# Supplementary material for: Endothelial derived, secreted long non-coding RNAs Gadlor1 and Gadlor2 aggravate cardiac remodeling
Source: Mol Ther Nucleic Acids. 2024 Aug 15;35(3):102306. doi: 10.1016/j.omtn.2024.102306 (PMC11402397; doi:10.1016/j.omtn.2024.102306)
Supplement: Document S1. Figures S1–S7, Tables S1–S5, and supplemental methods [file mmc1.pdf]

OMTN, Volume *35*

Supplemental information

Endothelial derived, secreted long non-coding RNAs *Gadlor1* and *Gadlor2* aggravate cardiac remodeling

Merve Keles, Steve Grein, Natali Froese, Dagmar Wirth, Felix A. Trogisch, Rhys Wardman, Shruthi Hemanna, Nina Weinzierl, Philipp-Sebastian Koch, Stefanie Uhlig, Santosh Lomada, Gesine M. Dittrich, Malgorzata Szaroszyk, Ricarda Haustein, Jan Hegermann, Abel Martin-Garrido, Johann Bauersachs, Derk Frank, Norbert Frey, Karen Bieback, Julio Cordero, Gergana Dobreva, Thomas Wieland, and Joerg Heineke

SUPPLEMENTAL MATERIAL

Extended Methods

Carotid flow measurement

To evaluate the degree of aortic constriction during TAC surgery, the peak velocity of flow in the right and left common carotid arteries (RCCA and LCCA) were measured 2 days after surgery with PW-Doppler at the level of carotid bifurcation to calculate the ratio of RCCA/LCCA flow.

EV FACS Staining

Characterization of surface markers of EVs was performed with flow cytometry by staining EC-derived EVs with CD9-PerCP-Cy5.5 (Miltenyi Biotec, 130-102-278, Clone: MZ3), CD63-APC (Miltenyi Biotec, 130-108-894, Clone: REA563) and CD54-FITC (BD, 553252, Clone: 3E2) labelled antibodies for 1 hour at 4°C. Measurements were obtained with BD FACSCanto ll.

EV Transmission Electron Microscopy (TEM)

Visualization of EVs was performed with TEM. After centrifugation, pellets were resuspended in the minimum possible volume of residual liquid and 25 % aqueous glutaraldehyde was added to a final concentration of 1 %. After fixation overnight, samples were mixed 1:1 (v/v) with 4 % agar at 40 °C. After hardening, the agar blocks were cut into cubes of 1 mm in size. Further preparation of samples and electron microscopy were performed as described, before being imaged with a FEI Morgagni 268 transmission electron microscope (FEI, Eindhoven, Netherlands) operated at 80 kV using a Veleta CCD camera (Olympus Soft Imaging Solutions).[1]

RNAScope in situ Hybridization

In situ hybridization was performed to localize *Gadlor1* (Mm-LOC118567341-C3), *Gadlor2* (Mm- AK038629-C1, Cat no: 404391) and *Cdh5* (Mm-Cdh5-C2, Cat no: 312531) mRNA expression on mouse heart tissue slides with RNAscope Multiplex Fluorescent v2 kit. The assay was performed according to the manufacturer's protocol. Briefly, OCT-embedded tissue slides (7 µm thickness) were used in the assay, fixed with 4% PFA and then stepwise dehydration was performed with increasing concentrations of ethanol. Tissue slides were subsequently treated with hydrogen peroxide (10 min at RT) and protease IV (30 min at RT) in the humidity control incubator. Slides were incubated for 2 hours with the mixture of probes to hybridize with the corresponding RNAs. Next, the signal was amplified with RNAScope Multiplex FL v2 AMP 1-3 reagents and then HRP channels were developed sequentially. Finally, DAPI was used to counterstain the slides, and each was mounted with ProLong Gold Antifade Mountant (Fisher Scientific).

Proximity Ligation Assay (PLA)

We performed proximity ligation assay (PLA) with minor modifications to a previously described protocol to confirm the interaction of *Gadlor1* and *Gadlor2* lncRNAs with CaMKII.[2] Briefly, specific single-stranded DNA probes, identical to those used in RAP-MS, were designed to be complementary to *Gadlor1* and *Gadlor2* RNA, each with a 3' biotin tag. For each target, five specific probes were pooled based on their region within the target RNA. HL-1 cells were fixed with 4% paraformaldehyde, washed in PBS, and permeabilized using PBS with 1% BSA and 0.1% Triton X-100. After washing in 0.1 M triethanolamine, acetic anhydride was incrementally added to a final concentration of 0.5%. Cells were washed in PBS with 0.02% Tween 20, followed by a hybridization buffer (5× SSC, 1× Denhardt's reagent, 0.1% Tween 20, 0.1% CHAPS, 5 mM EDTA, 1 mg/mL RNase-free tRNA, and 100 µg/mL heparin). Probes (200 nmol/L) were boiled at 95°C and incubated with cells in hybridization buffer overnight at 37°C in a humidified chamber.

Slides were sequentially washed in: 50% deionized formamide/5× SSC, 25% deionized formamide/1× SSC, 12.5% deionized formamide/2× SSC, 2× SSC/0.1% Tween 20, and 0.2× SSC/0.1% Tween 20. After washing in PBS with 0.02% Tween 20, slides were blocked with Duolink blocking buffer.

Primary antibodies (Rabbit-anti CamkIIΔ) were diluted in Duolink antibody diluent and incubated overnight at 4°C. After washing with Duolink buffer A, Duolink probes (Rabbit MINUS and Mouse PLUS) were applied to the slides for 1 hour at 37°C. Slides were washed in buffer A, treated with Duolink ligase mix for 30 minutes at 37°C, followed by a polymerase/ amplification mix for 100 minutes at 37°C. Finally, slides were washed in buffer B, rinsed in 0.01% buffer B, and mounted in Vectashield Mounting Medium with DAPI. The specificity of PLA was confirmed by omitting the primary antibody.

Supplemental Tables

Table S1: Clinical information of human serum samples from patient cohort.

Patient No	Age	Sex	Diagnosis	Previous Cardiac Surgery	NYHA Classification* During Admission	LVEF (%)
1	77	Female	AS, Hypertension, Dyslipidaemia, COPD	No	3	40
2	73	Male	AS, Hypertension, CAD, COPD	No	3	40
3	90	Female	AS, Hypertension, CAD	No	3	45
4	86	Female	AS, Hypertension	No	2	55
5	80	Male	AS, CAD	No	3	35
6	91	Female	AS, Hypertension, Dyslipidaemia, CAD, PAD	No	3	60
7	75	Male	AS, Hypertension, Diabetes, Dyslipidaemia	No	3	50
8	86	Female	AS, Hypertension, COPD	No	3	45
9	83	Female	AS, Hypertension, Dyslipidaemia, CAD	No	3	65
10	76	Male	AS, CAD	No	4	30
11	84	Male	AS, Hypertension, Diabetes, Dyslipidaemia, CAD, COPD	Yes	3	40
12	85	Male	AS, Hypertension	No	3	20
13	80	Male	AS, Dyslipidaemia, CAD	No	3	25
14	76	Male	AS, Dyslipidaemia, CAD	No	3	45
15	76	Female	AS, Hypertension, Diabetes, Dyslipidaemia, CAD	No	3	40
16	82	Female	AS, Hypertension, Diabetes, CAD	No	3	65

*New York Heart Association (NYHA) Functional Classification Criteria Committee, New York Heart Association, Inc. Diseases of the Heart and Blood Vessels. Nomenclature and Criteria for diagnosis, 6th edition Boston, Little, Brown and Co. 1964, p 114.
AS: Aortic Stenosis, CAD: Coronary Artery Disease, COPD: Chronic Obstructive Pulmonary Disease, LVEF: Left ventricle ejection fraction, PAD: Peripheral Arterial Disease.

Table S2: List of primers used for genotyping of *Gadlor*-KO mouse line.

	Primer 1 (Forward)	Primer 2 (Reverse)
***Gadlor*-WT**	CTTGAGCCGTCTCTCCAAAG	GGGTGGCATGCAAGATGATTGAGA
***Gadlor*-KO**	CTTGAGCCGTCTCTCCAAAG	TGTGGAGTGGACACATAGAGG

Table S3: List of antibodies/reagents used in cell isolation methodologies and immunofluorescence (IF) staining.

Target	Company, Catalogue No	Application
**Anti-mouse CD146 (LSEC) MicroBeads**	Miltenyi-Biotec, 130-092-007	Cell isolation
**Feeder Removal MicroBeads, Mouse**	Miltenyi-Biotec, 130-095-531	Cell isolation
**Isolectin B4 (IB4)**	Vector Lab, FL-1201	1:50 (IF)
**Purified Rat Anti-mouse CD102**	BD Pharmingen, 553326	Cell isolation
**Purified Rat Anti-mouse CD31**	BD Pharmingen, 553370	Cell isolation
**Rabbit Polyclonal anti-ki67**	Abcam, ab15580	1:100 (IF)
**VECTASHIELD HardSet Antifade Mounting Medium With DAPI**	Vector Lab, H-1500	
**Wheat Germ Agglutinin (WGA)**	Invitrogen, W21405	1:100 (IF)

Table S4: List of primers used for qPCR and qRT-PCR (5´-3´).

	Primer 1 (Forward)	Primer 2 (Reverse)
Gadlor1	AGGTGAGCTCTGGTTGTGTT	CTGCTGCCTGTGAAAGATGG
Gadlor2	TGAGACTCCACTTGCCACAT	TGTGGTTTCAGGCATGTTTCT
GADLOR1	AATTTCAGCCACAAGCATCC	TGCTTGGGGAAGAGGAAGTA
GADLOR2	TGGGATCTAAGCACTGACACC	GAGACAGACATTCGTTTGGTCA
**18S**	GTAACCCGTTGAACCCCATT	CCATCCAATCGGTAGTAGCG
**U6**	CTCGCTTCGGCAGCACA	AACGCTTCACGAATTTGCGT
Gapdh	CCGCATCTTCTTGTGCAGT	CATCACCTGGCCTACAGGAT
Acat1	GCAGGGAAGTTTGCCAGTGAGA	GAACACGGTCTTGAGCTTTGGC
Actn2	CACCTGGAGTTTGCCAAGAGAG	GCCTTGAACTGCTCATGTGCAG
Adam8	TGCCAACGTGACACTGGAGAAC	GCAGACACCTTAGCCAGTCCAA
Angpt2	AACTCGCTCCTTCAGAAGCAGC	TTCCGCACAGTCTCTGAAGGTG
Angptl4	CTGGACAGTGATTCAGAGACGC	GATGCTGTGCATCTTTTCCAGGC
Aurkb	CTTCTACGACCAGCAGAGGATC	GGCATCTGACAGTTCCTCCATG
Cacna1c	CGTTCTCATCCTGCTCAACACC	GAGCTTCAGGATCATCTCCACTG
Camk2d	GTGACACCTGAAGCCAAAGACC	CCTGTGCATCATGGAGGCAACA
Cdk1	CATGGACCTCAAGAAGTACCTGG	CAAGTCTCTGTGAAGAACTCGCC
Col15a1	ACACCCACAGTGACTCCCAAGA	TCCTCATTGCCCACGATGTCTC
Col1a1	CCGCTGGTCAAGATGGTC	CCTCGCTCTCCAGCCTTT
Col3a1	ATAAGCCCTGATGGTTCTCG	ATGCATGTTTCCCCAGTTTC
Col4a1	ATGGCTTGCCTGGAGAGATAGG	TGGTTGCCCTTTGAGTCCTGGA
Col6a1	GACACCTCTCAGTGTGCTCTGT	GCGATAAGCCTTGGCAGGAAATG
Comp	GTGCCCAACTTTGACCAGAGTG	ACAGGCATCACCCACAAAGTCG
Cox5a	GTCACACGAGACAGATGAGGAG	CCGTCTACATGCTCGCAATGCA

Cxcl2	CATCCAGAGCTTGAGTGTGACG	GGCTTCAGGGTCAAGGCAAACT
Dll1	GCTGGAAGTAGATGAGTGTGCTC	CACAGACCTTGCCATAGAAGCC
Efna1	GCTGAAGGTGACTGTCAATGGC	CGGCACTGTAACCAATGCTGTG
Fgfr2	GTCTCCGAGTATGAGTTGCCAG	CCACTGCTTCAGCCATGACTAC
Fh1	GAACTCACACGCAGGATGCTGT	GGCGGCTTTTATTCTCACCATCG
Fn1	TGTGACAACTGCCGTAGACC	TGGGGTGTGGATTGACCTTG
Gata4	GCCTCTATCACAAGATGAACGGC	TACAGGCTCACCCTCGGCATTA
Icam5	ACCGATGCACAGCAGTCAATGG	ATGTTCTGGGCAGCCTACACTG
Igf1	GTGGATGCTCTTCAGTTCGTGTG	TCCAGTCTCCTCAGATCACAGC
Il6	CGGCCTTCCCTACTTCACAA	TCCAGTTTGGTAGCATCCATCA
Klf15	ACACCAAGAGCAGCCACCTCAA	GCCTTGACAACTCATCTGAGCG
Lsamp	GGAGTCGAAGAGCAACGAAG	AATCTCAAGGCCATTTGCAC
Mfn2	GTGGAATACGCCAGTGAGAAGC	CAACTTGCTGGCACAGATGAGC
Myh6	GAGTGGGAGTTTATCGACTTCG	CCTTGACATTGCGAGGCTTC
Myh7	ACTGTCAACACTAAGAGGGTCA	TTGGATGATTTGATCTTCCAGGG
Nppa	TTCCTCGTCTTGGCCTTTTG	CCTCATCTTCTACCGGCATC
Nppb	GTCCAGCAGAGACCTCAAAA	AGGCAGAGTCAGAAACTGGA
Rcan1.4	CTTGTGTGGCAAACGATGATG	TGGTGTCCTTGTCATATGTTCTG
Sirt1	GGAGCAGATTAGTAAGCGGCTTG	GTTACTGCCACAGGAACTAGAGG
Sucla2	GGTGTCTCTGTTCCCAAAGGCT	TTTCCTCTGCCGCCAGCCAAAA
Tlr9	GCTGTCAATGGCTCTCAGTTCC	CCTGCAACTGTGGTAGCTCACT
ND1	CTAGCAGAAACAAACCGGGC	CCGGCTGCGTATTCTACGTT
16S	CCGCAAGGGAAAGATGAAAGAC	TCGTTTGGTTTCGGGGTTTC
**Gadlor_Intron**	GCTGCACAAATCCCACTTCA	TGAAGTGGGATTTGTGCAGC
**Gadlor_Spanning**	AGCACATTGTACTCCCATGTT	GTGAGTCCCTGCAAGTCCTA

**Gadlor1_ExonIntron**	AGCACATTGTACTCCCATGTT	GGCAGTTTGGAAGGTGATCA
**Gadlor2_ExonIntron**	TGGGAAGTAGAGAGCTATTGTTC	TCCTATGTGTGCACTGTCCA

Table S5: List of *Gadlor1* and *Gadlor2* probes used for RNA antisense purification (RAP).

	**Probe Sequence**
***Gadlor1* – Probe 1**	[Btn]AGGATTGTTAAATATGACTATGCTTGGTATAGTCACAAAACATGGGAGTAC
***Gadlor1* – Probe 2**	[Btn]TATCATAATCTTTCTGTAGGCCATTACTTGTTTCATATTTTAAAGGGACAGTCCACTCTAGGAATGTCAAGTGTCTGATCTCTGAAAACA
***Gadlor1* – Probe 3**	[Btn]TTTTCTGAATAGTTGAAAATTCTAACTAAACACAGGAAGAACAGAGNCACAAGAATAAAGAAATTTAGATATATCCTAAATGTTTCCAGG
***Gadlor1* – Probe 4**	[Btn]ATAAAAGAAGGCGAGGGGTGGCATGCAAGATGATTGAGAAAGCCCAGTAGCCATTTTTGGGGTGGGGCAAAGGGAGTGGTCTGGGTAGGG
***Gadlor2* – Probe 1**	[Btn]GCTGCTATTTTATTATCTCTTTGGTTCTGTTTTTCATTTGTATTAAAAATG
***Gadlor2* – Probe 2**	[Btn]GTGATGGTGAAGATGAAATTGAGATGAATCATTTGAAGAACGATGTGCGTTTTAGAAGAATCACTTTGTC

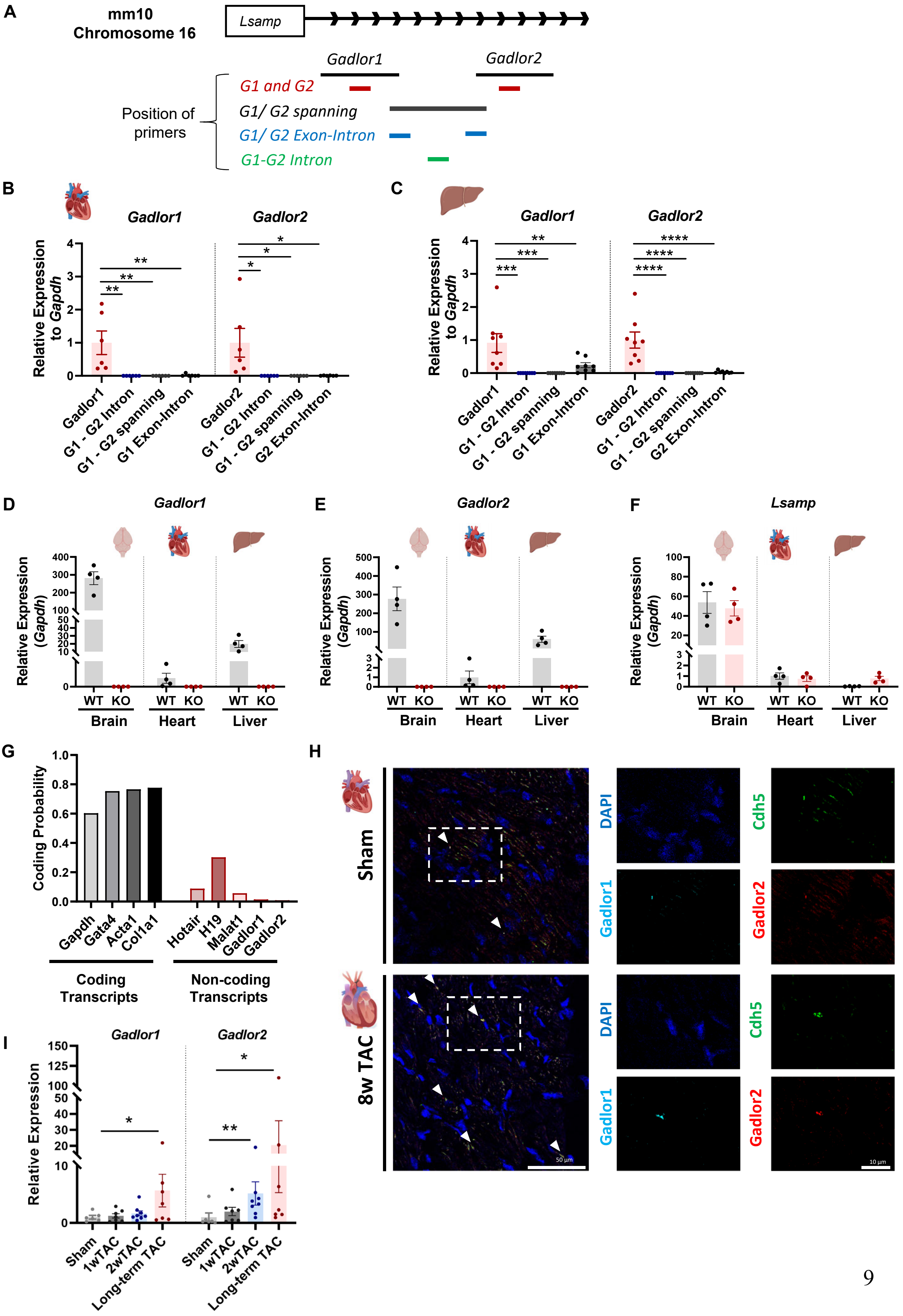
A
mm10
Chromosome 16
Lsamp
Gadlor1
Gadlor2
Position of primers
G1 and G2
G1/ G2 spanning
G1/ G2 Exon-Intron
G1-G2 Intron
B
Gadlor1
Gadlor2
Relative Expression to Gapdh
Gadlor1
G1 - G2 Intron
G1 - G2 spanning
G1 Exon-Intron
Gadlor2
G1 - G2 Intron
G1 - G2 spanning
G2 Exon-Intron
C
Gadlor1
Gadlor2
Relative Expression to Gapdh
D
Gadlor1
Relative Expression (Gapdh)
WT KO
Brain
Heart
Liver
E
Gadlor2
F
Lsamp
G
Coding Probability
Gapdh
Gata4
Acta1
Col1a1
Hotair
H19
Malat1
Gadlor1
Gadlor2
Coding Transcripts
Non-coding Transcripts
H
Sham
8w TAC
DAPI
Cdh5
Gadlor1
Gadlor2
50 µm
10 µm
I
Gadlor1
Gadlor2
Relative Expression
Sham
1wTAC
2wTAC
Long-term TAC

Figure S1: *Gadlor1* and *Gadlor2* are non-coding transcripts that are located in the intronic region of the *Lsamp* gene.

A. Position of primer pairs targeting different regions within *Gadlor1* and *Gadlor2* lncRNAs. **B-C.** Quantitative RT-PCR analysis of *Gadlor1/2* expression in heart and liver tissues with indicated primer pairs. **D-F.** Systemic deletion of the whole region containing *Gadlor* lncRNAs did not affect the expression of the neighbor gene *Lsamp*, which is mainly expressed in brain tissue (*Gapdh* expression used for normalization). Relative expression of *Gadlor1* (D), *Gadlor2* (E) and *Lsamp* (F) were shown in mouse brain, heart and liver tissue samples collected from WT and Gadlor-KO mice (n=4, same samples were indicated in each graph). **G.** Coding probability of *Gadlor1* and *Gadlor2* was evaluated with the online Coding Potential Assessment Tool (CPAT - http://lilab.research.bcm.edu/) and compared with coding transcripts and well-known lncRNAs for validation that revealed Gadlor1 and Gadlor2 are non-coding transcripts. **H.** Fluorescent in situ hybridization of *Gadlor1* (cyan), *Gadlor2* (red) and *Cdh5* RNAs in mouse heart tissue sections of sham and following 8-weeks of TAC surgery. *Cdh5* shows endothelial cells (green), nuclei stained with DAPI (blue), and the overlap in 3 channels was indicated with arrows. Dashed rectangles were the zoomed areas showed in separate pictures. Scale bars: 50 μm and 10 μm, as indicated. **I.** *Gadlor1/2* lncRNA levels were assessed in mouse cardiac ECs at different time-points post-TAC compared to sham samples. Sham group (n=6), 1-week TAC (n=7), 2-week TAC (n=8), Long-term TAC (10-12 weeks, n=7). The housekeeping gene 18S was used for normalization of RT-qPCR data. Data are shown as mean±SEM. *p-value<0.05, **p-value<0.01, ***p-value<0.001; ****p-value<0.0001

A **AK037972 *(Gadlor1)***

```
#=======================================
# Aligned_sequences: 2
# Gap_penalty: 10.0
# Extend_penalty: 0.5
#
# Length: 578
# Identity:      268/578 (46.4%)
# Similarity:    268/578 (46.4%)
# Gaps:          259/578 (44.8%)
# Score: 740.5
#=======================================
H   1 AAATTGTCTAGAA-TGTGGCAGTTTAAAAACACATCTACATTGATCTACA     49
        |||||||.||||| |||||||.|||||||.|||.||..|||||||||||.
M   1 AAATTGTTTAGAACTGTGGCACTTTAAAAGCACCTCGGCATTGATCTACT     50

   50 GTCTCAAGGTCCCAC--CTTTGTGTTACTTTTAGAATTTGTAGATGGATG     97
      ..|||.||.||.|||  ||.||||.|||||||||||.|||||||||||||.
   51 AACTCTAGATCTCACCTCTCTGTGCTACTTTTAGAGTTTGTAGATGGATA    100

   98 TTATTCAGCA-AGGATGACTCATCTACATT-TGGTTTGTCAATTTGTTTT    145
      .|.|.||||| |||| ||.||||||||.|| ||  .||||.|||||||||
  101 CTGTCCAGCAGAGGA-GAGTCATCTACGTTCTG--ATGTCGATTTGTTTT    147

  146 CAAAGATCATA----TGTACATAGCGTCCT----TCGATCTTACTTTCCC    187
      ||.||||||.|    || ||||    ||||    |.|     |||.||||
  148 CAGAGATCAGACACTTG-ACAT----TCCTAGAGTGG-----ACTGTCCC    187

  188 TTTAAAATATGAAACTGATAATGACCTTGAAAAAGAT-------AGAATT    230
      |||||||||||||||...|||||.|||..|.||||||       |.|.||
  188 TTTAAAATATGAAACAAGTAATGGCCTACAGAAAGATTATGATAAAACTT    237

  231 TC----------------------------------------AG------    234
      ||                                        ||
  238 TCCAGTTTTTTGGCTTTTCCCTTCGGCCCTGACTGGATGTGTAGTCTACT    287

  235 --CCACAA------------------------------------------    240
        ||||||
  288 TCCCACAATCCTGTATGTTACATGGTTCCCTACCCAGACCACTCCCTTTG    337

  241 -----------------------------------------GCATCCCA-    248
                                               ||||.|||
  338 CCCCACCCCAAAAATGGCTACTGGGCTTTCTCAATCATCTTGCATGCCAC    387

  249 ---------------------------------TGAA-------AT----    254
                                       ||||       ||
  388 CCCTCGCCTTCTTTTATGTTTCAGAGTTGTGTGAAGAATCACATTAGT    437

  255 --TAATGTATCCATACTTTTAACA--CTA-----GTATAC-----TTTAT    290
        ||.|||||.|.|.||||| |||  |||     ||.|||     |||||
  438 GATACTGTATGCTTTCTTTT-ACAATCTATTTACGTTTACACATATTTAT    486

  291 CTTCTTATTT-CA-AATGCTC--TGTCTCC--------------------    316
      .||..||||| || |.|.|||  |||.|.|
  487 TTTTATATTTACATATTTCTCATTGTTTACCAGTTGGCAATAGTGATATT    536

  317 TT---------CTGTCCA--TTACTTCC    333
      ||         ||.|.||  ||||||||
  537 TTGTGAGCTGACTTTACATTTTACTTCC    564
```

B **AK038629 (*Gadlor2*)**

```
#=======================================
# Aligned_sequences: 2
# Gap_penalty: 10.0
# Extend_penalty: 0.5
#
# Length: 913
# Identity:      656/913 (71.9%)
# Similarity:    656/913 (71.9%)
# Gaps:          130/913 (14.2%)
# Score: 2336.5
#=======================================
H   1 AAAAAGAAAATTTGGA-AGATGCTCAAATAGGA-TTGATAGAGGTTCATT     48
        ||.||.|||||||||| || |||.||.|||||| ||| .||.|..|||..
M  34 AAGAATAAAATTTGGACAG-TGCACACATAGGACTTG-CAGGGACTCACA     81

   49 TTGTGTCATATTTT-CGCTTTATATGGATGTTGATATGTTCTCAGAATAA     97
      .||||||||||||| |.||||.||||.|||||||||||||.||||||| |
   82 GTGTGTCATATTTTGCACTTTTTATGAATGTTGATATGTTTTCAGAAT-A    130

   98 ATTTATAACTGCAATAGAAAATGGAATGACTATTGATTATTTTTTAGTTA    147
      |||||||||||||||.|||||..|||||.||||||||||..||||||||.
  131 ATTTATAACTGCAATTGAAAACTGAATGGCTATTGATTAAATTTTAGTTT    180

  148 CCCAAACTAATTGTCATATAAAGCTTTGTAAAT-CTTTTA-AAATGAGTA    195
      |..||||||||||.||||||||||||..||||| |||||| |||  ||.|
  181 CAAAAACTAATTGACATATAAAGCTTCATAAATCCTTTTAGAAA--AGGA    228

  196 TGGGTGTAAATTAAAAACGAATTAAAGCAGAGGCTTATAAAGACACT---    242
      ||.||.||||||||||||.|||||.||||.||||||||||| |.|||
  229 TGAGTATAAATTAAAAACTAATTACAGCAAAGGCTTATAAA-ATACTCCA    277

  243 ATCTCCCCTTCAATGGGTTATGTATTTTTGTTGTGTGTTAGGAAGATATC    292
      || |||..|||.|| ||||||.||   .||||||.||.||.||||.|.|.
  278 AT-TCCAGTTCTAT-GGTTATATA---CTGTTGTATGCTATGAAGGTGTT    322

  293 TCTTGATCTCCACACCCAAAACTCCTTCC----------AAATGAACAAA    332
      .|||..|||.|..|..|||||||||.||          |.||||||
  323 CCTTTGTCTTCTTAATCAAAACTCCTGCCATAGAATTAAATATGAAC---    369

  333 GGCCTACACACAAAAATTATTTCAATTGCTAATAGCCAGTTTTTAT----    378
              ||.||||..|||||.||||||||||....|.|||.|||
  370 --------CATAAAAGATATTTTAATTGCTAATGATTACTTTATATATAG    411

  379 ------TTTTCAAAAGTAAATGCTTTCTTTTTTTGAAAAGTAAATATAAC    422
            |||||||.||                      |||  .|||||.
  412 TATCCCTTTTCAAGAG----------------------AGT--TTATAAG    437

  423 TATTTTAACAAAGTAAGATTTAAAAA---AAAAAACAACTCAGAAAAAAG    469
      .|||||.|...|||||.|||||||||   ||||||.|||      |.|||
  438 AATTTTTAATCAGTAATATTTAAAAATTTAAAAAAGAAC------AGAAG    481

  470 T--GCCAT------GCAG-AGATAACAGGGCTGATCTGTCTGTTATCTGC    510
      |  |||||      |||| ||| |.||      || ||.|.||||||||.
  482 TGGGCCATGTTAAGGCAGCAGA-ACCA------AT-TGCCAGTTATCTGT    523

  511 AGCCTCTATCCTTACTGTTAACAAGCCTTTTATCTTTGAAGACACTAAAC    560
      |.|||||||||||..||.|||.||||||||||.|||.|||||||||||.|
  524 AACCTCTATCCTTGATGCTAATAAGCCTTTTACCTTCGAAGACACTAAGC    573

  561 ATCTGGGATCTAAGCACTGACACCTATTAGTTACAGTGGTTTCCTTTTAC    610
      ||||||||||||||.|.||||||||||||||||||.||||||||||||||
  574 ATCTGGGATCTAAGTAGTGACACCTATTAGTTACAATGGTTTCCTTTTAC    623

  611 CTTTCTAAACTATCTGATAGATAAACCTCCAGGAATCCTACAAAATTAGG    660
      | ||||||.|.|||||||||||||||||||||||||||.||||||||.|.
  624 C-TTCTAAGCAATCTGATAGATAAACCTCCAGGAATCCCACAAAATTGGA    672

  661 CCCTTAATTGACCAAACGAATGTCTGTCTCTCTTTTTCAATCTAAATCAA    710
      ||||||||||||||||.|||||.|||||||||||||.||.|.|||||..|
  673 CCCTTAATTGACCAAATGAATGGCTGTCTCTCTTTTACAGTATAAATTGA    722

  711 AAAG-GAGTTTGCTCCTAGGAG--ATGAGATGATGTACAGTGGAAAAAAA    757
      |||| |||||||||||.|.|||  |.||.||.|..||    |||||.|||
  723 AAAGTGAGTTTGCTCCCATGAGGTAAGACATAACTTA----GGAAAGAAA    768

  758 TATTTCA-GCTGCAACTCCACTGGTAACCGTCAATGTGACCTTATAATCT    806
          ||| ||||..||||||||.|..| |.|.|.||||.||||.||||||
  769 ----TCACGCTGAGACTCCACTTGCCA-CATTAGTGTGCCCTTGTAATCT    813

  807 CCCC-----CCAATTTATTCCCA-AAACAGCTA--CCAAAGTGATCTTTC    848
      |.||     |||       |||| .||||| ||  .|||||||||..|||
  814 CACCAAAAGCCA-------CCCACCAACAG-TAGGACAAAGTGATTCTTC    855

  849 TAAAACCCACATC    861
      ||||||.||||||
  856 TAAAACGCACATC    868
```

C

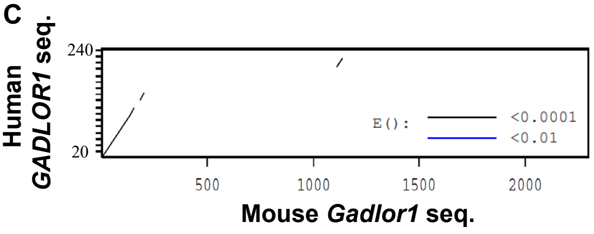

D

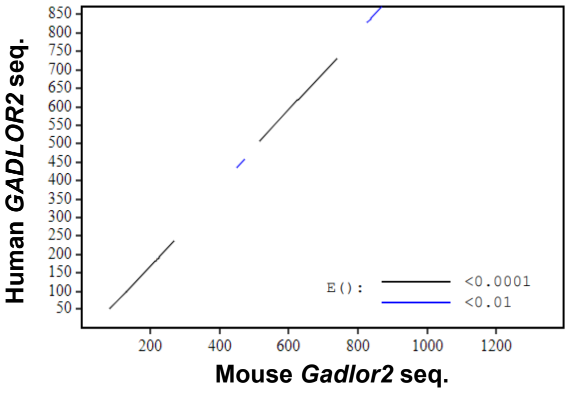

Figure S2: Conservation levels of Gadlor1/2 between mouse and human genome.
A-B. Assessment of sequence level conservation of *Gadlor1* and *Gadlor2* among human (H) and mouse (M) genome evaluated with EMBOSS-Water local alignment tool (https://www.ebi.ac.uk/Tools/psa/emboss_water/).
C-D. Visualization of pairwise local alignment with LALIGN DNA:DNA tool of University of Virginia (https://fastademo.bioch.virginia.edu/fasta_www2/fasta_www.cgi?rm=lplalign).

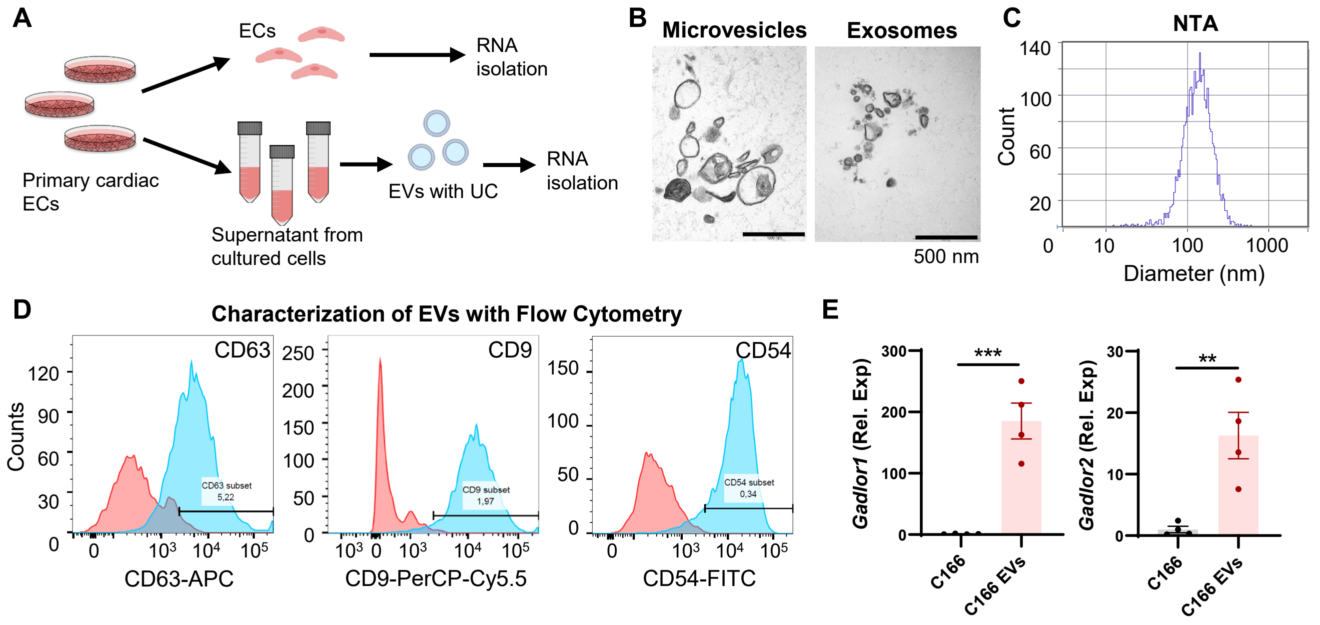

Figure S3: ***Gadlor*** **lncRNAs are mainly secreted in endothelial cells (EC) derived extracellular vesicles (EVs). A.** Scheme depicting the experimental design of EV isolation with ultracentrifugation (UC) from cultured primary cardiac ECs. Characterization of cardiac EC-derived EVs by **B.** visualizing microvesicles and exosomes with electron microscopy (scale bar: 500 nm) and **C.** detecting the size in diameter (nm: nanometers) with Nanoparticle Tracking Analysis (NTA). **D.** Flow cytometry analysis of EV surface markers (CD63-APC, CD9-PerCP-Cy5.5 and CD54-FITC) on EC-derived EVs that were stained against markers (blue curve) and compared to isotype control (red curve). **E.** Expression of *Gadlor1* and *Gadlor2* in C166 mouse endothelial cell and in EVs derived from these cells. Data are shown as mean±SEM. Data normality was evaluated with Shapiro- Wilk test and p-values were calculated with Student's t-test for parametric (or Mann-Whitney for non-parametric) assessment. *p-value<0.05, **p-value<0.01, ***p-value<0.001.

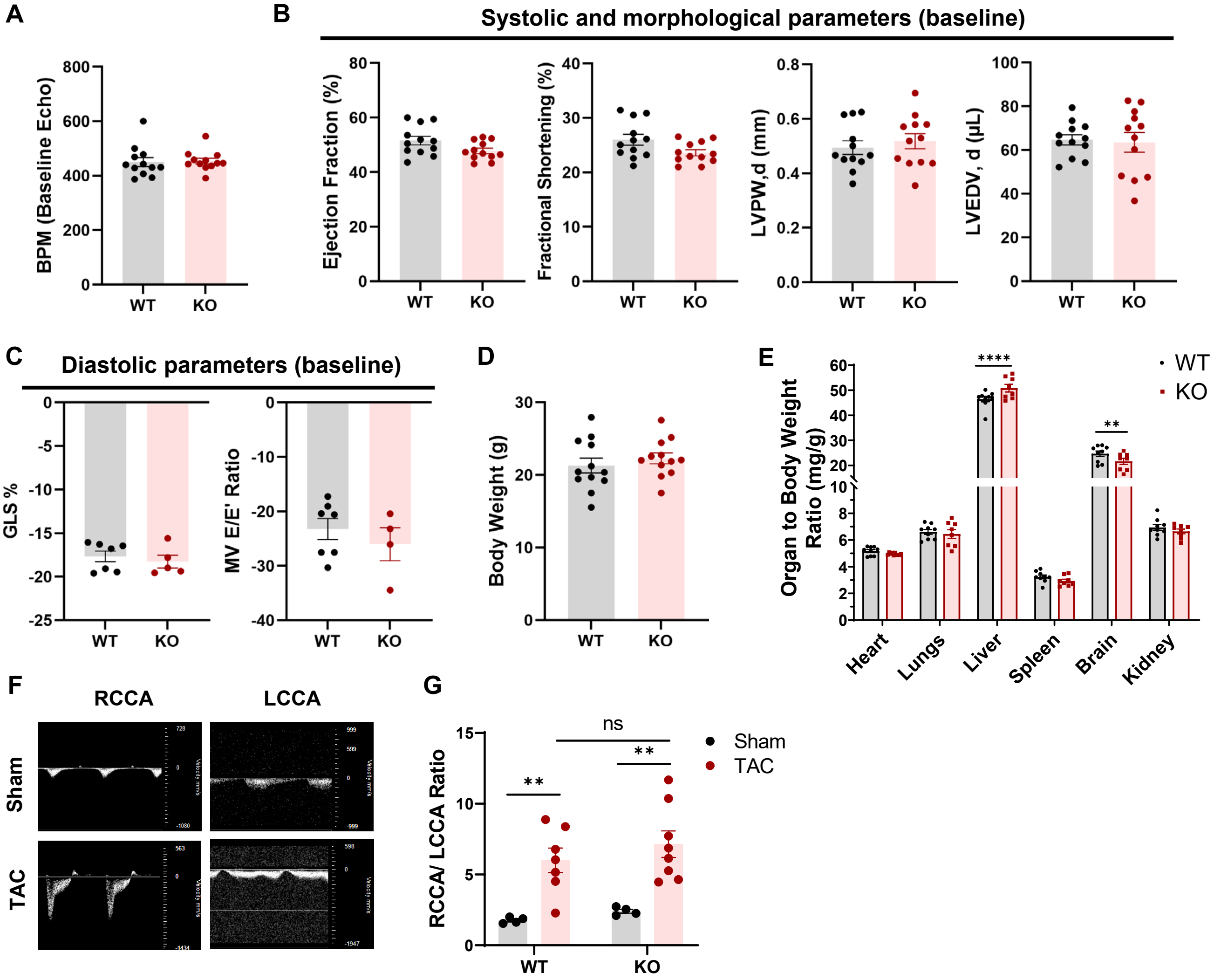

Figure S4: Baseline phenotypic characterization of *Gadlor*-KO mice compared to wild-type (WT) littermates.

A. Heart rate of WT and *Gadlor*-KO mice during echocardiography (BPM: beats per minute, n≥12). **B.** Analysis of systolic function and morphological parameters of left ventricle (LV) ejection fraction (%), fractional shortening (%), LV posterior wall thickness in diastole (mm) and LV end diastolic volume (µl) (n≥12). **C.** Analysis of diastolic function parameters, global longitudinal strain (GLS, %) and mitral valve (MV) E to E' ratio (n≥4). **D.** Body weight (g) of adult (9 weeks old) WT and *Gadlor*-KO animals (n≥18), and **E.** organ to body weight ratio (mg/g) of isolated organs including heart, lungs, liver, spleen, brain and kidney (n≥8). **F-G.** Representative images and quantification of flow measurement in right and left common carotid arteries (RCCA and LCCA) in sham (n≥4) and TAC (n≥7) mice to measure the strength of aortic constriction in WT and *Gadlor*-KO animals.

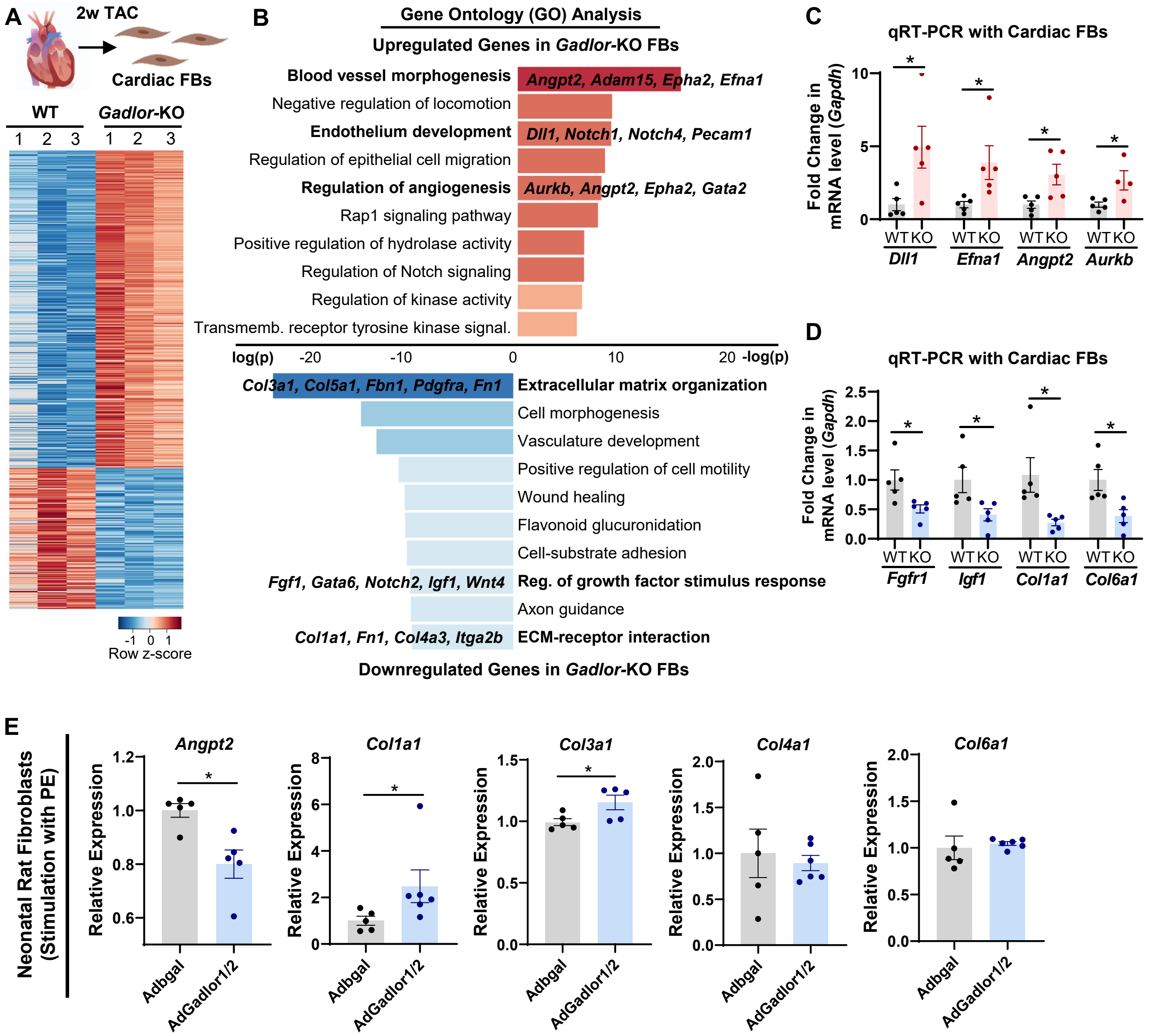

Figure S5: RNA sequencing of *Gadlor*-KO cardiac FBs showed less induction of fibrosis-associated genes after TAC.

A. Heatmap showing differentially regulated genes analysed by bulk RNAseq of isolated cardiac fibroblasts after 2-weeks TAC. **B.** Bar plots showing the gene-ontology (GO) analysis of upregulated and downregulated genes in *Gadlor*-KO FBs after 2-weeks TAC (Red: Upregulated in *Gadlor*-KO, Blue: Downregulated in *Gadlor*-KO). Exemplary genes were listed for selected GO-terms. **C-D.** Validation of selected genes from RNAseq data with qRT-PCR in isolated adult cardiac FBs after 2 weeks of TAC (Red: Upregulated in *Gadlor*-KO, Blue: Downregulated in Gadlor-KO). **E.** qRT-PCR of selected genes in neonatal rat fibroblasts (NRFB) overexpressing *Gadlor* lncRNAs after *Gadlor1/2* adenovirus treatment (βgal as control) followed by phenylephrine (PE) stimulation (100 µM, 24 hours). Data are shown as mean±SEM. Data normality was evaluated with Shapiro-Wilk test and p- values were calculated with Student's t-test for parametric and Mann-Whitney test for non-parametric assessment. *p- value<0.05.

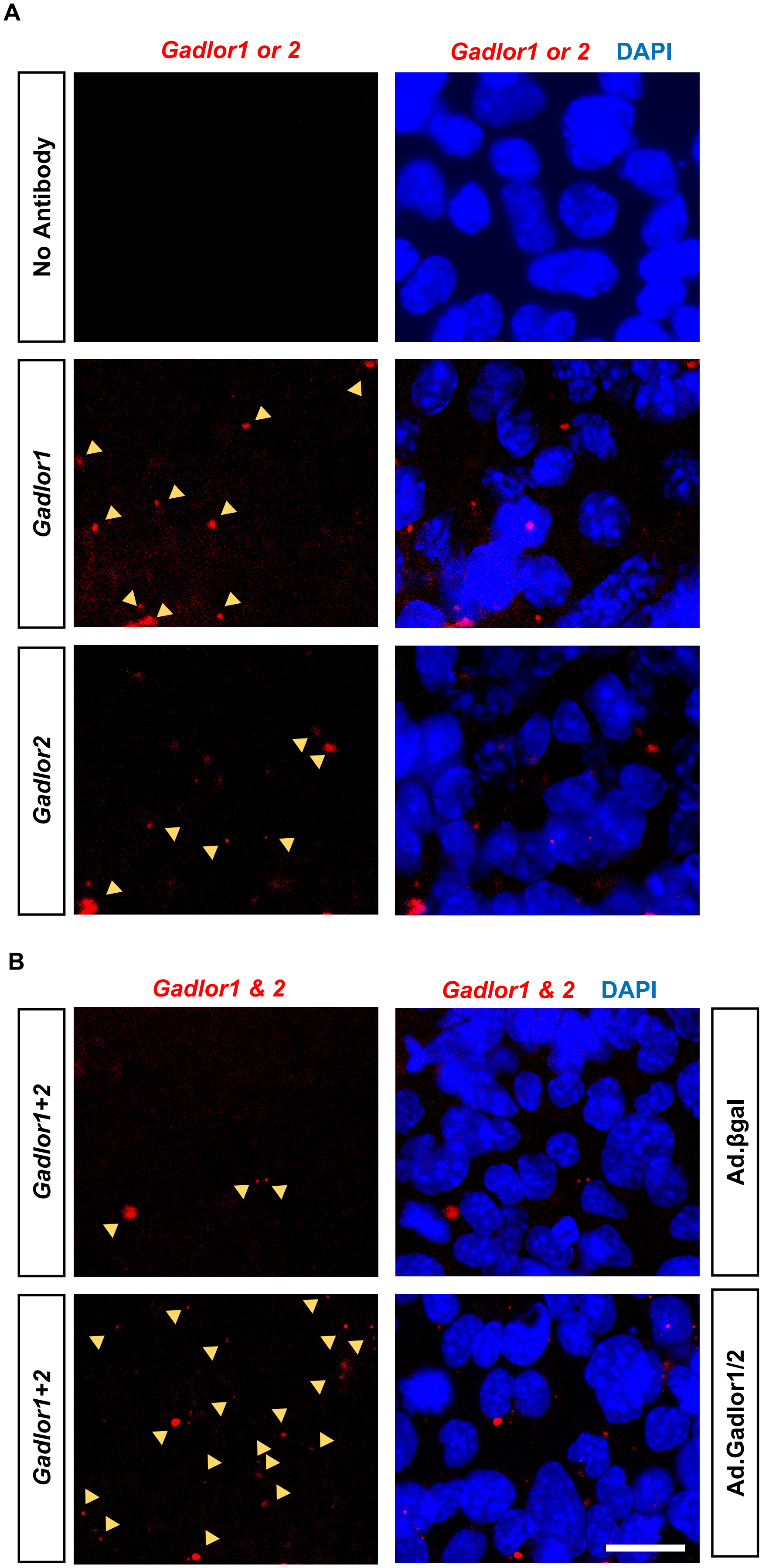
A
Gadlor1 or 2
Gadlor1 or 2 DAPI
No Antibody
Gadlor1
Gadlor2
B
Gadlor1 & 2
Gadlor1 & 2 DAPI
Gadlor1+2
Ad.βgal
Gadlor1+2
Ad.Gadlor1/2

Figure S6: Visualization of the specific interactions between CaMKII and *Gadlor1* and *Gadlor2* lncRNAs in situ by proximity ligation assay (PLA).

A. Representative images from proximity ligation assays (PLA) in HL1 cardiomyocytes in situ, showing the interaction between CaMKII with endogenous *Gadlor1* or *Gadlor2* lncRNAs (red). PLA specificity was confirmed by omitting the primary antibody as a negative control. **B.** Representative images from PLA in HL1 cardiomyocytes in situ, showing the interaction between CaMKII with adenoviral overexpressed *Gadlor1* and *Gadlor2* lncRNAs (red). Cells were mounted with Vectashield containing DAPI (blue). Scale bar: 25 µm.

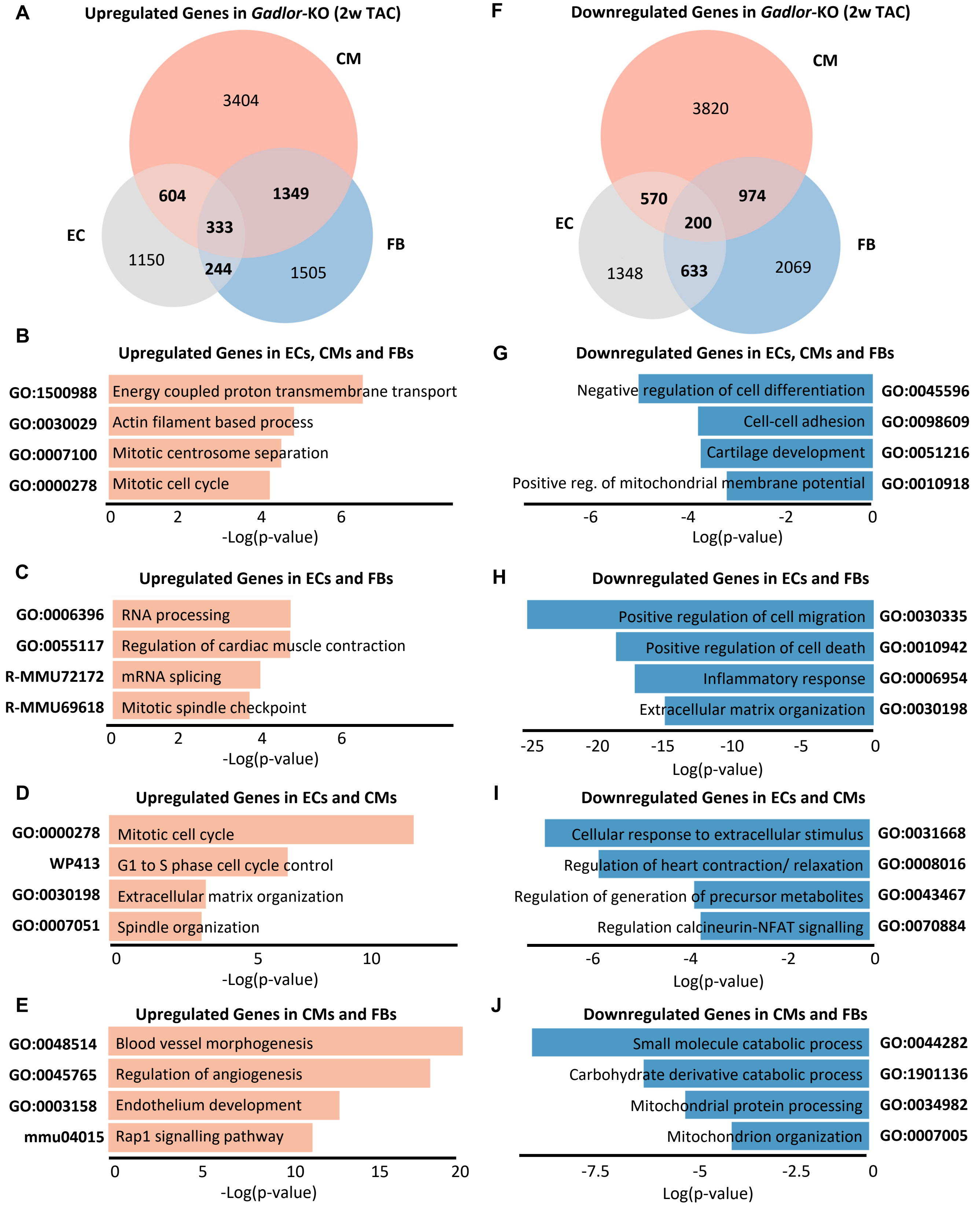
A
Upregulated Genes in Gadlor-KO (2w TAC)
CM
EC
FB
3404
604
1349
333
1150
244
1505
F
Downregulated Genes in Gadlor-KO (2w TAC)
CM
EC
FB
3820
570
974
200
1348
633
2069
B
Upregulated Genes in ECs, CMs and FBs
GO:1500988 Energy coupled proton transmembrane transport
GO:0030029 Actin filament based process
GO:0007100 Mitotic centrosome separation
GO:0000278 Mitotic cell cycle
-Log(p-value)
G
Downregulated Genes in ECs, CMs and FBs
Negative regulation of cell differentiation GO:0045596
Cell-cell adhesion GO:0098609
Cartilage development GO:0051216
Positive reg. of mitochondrial membrane potential GO:0010918
Log(p-value)
C
Upregulated Genes in ECs and FBs
GO:0006396 RNA processing
GO:0055117 Regulation of cardiac muscle contraction
R-MMU72172 mRNA splicing
R-MMU69618 Mitotic spindle checkpoint
-Log(p-value)
H
Downregulated Genes in ECs and FBs
Positive regulation of cell migration GO:0030335
Positive regulation of cell death GO:0010942
Inflammatory response GO:0006954
Extracellular matrix organization GO:0030198
Log(p-value)
D
Upregulated Genes in ECs and CMs
GO:0000278 Mitotic cell cycle
WP413 G1 to S phase cell cycle control
GO:0030198 Extracellular matrix organization
GO:0007051 Spindle organization
-Log(p-value)
I
Downregulated Genes in ECs and CMs
Cellular response to extracellular stimulus GO:0031668
Regulation of heart contraction/ relaxation GO:0008016
Regulation of generation of precursor metabolites GO:0043467
Regulation calcineurin-NFAT signalling GO:0070884
Log(p-value)
E
Upregulated Genes in CMs and FBs
GO:0048514 Blood vessel morphogenesis
GO:0045765 Regulation of angiogenesis
GO:0003158 Endothelium development
mmu04015 Rap1 signalling pathway
-Log(p-value)
J
Downregulated Genes in CMs and FBs
Small molecule catabolic process GO:0044282
Carbohydrate derivative catabolic process GO:1901136
Mitochondrial protein processing GO:0034982
Mitochondrion organization GO:0007005
Log(p-value)

Figure S7: Venn diagrams of differentially expressed genes in different cardiac cell types of *Gadlor*-KO mice compared to WT littermates after 2 weeks of TAC.
A. Venn diagram of upregulated genes in different cardiac cell types (CM: red, EC: grey, FB: blue) of *Gadlor*-KO mice compared to WT after 2-weeks TAC. The numbers indicating the number of genes in corresponding cell types. Genes were filtered as fold-change greater or equals to 1.2 for each condition. **B-E.** Gene ontology (GO) analysis of indicated intersections representing the overlapping upregulated genes in shown cell type of *Gadlor*-KO compared to WT littermates. **F.** Venn diagram of downregulated genes in different cardiac cell types (CM: red, EC: grey, FB: blue) of *Gadlor*-KO mice compared to WT after 2-weeks TAC. **G-J.** Gene ontology (GO) analysis of indicated intersections representing the overlapping downregulated genes in shown cell type of Gadlor-KO compared to WT littermates. Bar plots showing selected GO-terms for each condition.

Supplemental Video

Video S1: PKH67 labelled extracellular vesicles (EVs) taken up by a neonatal rat cardiomyocyte (NRCM) in a three-dimensional view.

Supplemental References

1. Grund A, Szaroszyk M, Korf-Klingebiel M, Malek Mohammadi M, Trogisch FA, Schrameck U, Gigina A, Tiedje C, Gaestel M, Kraft T, et al. TIP30 counteracts cardiac hypertrophy and failure by inhibiting translational elongation. EMBO Mol Med. 2019;11:e10018. doi: 10.15252/emmm.201810018

2. Wardman R, Keles M, Pachkiv I, Hemanna S, Grein S, Schwarz J, Stein F, Ola R, Dobreva G, Hentze MW et al. RNA-Binding Proteins Regulate Post-Transcriptional Responses to TGF-β to Coordinate Function and Mesenchymal Activation of Murine Endothelial Cells. Arterioscler. Thromb. Vasc. Biol. 2023; 43:1967–1989. doi.org/10.1161/ATVBAHA.123.319925
