# Supplementary material for: Endothelial derived, secreted long non-coding RNAs Gadlor1 and Gadlor2 aggravate cardiac remodeling
Source: Mol Ther Nucleic Acids. 2024 Aug 15;35(3):102306. doi: 10.1016/j.omtn.2024.102306 (PMC11402397; doi:10.1016/j.omtn.2024.102306)
Supplement: Document S2. Article plus supplemental information [file mmc3.pdf]

Molecular Therapy
Nucleic Acids
Original Article

Endothelial derived, secreted long non-coding RNAs *Gadlor1* and *Gadlor2* aggravate cardiac remodeling

Merve Keles,[1,2,3,14,16] Steve Grein,[1,3,16] Natali Froese,[4] Dagmar Wirth,[5] Felix A. Trogisch,[1,2,3] Rhys Wardman,[1,3] Shruthi Hemanna,[1,3] Nina Weinzierl,[1] Philipp-Sebastian Koch,[6] Stefanie Uhlig,[7] Santosh Lomada,[3,8] Gesine M. Dittrich,[1,3,15] Malgorzata Szaroszyk,[4] Ricarda Haustein,[4] Jan Hegermann,[9] Abel Martin-Garrido,[1] Johann Bauersachs,[4] Derk Frank,[10,11] Norbert Frey,[3,12] Karen Bieback,[7] Julio Cordero,[13] Gergana Dobreva,[3,13] Thomas Wieland,[3,8] and Joerg Heineke[1,2,3]

[1]ECAS (European Center for Angioscience), Department of Cardiovascular Physiology, Medical Faculty Mannheim of Heidelberg University, 68167 Mannheim, Germany; [2]CFPM (Core Facility Platform Mannheim), Cardiac Imaging Center, Medical Faculty Mannheim of Heidelberg University, 68167 Mannheim, Germany; [3]DZHK (German Center for Cardiovascular Research), partner site Heidelberg/Mannheim, 69120 Heidelberg, Germany; [4]Department of Cardiology and Angiology, Hannover Medical School, 30625 Hannover, Germany; [5]Helmholtz Center for Infection Research, Model Systems for Infection and Immunity, 38124 Braunschweig, Germany; [6]Department of Dermatology, Venereology and Allergology, University Medical Center and Medical Faculty Mannheim, Heidelberg University, 68167 Mannheim, Germany; [7]CFPM, FlowCore, Medical Faculty Mannheim of Heidelberg University, 68167 Mannheim, Germany; [8]ECAS, Department of Experimental Pharmacology, Medical Faculty Mannheim of Heidelberg University, 68167 Mannheim, Germany; [9]Institute of Functional and Applied Anatomy, Core Unit Electron Microscopy, Hannover Medical School, 30625 Hannover, Germany; [10]Department of Internal Medicine III, University Hospital Schleswig-Holstein, 24105 Kiel, Germany; [11]DZHK, partner site Hamburg/Kiel/Lübeck, 20246 Hamburg, Germany; [12]Department of Internal Medicine III, Medical Faculty Heidelberg, Heidelberg University, 69120 Heidelberg, Germany; [13]ECAS, Department of Cardiovascular Genomics and Epigenomics, Medical Faculty Mannheim of Heidelberg University, 68167 Mannheim, Germany

Pathological cardiac remodeling predisposes individuals to developing heart failure. Here, we investigated two co-regulated long non-coding RNAs (lncRNAs), termed *Gadlor1* and *Gadlor2*, which are upregulated in failing hearts of patients and mice. Cardiac overexpression of *Gadlor1* and *Gadlor2* aggravated myocardial dysfunction and enhanced hypertrophic and fibrotic remodeling in mice exposed to pressure overload. Compound *Gadlor1/2* knockout (KO) mice showed markedly reduced myocardial hypertrophy, fibrosis, and dysfunction, while exhibiting increased angiogenesis during short and prolonged periods of pressure overload. Paradoxically, *Gadlor1/2* KO mice suffered from sudden death during prolonged overload, possibly due to cardiac arrhythmia. *Gadlor1* and *Gadlor2*, which are mainly expressed in endothelial cells (ECs) in the heart, where they inhibit pro-angiogenic gene expression, are strongly secreted within extracellular vesicles (EVs). These EVs transfer *Gadlor* lncRNAs to cardiomyocytes, where they bind and activate calmodulin-dependent kinase II, and impact pro-hypertrophic gene expression and calcium homeostasis. Therefore, we reveal a crucial lncRNA-based mechanism of EC-cardiomyocyte crosstalk during heart failure, which could be specifically modified in the future for therapeutic purposes.

INTRODUCTION

Despite recent advances in therapeutic options, chronic heart failure is still one of the leading causes of global mortality, in part due to insufficient means to counteract maladaptive cardiac remodeling.[1–3] Cardiac remodeling comprises global changes in heart shape, reduced function and arrhythmia, which are the consequence of cardiomyocyte (CM) hypertrophy, altered angiogenesis, and myocardial fibrosis.[4–7] The majority of current therapeutic options target protein-coding genes; however, recent studies revealed the significance of non-coding transcripts in cardiovascular development and disease conditions.[5,8,9]

Long non-coding RNAs (lncRNAs) are a diverse group of non-coding transcripts that are longer than 200 nucleotides, and that in general do not encode for proteins.[10] Due to their complex structure, lncRNAs can act, for instance, as chromatin modifiers, affect protein functions, or alter mRNA stability or processing.[5] Thereby, lncRNAs typically affect gene expression, alternative splicing, or signaling pathways.[11,12]

Received 30 January 2024; accepted 12 August 2024;
https://doi.org/10.1016/j.omtn.2024.102306.

[14]Present address: National Heart and Lung Institute, Faculty of Medicine, Imperial College London, W12 0NN London, UK

[15]Present Address: Department of Pharmacology and Toxicology, University Medical Center Göttingen, 37075 Göttingen, Germany

[16]These authors contributed equally

Correspondence: Joerg Heineke, European Center for Angioscience (ECAS), Department of Cardiovascular Physiology, Medical Faculty Mannheim, Heidelberg University, Ludolf-Krehl-Straße 7 – 11, 68167 Mannheim, Germany.
E-mail: joerg.heineke@medma.uni-heidelberg.de

© 2024 The Author(s).
Published by Elsevier Inc. on behalf of The American Society of Gene and Cell Therapy.
This is an open access article under the CC BY license (http://creativecommons.org/licenses/by/4.0/).

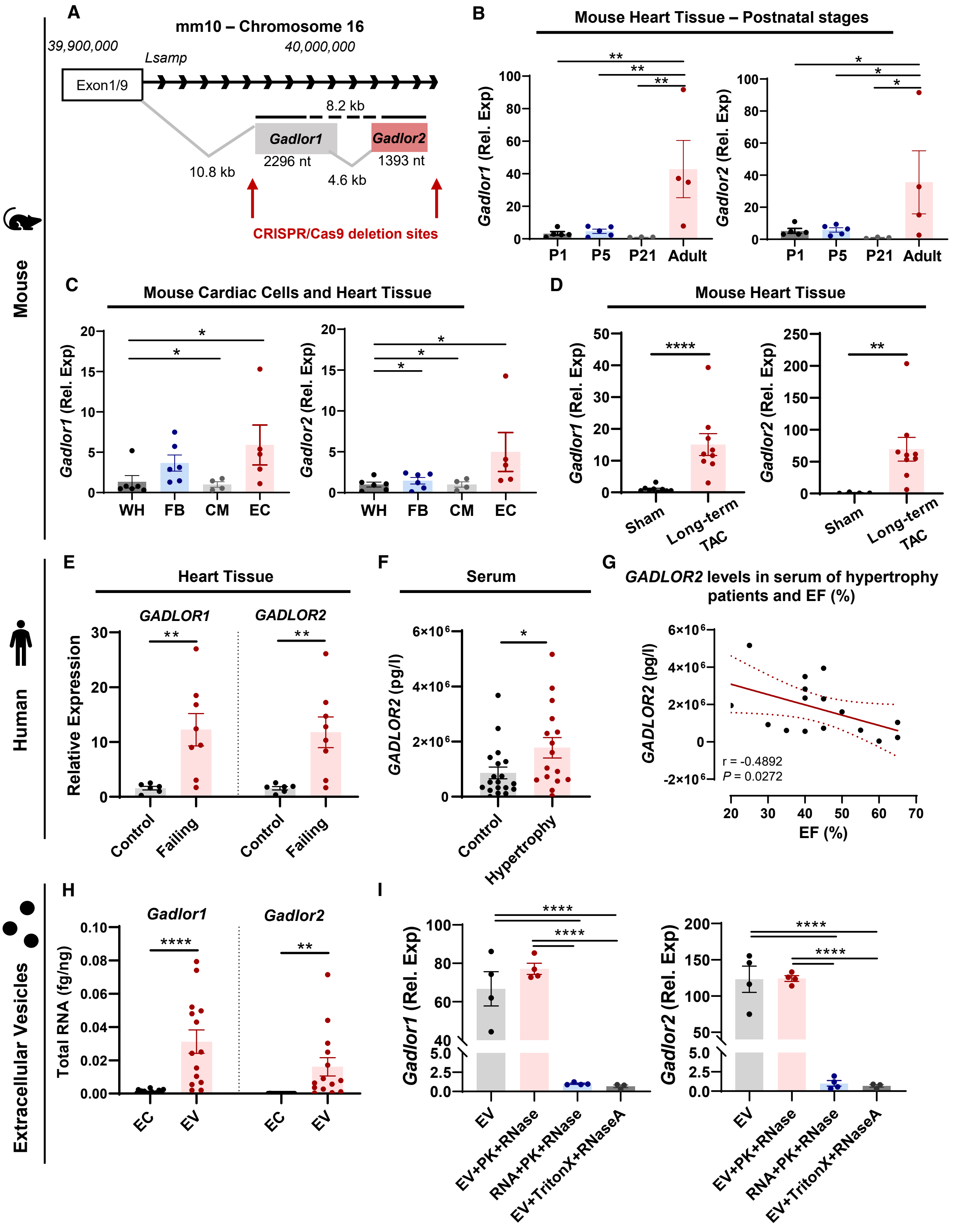

(legend on next page)

Recent studies have identified several lncRNAs with crucial roles in cardiac remodeling and heart failure.[11,13–16] Since non-coding transcripts are detectable in human body fluids such as in serum, circulating microRNAs (miRNAs) and lncRNAs are currently being investigated as clinical biomarkers, as well as for being paracrine mediators of cellular communication during stress conditions.[17–19] Intra-myocardial communication is a highly regulated process governing the complex physiology of the heart, whereby proper synchronization is required, for example, between endothelial cells (ECs) and CMs, which rely on EC-lined capillaries to provide nutrients and oxygen, but also paracrine factors regulating CM growth and function.[20–22] Cellular communication during pathological remodeling can be mediated by proteins, but also by the secretion and transfer of nanoscale extracellular vesicles (microvesicles and exosomes, termed small extracellular vesicles, EVs, here) that carry non-coding RNAs and alter the behavior of recipient cells.[23–30] It is currently emerging that EVs transport lncRNAs between cells and thereby affect EC function, cardiac fibrosis, and hypertrophy, but more work is needed.[26,28,31]

Here, we studied two related and secreted lncRNAs *Gadlor1* and *Gadlor2* (GATA-downregulated lncRNA), which are jointly upregulated after endothelial deletion of GATA2.[32] We found that the expression of both *Gadlor* lncRNAs are significantly increased in hearts of mice after transverse aortic constriction (TAC), but also in the myocardium and serum of patients suffering from chronic heart failure. Moreover, our results revealed that *Gadlor1* and *Gadlor2* are secreted within EVs predominantly by cardiac ECs, from which they are mainly taken up by CMs, although they also have autocrine/intracrine effects on ECs. In CMs, *Gadlor1/2* impact calcium handling, hypertrophy, and gene expression, while triggering anti-angiogenic effects in ECs. Our study reveals that secreted *Gadlor* lncRNAs aggravate cardiac remodeling but might protect from arrhythmias by enhancing calcium cycling.

RESULTS

Increased cardiac expression of *Gadlor* lncRNAs during pressure overload in mice and in human failing hearts

Earlier observations by our group had identified two previously unknown lncRNAs *Gadlor1* and *Gadlor2*, which were markedly and jointly upregulated in cardiac ECs of endothelial-specific GATA2 knockout (KO) mice.[32] *Gadlor1* (AK037972) and *Gadlor2* (AK038629) are located in close proximity to each other on mouse chromosome 16, and are embedded after exon1 of the *Lsamp* (limbic system-associated membrane protein) gene in its intronic region (Figures 1A and S1A–S1C). *Lsamp* is mainly expressed in the brain, and we did not detect significant *Lsamp* expression in the heart or cardiac ECs, and *Lsamp* expression patterns did not change in response to *Gadlor* KO in multiple organs (Figures S1D–S1F). *Gadlor1* and *Gadlor2* are not matched with any known mouse peptides (BlastP, the National Center for Biotechnology Information [NCBI] database), and we verified that they are non-coding transcripts by using the CPAT algorithm to assess protein-coding probability (Figure S1G). Cardiac *Gadlor1* and *2* expression is low in the direct postnatal period, but both transcripts become upregulated in parallel in the adult (2 month old) heart (Figure 1B). Cellular expression analysis among the main cell types in the heart revealed the highest *Gadlor1* and *Gadlor2* expression in ECs, followed by fibroblasts (FBs), while the lowest levels were detected in CMs (Figure 1C). In these analyses, *Gadlor1* and *2* lncRNA levels were also significantly enriched in ECs compared with whole heart tissue.

Next, we assessed *Gadlor1/2* RNA levels in cardiac disease. Myocardial *Gadlor1* and *2* expression was markedly and jointly upregulated in the chronic phase (12 weeks) after TAC versus sham (Figure 1D). Predominant endothelial expression and the increased abundance during pressure overload were confirmed by fluorescent *in situ* hybridization and RT-qPCR (Figures S1H and S1I).

The conservation of *Gadlor1/2* RNA between human, mouse, and rat was analyzed with the EMBOSS-Water platform (Figure S2). Although lncRNAs generally show lower levels of sequence conservation, our analysis revealed notable conservation, particularly higher for *Gadlor2* (approximately 72% and 87% for *Gadlor2* and 47% and 79% for *Gadlor1* in human and rat genome when aligned to the mouse genome). Importantly, *GADLOR1/2* expression was strongly induced in the myocardium of patients suffering from advanced heart failure (Figure 1E). We established an assay to specifically detect and quantify *GADLOR2* concentrations in human serum, which were significantly elevated in patients with heart failure due to aortic stenosis (Figure 1F). In this patient cohort, high *GADLOR2* serum concentrations were found mainly in patients with low left ventricular

Figure 1. *Gadlor* lncRNAs are primarily expressed in cardiac endothelial cells (ECs) and their expression is significantly upregulated in mice following pressure-overload and in human failing hearts

(A) Representative scheme of AK037972 (*Gadlor1*) and AK038629 (*Gadlor2*) locus on mouse chromosome 16 and positions of the CRISPR-Cas9 deletion sites used to generate *Gadlor*-KO mouse line. (B) *Gadlor1* and *Gadlor2* expression levels in mouse cardiac tissue from different post-natal developmental stages (P1, $n = 5$; P5, $n = 5$; P21, $n = 3$; and adult [9 weeks old], $n = 4$), and in (C) different cardiac cell types (ECs, $n = 5$; FBs, $n = 6$; CMs, $n = 4$) and heart tissue (WH, $n = 6$). (D) *Gadlor* lncRNA levels in mouse cardiac tissue after long-term (12 weeks, $n = 9$) TAC (transverse aortic constriction) compared with sham. (E) *GADLOR1* and *2* expressions in control (healthy, $n = 6$) and human failing heart tissue ($n = 8$) samples that were obtained from aortic stenosis patients. (F) Detection of *GADLOR2* levels (pg/L) in human serum of healthy volunteers ($n = 19$) and patients with aortic stenosis ($n = 16$). (G) Correlation analysis of *GADLOR2* levels (pg/L) with ejection fraction (%) of hypertrophy patients ($n = 16$) (Pearson correlation, Pearson r = −4,892 and p value = 0.0272). (H) Quantification of RNA of *Gadlor1* and *Gadlor2* in ECs and EC-derived EVs (fg *Gadlor*/ng total RNA). (I) Detection of *Gadlor* lncRNAs in EC-derived EVs with proteinase K (PK), RNase, or Triton X-100 as indicated. Data are shown as mean ± SEM. Data normality was evaluated with the Shapiro-Wilk test. p values were calculated with Student's t test for parametric (or Mann-Whitney for non-parametric) for comparing two groups, and one-way ANOVA was applied for comparing multiple groups followed by Fisher's LSD post-hoc test. $^*p < 0.05$, $^{**}p < 0.01$, $^{***}p < 0.001$, $^{****}p < 0.0001$.

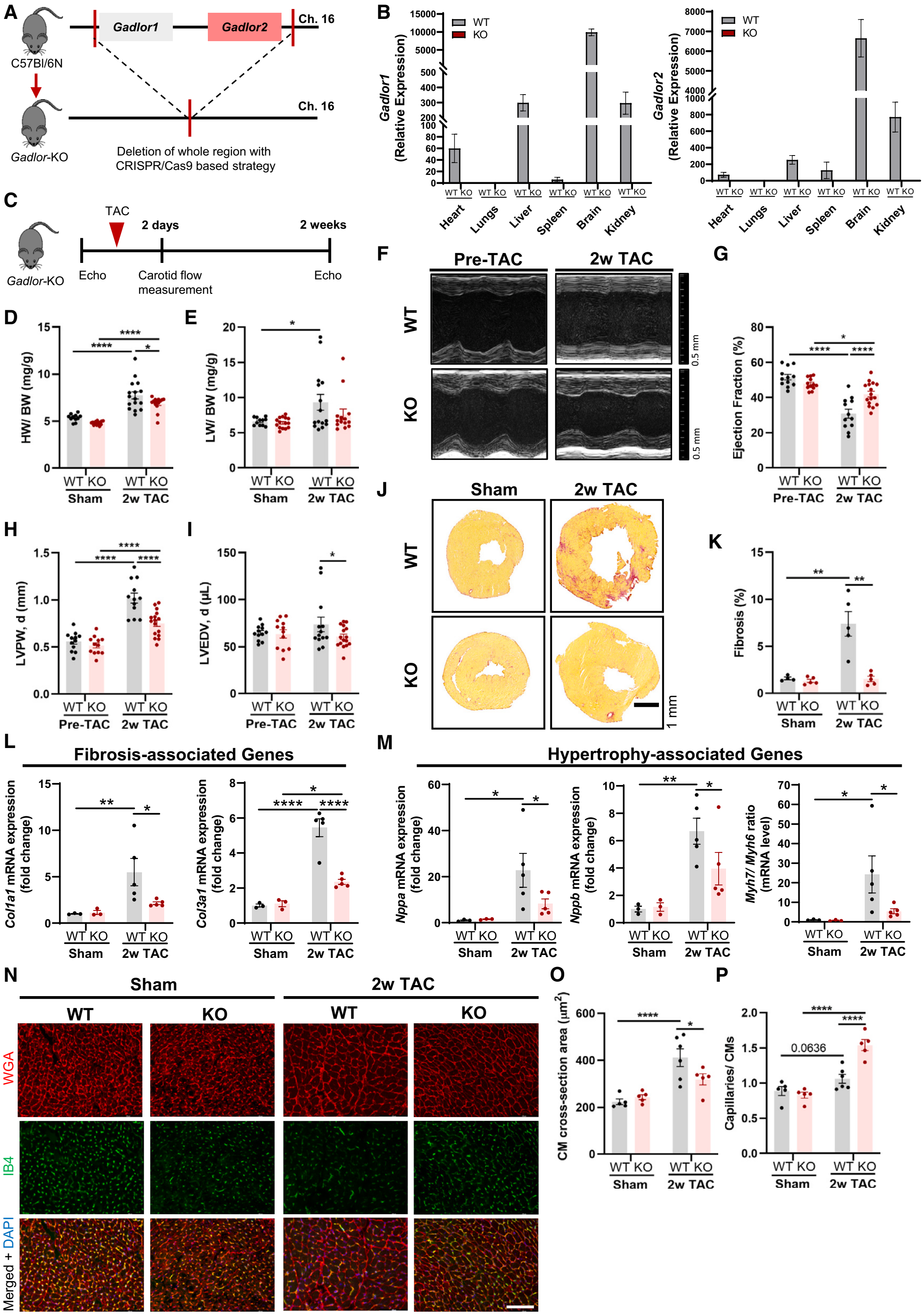

(legend on next page)

ejection fraction, and a negative correlation between these two parameters was identified (Figure 1G). We were not able to establish a similar assay to quantify *GADLOR1* in human serum. Taken together, our results demonstrate *Gadlor1* and *Gadlor2* as lncRNAs that are co-upregulated in diseased mouse and human hearts.

Gadlor lncRNAs are enriched in EC-derived EVs

Since *GADLOR2* was detectable in the serum of heart failure patients, and secreted EVs can contain lncRNAs,[26,29] we hypothesized that *Gadlor1/2* might be secreted within EVs. To test our hypothesis, the abundance of *Gadlor1/2* was analyzed in cultured primary mouse cardiac ECs and EVs collected and purified from the supernatant of the ECs by ultracentrifugation (Figure S3A). Initially, we characterized the isolated EVs with electron microscopy (EM), nanoparticle tracking analysis (NTA), and FACS. We found typical microvesicles and exosomes in EM, and the average size of the particles was slightly larger than 100 nm in diameter by NTA, which showed that they were in the range of microvesicles and exosomes (together called EVs in this study) (Figures S3B and S3C). Flow cytometry assessment also confirmed the presence of CD63, CD9, and CD54 as common cell surface markers for EVs (Figure S3D).

Interestingly, concentration of *Gadlor1/2* was markedly higher in cardiac EC-derived EVs compared with cardiac ECs themselves (Figure 1H). EV *Gadlor1* and *2* RNA levels were not affected by proteinase K (PK), or by RNase A, which indicated that *Gadlor1/2* were encapsulated within vesicles and protected from enzyme degradation (Figure 1I). Indeed, *Gadlor* lncRNAs were not protected upon direct incubation with PK and RNase A, or when EVs were dissolved by Triton X-100, before RNase A was applied (Figure 1I). In addition to primary cardiac ECs, also C166 mouse ECs secreted *Gadlor* RNAs in EVs (Figure S3E). Altogether, these results suggest that *Gadlor1* and *Gadlor2* are highly enriched and encapsulated in EC-derived EVs.

Gadlor-KO mice are protected from cardiac hypertrophy and dysfunction during pressure overload

To investigate the role of *Gadlor* lncRNAs, systemic *Gadlor* KO mice were generated by deletion of the respective region of mouse chromosome 16 (including *Gadlor1* and *2*, since both were consistently jointly regulated in our analyses) by using a CRISPR-Cas9-based strategy (Figures 1A and 2A). Unchallenged *Gadlor*-KO mice were indistinguishable from their wild-type (WT) littermates based on appearance, body weight (BW), heart weight (HW), and baseline echocardiographic analysis (Figures S4A–S4D). We detected, however, a mild increase in liver and a decrease in brain weight in KO animals compared with WT littermates (Figure S4E).

The ablation of *Gadlor1/2* was confirmed in KO animals by RT-qPCR in different organs (Figure 2B). We found *Gadlor1* and *Gadlor2* expression in heart, liver, brain, spleen, and kidney, with the highest expression in the brain of WT mice (Figure 2B). To study the effect of *Gadlor1* and *Gadlor2* in cardiac remodeling during pressure overload, WT and *Gadlor*-KO animals were subjected to TAC or sham surgery and subsequently monitored for 2 weeks (Figure 2C). The carotid flow was measured after 2 days of TAC in the right and left common carotid arteries (RCCA and LCCA, respectively) to evaluate the strength of the aortic constriction. The RCCA/LCCA flow ratio increased to a similar degree in both WT and *Gadlor*-KO animals, indicating comparable pressure overload (Figures S4F and S4G). We found a significant increase in HW/BW ratio after 2 weeks of TAC in both WT and *Gadlor*-KO compared with sham animals; however, less hypertrophy (i.e., a reduced HW/BW ratio) was observed in *Gadlor*-KO mice (Figure 2D). In addition, heart failure-induced pulmonary congestion was only observed in WT animals after TAC, whereas *Gadlor*-KO animals were protected (Figure 2E). In echocardiography, *Gadlor*-KO mice showed less cardiac systolic dysfunction, a markedly reduced left posterior ventricular wall thickness and less chamber dilation (measured as left end-diastolic volume) compared with WT mice after TAC (Figures 2F–2I).

Deletion of *Gadlor1* and *Gadlor2* alleviates CM hypertrophy and fibrosis *in vivo*

Sirius red staining revealed strongly diminished myocardial fibrosis in *Gadlor*-KO mice, which was supported by reduced cardiac *Col1a1* and *Col3a1* mRNA levels after TAC (Figures 2J–2L). A lower expression of *Nppa*, *Nppb*, a lower *Myh7/Myh6* RNA ratio, and a reduced CM cross-sectional area confirmed blunted cardiac hypertrophy in *Gadlor*-KO animals compared with WT littermates after TAC (Figures 2M–2O). In addition, Isolectin B4 staining of capillaries

Figure 2. Deletion of *Gadlor* lncRNAs reduces cardiac hypertrophy and fibrosis during cardiac pressure overload

(A) Scheme of *Gadlor*-KO mouse line generation by systemic deletion of the region on chromosome 16 containing both *Gadlor1* and *Gadlor2* by a CRISPR-Cas9 approach. (B) Validation of *Gadlor*-KO mouse line by assessing the relative expression of *Gadlor1/2* in the indicated organs in adult WT and *Gadlor*-KO mice ($n \geq 8$). The difference in *Gadlor1/2* expression levels between WT and *Gadlor*-KO animals were significant for all organs in which *Gadlor1* and *Gadlor2* were detected. Heart, *$p < 0.05$; liver and kidney, ***$p < 0.001$; brain, ****$p < 0.0001$). (C) Experimental design of TAC operation for *Gadlor*-KO and WT littermates. (D) Heart weight (mg) to body weight (g) ratio (HW/BW) and (E) lung weight (mg) to body weight (g) ratio of *Gadlor*-KO and WT littermates in sham and after 2 weeks of TAC ($n \geq 11$). (F) Representative echocardiography images of *Gadlor*-KO and WT mice before (pre-TAC) and after 2 weeks of TAC (2w TAC) shown in parasternal long axis mode, and analysis of (G) left ventricle (LV) ejection fraction (EF%), (H) LV posterior wall thickness in diastole (mm) and (I) LV end diastolic volume (μL), ($n \geq 12$). (J) Representative Sirius red fibrosis staining in cardiac tissue sections of sham and 2w TAC animals. Scale bar, 1 mm. (K) Quantification of fibrotic area ($n \geq 4$). (L) Relative expression of fibrosis marker genes *Col1a1* and *Col3a1* and (M) hypertrophy marker genes *Nppa*, *Nppb*, and *Mhy7/6* ratio in mRNA levels in heart tissue samples of sham ($n = 3$) and 2w TAC ($n = 5$). (N) Representative immunofluorescence images of cardiac tissue samples stained with WGA (wheat-germ agglutinin coupled with Alexa Fluor 555) and IB4 (Isolectin B4 coupled with Alexa Fluor 488) in sham and 2week TAC samples from *Gadlor*-KO and WT littermates. Scale bar, 100 μm. Quantification of (O) cardiomyocyte cross-sectional area and (P) myocardial capillary density per cardiomyocyte ratio. Data are shown as mean ± SEM. Data normality was evaluated with Shapiro-Wilk test and *p* values were calculated with Student's t test for comparing two groups and two-way ANOVA for grouped analysis followed by Fisher's LSD post-hoc test. *$p < 0.05$, **$p < 0.01$, ***$p < 0.001$, ****$p < 0.0001$.

revealed a substantial increase of capillary density in the myocardium of *Gadlor*-KO mice compared with WT animals after TAC (Figures 2N and 2P). Together, our data showed that deletion of *Gadlor1/2* protected against cardiac hypertrophy, systolic dysfunction, capillary rarefaction, and myocardial fibrosis during pressure overload.

Overexpression of *Gadlor1* and *Gadlor2* via EVs triggers cardiac dysfunction and fibrosis

To decipher the functional effect of increased *Gadlor1* and *Gadlor2* levels during pressure overload, we administered control EVs and *Gadlor*-enriched EVs to mouse hearts directly before TAC surgery by intraventricular injection (and concomitant cross-clamping of the aorta and pulmonary artery distal of the coronary vessels origin, to allow perfusion of the injected EVs through the coronaries). Control EVs were produced by purifying EC-derived EVs from the supernatant of control adenovirus (Ad.βgal)-infected C166 mouse ECs, and *Gadlor1*- and *Gadlor2*-containing EVs were purified from the supernatant of Ad.*Gadlor1*- and Ad.*Gadlor2*-infected C166 ECs (Figure 3A). We detected significantly higher levels of myocardial *Gadlor1* and *Gadlor2* after 1 week of EV administration (Figure 3B). *Gadlor*-EV-treated mice exerted a marked reduction of cardiac systolic function after 1 and 2 weeks of TAC, as well as increased wall thickness compared with control EV-treated animals (two cohorts were examined, cohorts I and II, Figures 3A–3E). Histological analyses by Sirius red staining showed exaggerated myocardial fibrosis in *Gadlor*-EV-treated mice, which was confirmed by qPCR of *Col1a1* and *Col3a1* mRNA expression in the myocardium (Figures 3F and 3G). Furthermore, increased expression of *Nppa* and *Nppb*, as well as a trend toward larger CM cross-sectional area verified increased hypertrophic remodeling in *Gadlor*-EV-treated mice (Figures 3H–3J). The myocardial capillary density was not changed between experimental groups (Figure 3K). We concluded that overexpression of *Gadlor1* and *Gadlor2* via administration of endothelial-derived EVs aggravated cardiac dysfunction and hypertrophy, and triggered fibrosis during pressure overload.

Gadlor-KO mice exert increased mortality during chronic pressure overload

Next, we studied whether the protective effects of *Gadlor1/2* ablation were maintained during persisting long-term pressure overload (Figure 4A). Similar to our findings after 2 weeks of TAC, *Gadlor*-KO animals had a significantly reduced HW/BW ratio after 8 weeks of TAC compared with WT littermates, an improved systolic left ventricular function with reduced posterior wall thickness, an increased capillary density, diminished CM hypertrophy, and markedly less fibrosis (Figures 4B–4J). Despite retaining better systolic function and improved remodeling features, we observed a considerably higher mortality rate in *Gadlor*-KO mice starting between 2 and 3 weeks after TAC (Figure 4K). Interestingly, WT and KO animals were indistinguishable in terms of appearance, behavior, and mobility after TAC operation, and death in *Gadlor*-KO mice was unexpected and sudden in nature, possibly due to cardiac arrhythmia.

Gadlor1 and *Gadlor2* affect gene expression and angiogenic function in ECs

Since ECs are the main cardiac cell type expressing *Gadlor1/2*, and since we observed an increased capillary density in the myocardium of *Gadlor*-KO mice after 2 and 8 weeks of TAC (see above), we performed RNA sequencing (RNA-seq) from isolated WT and *Gadlor*-KO ECs after 2 weeks of TAC. The purity of ECs in our MACS-based isolation procedure is >90%.[33] The genome-wide transcriptome analysis via bulk RNA-seq identified a total of 3,355 differentially expressed (DE) genes between WT and *Gadlor*-KO, which are shown in the heatmap in Figure 5A. Gene ontology analysis from DE genes and RT-qPCR revealed and confirmed upregulation of angiogenesis, mitotic cell cycle and respiratory electron transport, and downregulation of inflammatory response genes in *Gadlor*-KO EC after 2 weeks of TAC (Figures 5B–5D). In addition, myocardial staining for Ki67 as a proliferation marker revealed more Ki67-positive ECs in *Gadlor*-KO mice after TAC (Figures 5E and 5F). In turn, overexpression of *Gadlor1* and *Gadlor2* by adenovirus in C166 mouse ECs blunted the increase in EC sprouting activity during FGF2 stimulation and reduced sprouting further in the presence of TGF-β in comparison with Ad.βgal (control) infected cells (Figures 5G and 5H). Furthermore, heart ECs isolated from *Gadlor*-KO mice exhibited a higher proliferation rate compared with WT ECs. Reintroduction of *Gadlor1/2* lncRNAs into these *Gadlor*-KO heart ECs using adenovirus attenuated the pro-angiogenic phenotype observed (Figure 5I). In summary, *Gadlor* lncRNAs exert anti-angiogenic properties in ECs.

Cardiac FBs of *Gadlor*-KO mice induce less fibrosis-associated genes after TAC

Based on our findings, FBs exerted the second highest *Gadlor* lncRNAs expression after ECs in the myocardium (Figure 1C). To study the reason for decreased fibrosis in hearts upon *Gadlor*-KO after TAC, we performed bulk RNA-seq from isolated cardiac FBs after 2 weeks of TAC. The purity of cardiac FBs in our MACS-based isolation procedure is >90%.[33] Genome-wide analysis revealed a total of 915 downregulated and 998 upregulated genes in *Gadlor*-KO FBs compared with WT mice after TAC, which are shown in a heatmap in Figure S5A. Among these, we detected a strong downregulation of mainly ECM organization and growth factor associated genes (confirmed by RT-qPCR: *Fgfr1*, *Igf1*, *Col1a1*, *Col3a1*, *Col6a1*) (Figures S5B–S5D). In addition, genes related to the regulation of angiogenesis, blood vessel, and endothelium development were upregulated in *Gadlor*-KO FBs, which suggested a contribution of FBs to the enhanced angiogenesis observed in *Gadlor*-KO mice upon overload. The increased expression of *Angpt2*, *Efna1*, *Aurkb*, and *Dll1* in cardiac FBs from *Gadlor*-KO mice was confirmed by RT-qPCR (Figure S5C). Upon adenoviral overexpression of *Gadlor1* and *Gadlor2* followed by phenylephrine (PE) stimulation, neonatal rat cardiac FBs (NRFBs), in turn, exerted a decreased expression of *Angpt2*, and an increased expression of *Col1a1* and *Col3a1*, but no differential expression was observed for *Col4a1* or *Col6a1* (Figure S5E). We concluded that *Gadlor* lncRNAs trigger pro-fibrotic gene expression in cardiac FBs.

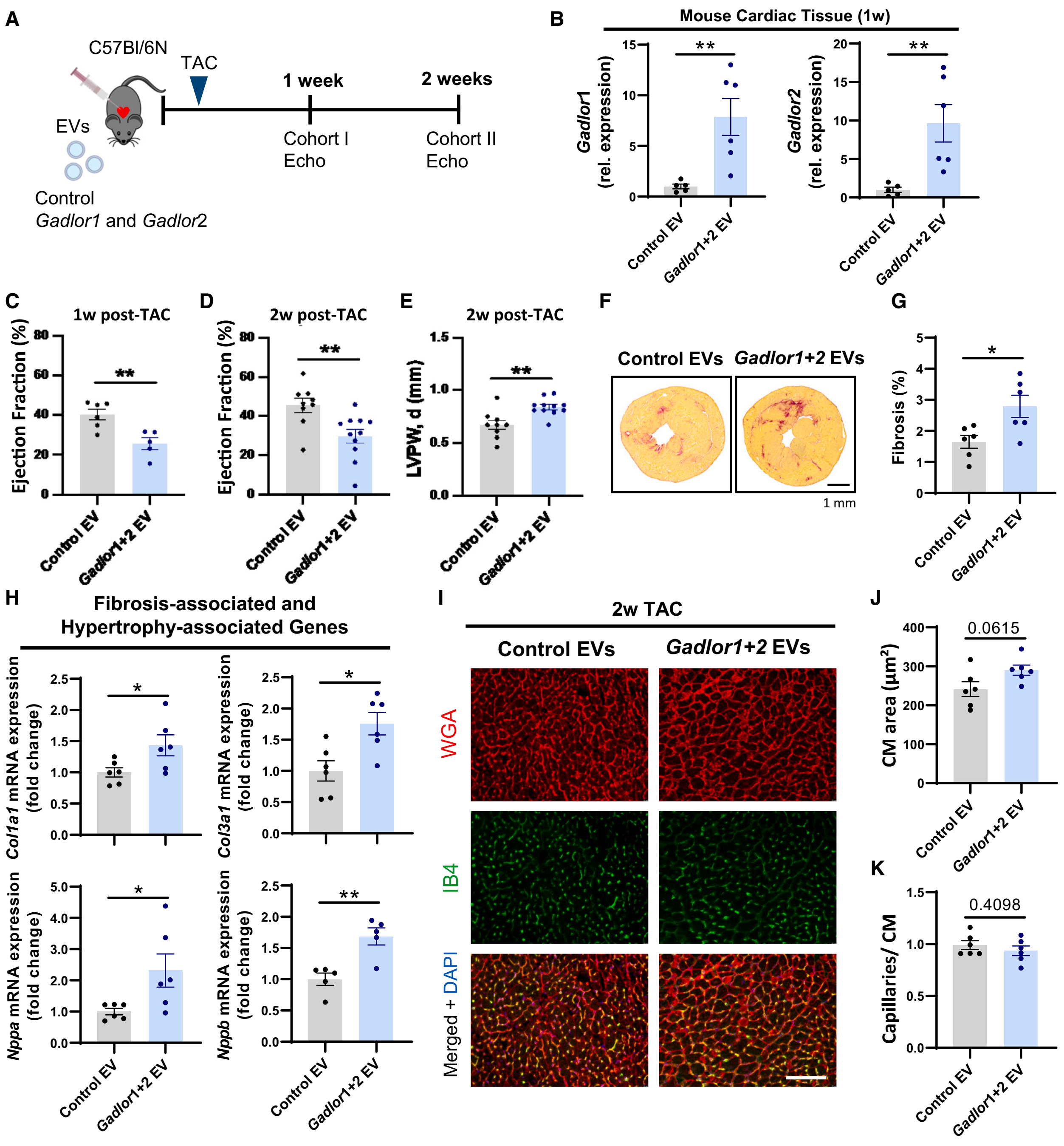

Figure 3. Overexpression of *Gadlor1* and *Gadlor2* via extracellular vesicles (EVs) triggers cardiac dysfunction and development of fibrosis after TAC

(A) Scheme of the experimental design depicting the injection of *Gadlor1* and *Gadlor2* containing extracellular vesicles for overexpression followed by TAC operation. (B) Relative expression of *Gadlor1 and Gadlor2* in cardiac tissue samples after 1 week TAC (indicated as cohort I) to validate the overexpression after injection of control (Ad.βgal-treated samples, $n = 5$) and *Gadlor*-containing (Ad.*Gadlor1*- and Ad.*Gadlor2*-treated samples, $n = 6$) EVs. Echocardiographic analysis of (C) left ventricle (LV) ejection fraction (EF%) after 1 week TAC, and (D) LV-EF%, (E) LV posterior wall thickness in diastole (mm) after 2 weeks of TAC in mice injected with control EVs and *Gadlor1* and *Gadlor2* EVs. (F) Representative images of Sirius red staining. Scale bar, 1 mm. (G) Quantification of fibrotic area after 2 weeks of TAC in mice injected with control EVs and *Gadlor1* and *2* EVs. (H) Relative expression of fibrosis marker genes *Col1a1* and *Col3a1* and hypertrophy marker genes *Nppa* and *Nppb* in heart tissue samples of control EV-treated ($n = 6$) and *Gadlor* EV-treated ($n = 6$) mice. (I) Representative immunofluorescence images of cardiac tissue samples stained with WGA (wheat-germ agglutinin coupled with Alexa Fluor 555) and IB4 (Isolectin B4 coupled with Alexa Fluor 488) in samples from control-EV- and *Gadlor*-EV-treated animals. Scale bar, 100 μm. Quantification of (J) cardiomyocyte cross-sectional area and (K) myocardial capillarization after EV treatment followed by 2 weeks of TAC. Data are shown as mean ± SEM. Data normality was evaluated with Shapiro-Wilk test and *p* values were calculated with Student's t test. *$p < 0.05$, **$p < 0.01$, ***$p < 0.001$, ****$p < 0.0001$.

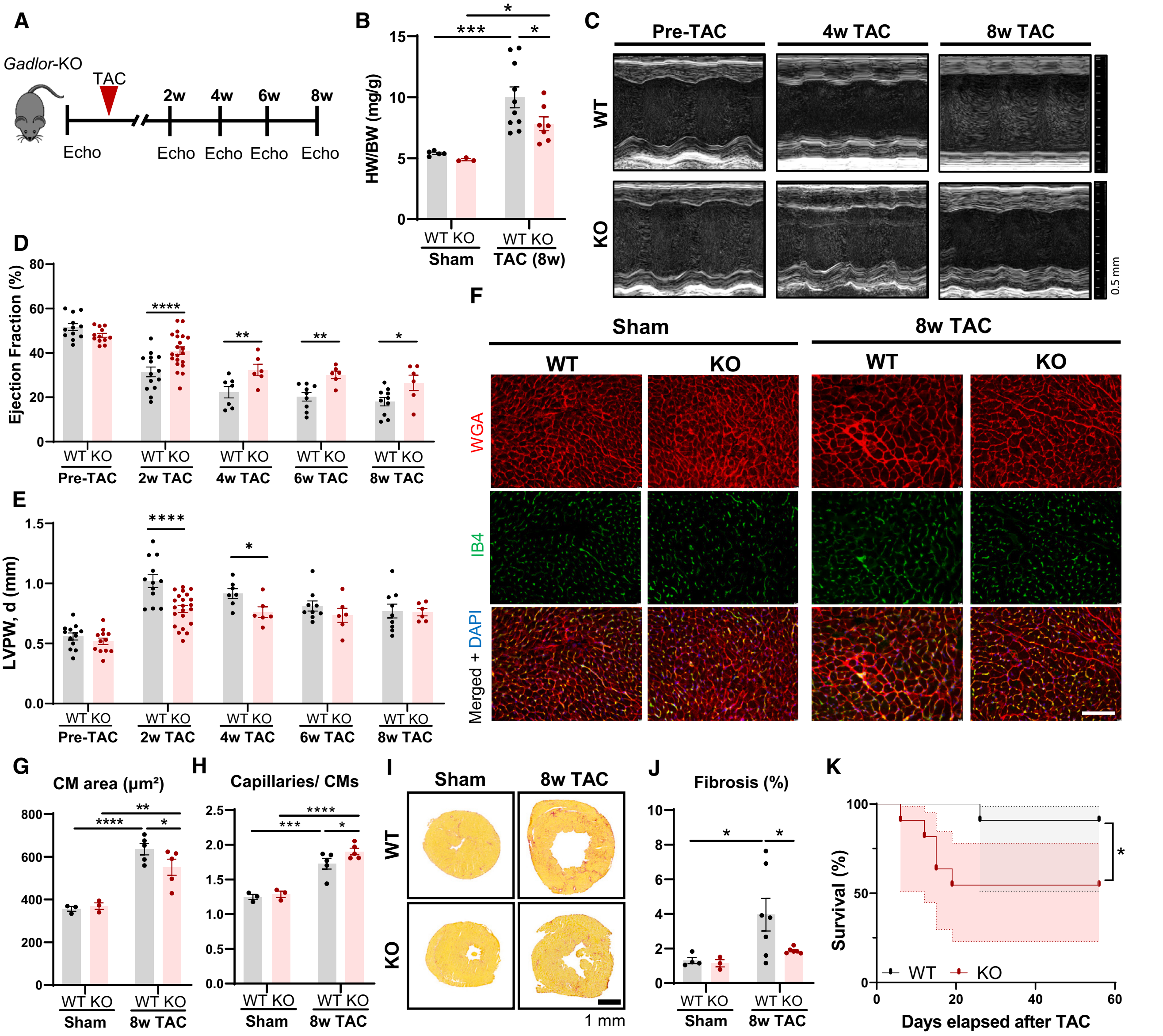

Figure 4. *Gadlor*-KO mice showed higher mortality despite retaining better heart function and structural remodeling after persistent pressure overload
(A) Experimental design of long-term TAC operation for *Gadlor*-KO and WT littermates up to 8 weeks after TAC. (B) Heart weight (mg) to body weight (g) ratio (HW/BW) of *Gadlor*-KO and WT mice in age comparable sham ($n \geq 3$) and after 8 weeks TAC ($n \geq 7$). (C) Representative echocardiography images of *Gadlor*-KO and WT mice before (pre-TAC) and after 4 and 8 weeks TAC shown in parasternal long axis mode, and analysis of (D) left ventricle (LV) ejection fraction (EF%) and (E) left ventricle (LV) posterior wall thickness in diastole (mm), (unpaired analysis). (F) Representative immunofluorescence images of cardiac tissue samples stained with WGA (wheat-germ agglutinin coupled with Alexa Fluor 555) and IB4 (Isolectin B4 coupled with Alexa Fluor 488) in sham ($n = 3$) and 8-week TAC ($n = 5$) samples from *Gadlor*-KO and WT littermates. Scale bar, 100 μm. Quantification of (G) cardiomyocyte cross-sectional area and (H) myocardial capillary density per cardiomyocyte ratio. (I) Representative Sirius red fibrosis staining in cardiac tissue sections of sham ($n \geq 3$) and 8 week TAC ($n \geq 6$) mice. Scale bar, 1 mm. (J) Quantification of fibrotic area (%). (K) Survival of *Gadlor*-KO and WT mice after TAC ($n = 11$). Probability of survival compared with log rank (Mantel-Cox) test. Data are shown as mean ± SEM. Data normality was evaluated with Shapiro-Wilk test and p values were calculated with Student's t test for comparing two groups and two-way ANOVA for grouped analysis followed by Fisher's LSD post-hoc test. *$p < 0.05$, **$p < 0.01$, ***$p < 0.001$, ****$p < 0.0001$.

Gadlor lncRNAs are transferred to CMs via EC-derived EVs

Since *Gadlor1* and *Gadlor2* were mainly secreted by ECs, we reasoned that these lncRNAs might exert paracrine effects due to EV-mediated transfer into CMs, where they could affect contraction, arrhythmia, or hypertrophy. To analyze whether *Gadlor1/2* are transferred from ECs to CMs, we initially overexpressed *Gadlor1/2* by adenovirus in C166

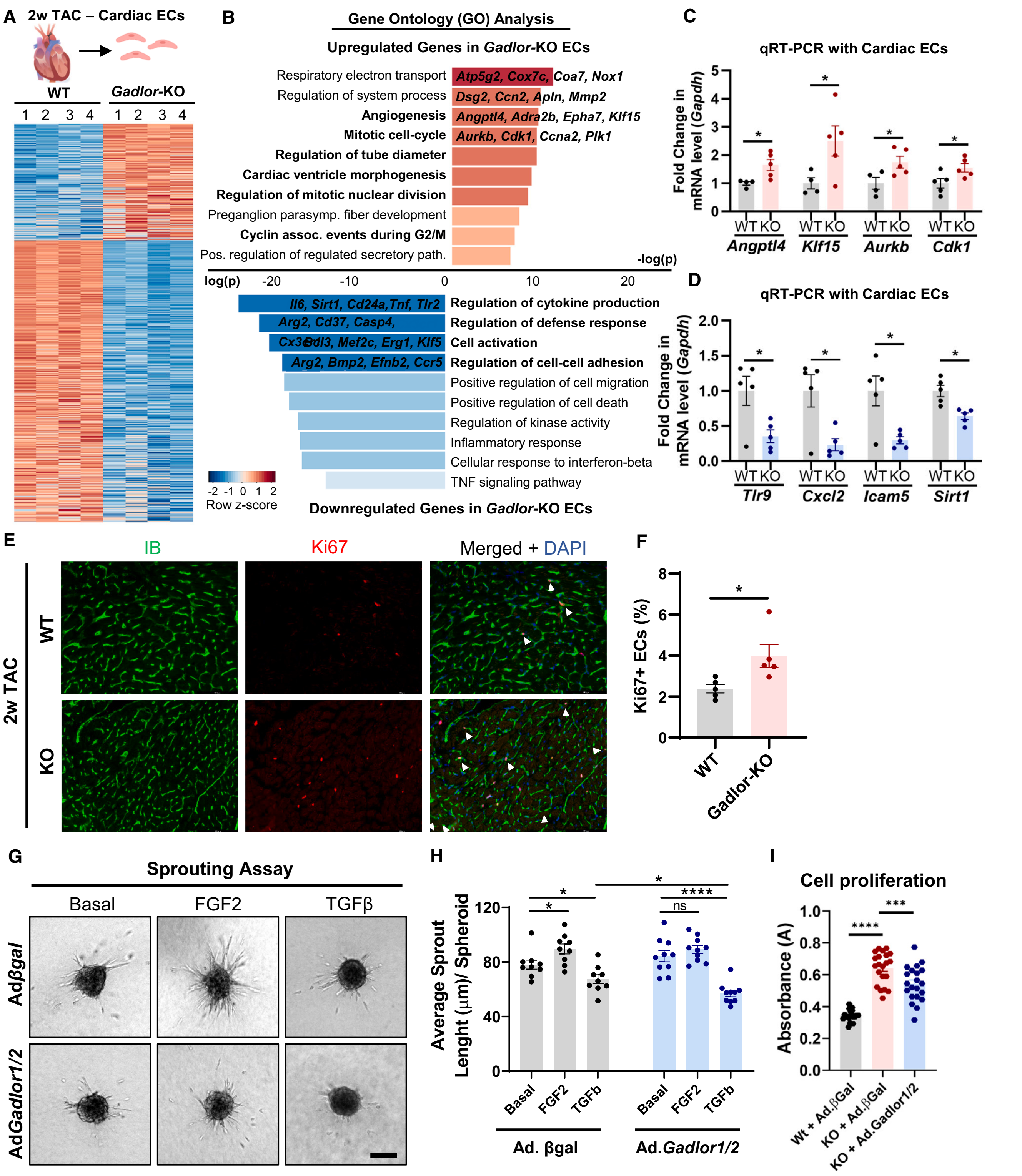

Figure 5. Deletion of *Gadlor* lncRNAs induces angiogenesis and expression of cell-cycle genes in cardiac ECs during pressure-overload

(A) Heatmap showing differentially regulated genes revealed by bulk RNA-seq of cardiac endothelial-cells after 2-week TAC. (B) Bar plots showing the gene ontology (GO) analysis of upregulated and downregulated genes in *Gadlor*-KO ECs compared with WT ECs after 2 weeks TAC (red, upregulated in *Gadlor*-KO; blue, downregulated in

(legend continued on next page)

mouse ECs, and purified EVs from the supernatant of these cells (Figure 6A). We confirmed the enhancement of *Gadlor1/2* expression in EVs upon *Gadlor1/2* overexpression versus control EVs (Figure 6B). The collected EVs were transferred either to neonatal rat cardiomyocytes (NRCMs) or NRFBs. RT-qPCR analysis showed a significantly higher abundance of *Gadlor1/2* in NRCMs incubated with *Gadlor*-EVs compared with samples incubated with control EVs or CMs without any EV treatment, which indicated that EVs were taken up by CMs (Figure 6C). *Gadlor* lncRNAs were also transferred from ECs to CMs by control EVs, since higher CM *Gadlor1/2* levels were observed after control EV compared with non-EV treatment. In addition, a mild increase in both *Gadlor1* and *Gadlor2* abundance was also observed in NRFBs after incubation with *Gadlor* EVs; however, at a much lower degree compared with NRCMs (Figure 6C). We visualized the uptake of the fluorescently labeled (PKH67) EVs into recipient CMs, which was analyzed with confocal microscopy after 6 and 24 h (Figure 6D). 3D imaging by confocal z stacks confirmed that EVs were internalized into the recipient CMs rather than being attached to the surface (Video S1).

Furthermore, we performed co-culture experiments with isolated *Gadlor*-KO CMs (lacking endogenous *Gadlor1/2* expression) and WT mouse cardiac endothelial cells (MCECs, on cell culture inserts, which allow the transfer of EVs, due to a pore size of 1 μm) for 48 h (Figure 6E). *Gadlor*-KO CMs were used to exclude confounding effects of endogenous *Gadlor1/2* expression with exogenous *Gadlor1/2* uptake from EC-derived EVs. As expected, we did not find any *Gadlor1/2* expression in isolated *Gadlor*-KO CMs when they were kept in monoculture, whereas we detected *Gadlor1* and *Gadlor2* after co-culture with WT MCECs (Figure 6F). This suggested that *Gadlor1/2* were transferred into *Gadlor*-KO CMs from WT MCECs, possibly via EV-mediated cell-to-cell transfer. MCECs that were co-cultured with *Gadlor*-KO CMs also exerted an increase of *Gadlor1/2* expression (Figure 6F), which might suggest a possible feedback mechanism between CMs and ECs. The addition of GW4869 as inhibitor of exosome (i.e., small EV) generation into the co-culture system ablated the transfer of EC-derived *Gadlor1/2* to KO CMs as well as the putative feedback mechanisms, suggesting that both might depend on small EVs (Figure 6G). Next, we wanted to assess whether WT CMs depend on ECs for *Gadlor1/2* expression. Indeed, inclusion of GW4869 into co-culture of WT CMs with WT MCECs, markedly reduced *Gadlor1/2* levels in the CMs. This indicated that ECs provide *Gadlor1/2* to CMs and might thereby relay paracrine signals to these cells (Figure 6H).

Subcellular fractionation studies to localize *Gadlor* lncRNAs following EV-mediated transfer showed that *Gadlor1* and *Gadlor2* were detected in the cytosol and in a bit higher level in the nucleus of recipient CMs (Figure 6I). Overall, endothelial-derived *Gadlor1/2*-containing EVs are mainly taken up by CMs, where they might act in the cytosol as well as in the nucleus. Cardiac FBs may mainly rely on their own cellular *Gadlor1/2* expression.

Gadlor lncRNAs bind to calcium/calmodulin-dependent protein kinase type II in CMs

LncRNAs can interact with proteins to affect their regulatory functions.[34] Even though CMs showed the least endogenous levels of *Gadlor* lncRNAs, we still observed strong effects in CMs (e.g., reduced hypertrophy and potentially arrhythmia) after *Gadlor1/2* deletion upon pressure overload. To decipher this further, we aimed at identifying the binding partners of *Gadlor1/2* in CMs by performing RNA antisense purification coupled with mass spectrometry (RAP-MS) in HL1 cardiac muscle cells after overexpression of *Gadlor1/2* (Figure 7A). Interestingly, we found calcium/calmodulin-dependent protein kinase type II, delta (CaMKIIδ) as the most significant binding partner of *Gadlor1/2* (Figure 7B). Next, we confirmed the interaction between CaMKII and *Gadlor1/2* with native RNA immunoprecipitation (RIP), i.e., immunoprecipitation of CaMKII, followed by RT-qPCR to detect *Gadlor1* and *Gadlor2* (Figure 7C). We also confirmed the specific interactions between CaMKII and *Gadlor1* and *Gadlor2* lncRNAs *in situ* by proximity ligation assay (Figure S6). We then investigated whether *Gadlor1/2* modulates CaMKII function by assessing phospholamban threonine-17 (Thr17) phosphorylation, which is mediated directly by CaMKII. Indeed, we found reduced and increased Thr17 phospholamban phosphorylation in *Gadlor*-KO mice or *Gadlor*-EV-treated mice after TAC, respectively (Figures 7D–7G). This indicated that *Gadlor1/2* might promote CaMKII activation in CMs.

Since *Gadlor* lncRNAs had influenced cardiac function, and CaMKII was identified as binding partner, which has a known role in CM excitation-contraction coupling, we investigated CM contractility and calcium dynamics with the CytoCypher High-Throughput (HT) system (Ionoptix) in isolated adult CMs after 1 week of TAC. Interestingly, during measurements, a considerably higher number of *Gadlor*-KO CMs with arrhythmic behavior was detected compared with WT CMs (Figure 7H). Sarcomere length traces (Figure 7I) indicated that *Gadlor*-KO CMs exhibited double contractions even though they were paced with electrical field stimulation, in which each stimulation usually results in a single contraction.

Gadlor-KO). Some exemplary genes were listed for selected GO terms. (C and D) Validation of selected genes from RNA-seq data with RT-qPCR in isolated cardiac ECs after 2 weeks of TAC (red, upregulated in *Gadlor*-KO; blue, downregulated in *Gadlor*-KO). (E) Representative immunofluorescence images of cardiac tissue samples stained with IB4 (Isolectin B4 coupled with Alexa Fluor 488) and Ki67 (coupled with Alexa Fluor 555) in 2 week TAC samples from *Gadlor*-KO and WT littermates (arrows showing Ki67+ ECs). Scale bar, 100 μm. (F) Quantification of Ki67-positive EC percentage (Ki67+ ECs%). (G) Representative images of sprouting assay with C166 mouse ECs after adenovirus treatment (Adβgal and Ad*Gadlor1/2*) followed by 24 h FGF2 or TGF-β treatment. Three-dimensional (3D) collagen matrix embedded spheroids were allowed to form sprouts for 24 h. Scale bar, 100 μm. (H) Quantification of average sprout length per spheroid. (I) Cell proliferation was detected by BrdU ELISA in isolated cardiac ECs from WT and *Gadlor*-KO with additional βgal or *Gadlor1/2* adenoviral treatment as indicated. Data are shown as mean ± SEM. Data normality was evaluated with Shapiro-Wilk test. p values were calculated with Student's t test for comparing two groups. For grouped analysis, p values were evaluated with two-way ANOVA followed by Fisher's LSD post-doc test. *$p < 0.05$, **$p < 0.01$, ***$p < 0.001$, ****$p < 0.0001$.

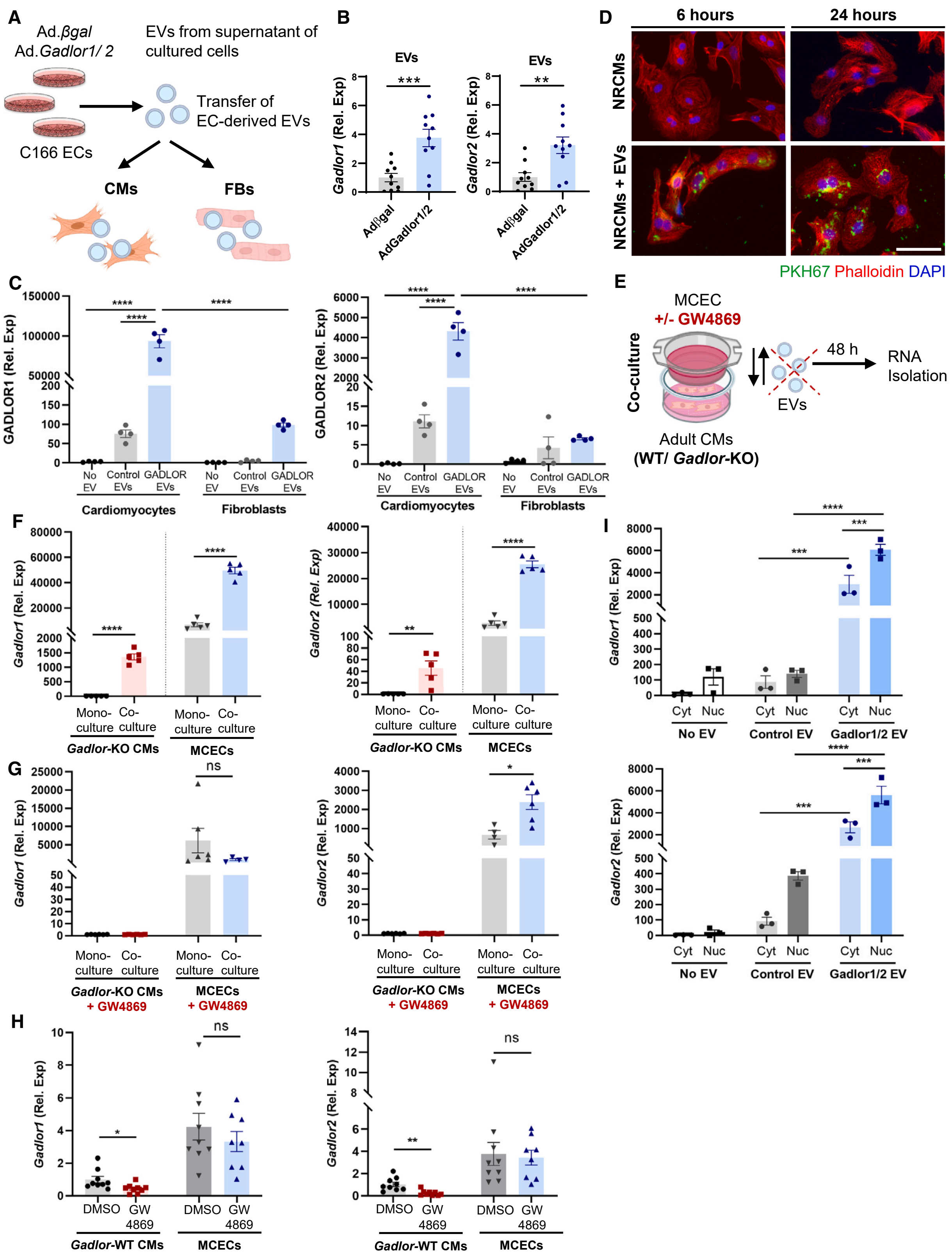

(legend on next page)

Interestingly, *Gadlor*-KO CMs displayed a much slower re-uptake of calcium into the sarcoplasmic reticulum (SR), which led to prolonged calcium transients. We also observed an increased calcium peak height in *Gadlor*-KO CMs (Figures 7J and 7K). In addition, while sarcomere shortening and its timing were not significantly affected, CM relaxation was delayed (Figure 7L). Treatment of adult mouse CMs with *Gadlor1/2*-enriched versus control EC-derived EVs, in turn, led to augmented sarcomere shortening and especially faster CM relaxation (Figure 7M). While calcium peak height was not changed, a faster diastolic calcium re-uptake was detected by a decreased tau value (Figure 7N). Collectively, *Gadlor*-KO myocytes displayed an enhanced propensity to arrhythmic beating, slower diastolic relaxation, and slower calcium re-uptake into the SR, which entailed increased intracellular calcium levels. Overexpression of *Gadlors1/2*, in turn, led to increased sarcomere shortening, faster CM relaxation, and faster re-uptake of calcium into the SR. These effects were partially inhibited upon KN93 (CaMKII inhibitor) treatment (Figure 7O), which decreased the sarcomere shortening and increased the time needed for re-uptake of calcium into the SR (tau) in *Gadlor*-EV-treated CMs, indicating that *Gadlor1/2* exert their effects on excitation-contraction coupling at least in part through CaMKII activation.

Gadlor1 and *Gadlor2* promote pro-hypertrophic and mitochondrial gene expression in CMs

Since *Gadlor1/2* were in part localized in CM nuclei after their transfer (Figure 6I), we hypothesized that they might also affect gene expression in these cells. Comprehensive transcriptome analysis after 2 weeks of TAC between isolated WT and *Gadlor*-KO CMs revealed that 2,492 genes were up-, while 2,956 were downregulated, after ablation of *Gadlor1/2* as shown in the heatmap in Figure 8A. Functional annotation of DE genes showed that genes related to vasculature development, ECM organization, and regulation of cytokine production were upregulated (Figure 8B), while mitochondrial organization, TCA cycle, and cardiac muscle contraction/hypertrophy related genes were downregulated in *Gadlor*-KO CMs (Figure 8B). We confirmed the upregulation of *Il6*, *Tlr9*, *Col15a1*, *Col14a1*, and *Adam8* mRNAs, and the downregulation of *Camk2d*, *Gata4*, *Actn2*, and *Mfn2* mRNAs in *Gadlor*-KO CMs after TAC by RT-qPCR (Figures 8C and 8D). To rule out a reduced number of mitochondria as a reason for the downregulation of mitochondrial genes, we assessed the mitochondrial-to-nuclear DNA ratio, but detected no changes in *Gadlor*-KO versus WT mice, indicating a similar number of mitochondria under both conditions (Figure 8E). We next assessed the effects of *Gadlor1/2* overexpression on CM gene expression after PE stimulation (Figure 8F). *Gadlor1/2* overexpression in large parts revealed opposite effects of what was found in *Gadlor*-KO CMs, for example it promoted the expression of genes assigned to the gene ontology class "cardiac muscle contraction" (*Gata4*, *Camk2d*, *Rcan1.4*), which mainly exert pro-hypertrophic function in CMs. These effects were partially reversed upon treatment with KN93, indicating CaMKII-dependent effects of *Gadlor1/2* on pro-hypertrophic (and other) gene expression.

Comparing the effects of *Gadlor* KO in the different cardiac cell types after TAC revealed a strong overlap among upregulated and downregulated genes in FBs, ECs, and CMs (Figure S7). For example, *Gadlor* KO led to the upregulation of energy-coupled proton transport mitochondrial genes, actin filament associated genes, and mitotic cell-cycle-related genes in all three cell types, in addition to the upregulation of cardiac muscle contraction and blood vessel morphogenesis genes in two cell types. On the other hand, genes related to the negative regulation of cell differentiation, cartilage development, and the regulation of membrane potential were downregulated in all three cell types, while genes related to cell migration, inflammation, extracellular matrix organization, heart contraction, calcineurin/NFAT signaling, and mitochondrial organization were downregulated in at least two of the cell types (Figure S7). Therefore, *Gadlor1/2* effects on gene expression contain common, but also unique targets in different cardiac cell types.

DISCUSSION

Here, we describe two lncRNAs (termed *Gadlor1* and *Gadlor2*) as central regulators of cardiac failure during pressure overload. *Gadlor1* and *Gadlor2* were jointly upregulated in mouse and human failing hearts. In the serum of patients with aortic stenosis, high *GADLOR2* levels were associated with a low ejection fraction. Accordingly, enhanced experimental *Gadlor1/2* expression in mouse hearts triggered aggravated heart failure, myocardial hypertrophy, and fibrosis, while combined *Gadlor*-KO mice were protected from cardiac dysfunction, fibrosis, and vascular rarefaction during short and persisting pressure overload. Paradoxically, despite retaining better heart function and remodeling features, the complete lack of *Gadlor*

Figure 6. Secreted *Gadlor* lncRNAs are transferred to cardiomyocytes via endothelial-derived EVs

(A) Schematic representation of experimental design of EV (isolated from C166 ECs, supernatant) transfer to NRCMs or NRFBs. (B) Validation of *Gadlor1* and *Gadlor2* overexpression in isolated EVs. (C) RT-qPCR detection of *Gadlor1* and *Gadlor2* after transferring control EVs (isolated from Adβgal-treated ECs) or *Gadlor*-EVs (isolated from Ad*Gadlor1/2*-treated ECs) on neonatal rat cardiomyocytes (NRCMs) or neonatal rat cardiac fibroblasts (NRFBs). The samples were collected after 6 h of EV incubation. (D) Visualization of NRCMs that were incubated with PKH67-labeled (green) EVs isolated from C166 ECs for 6 and 24 h. Staining was performed with Phalloidin (red) and DAPI (blue). Scale bar, 50 μm. (E) Scheme of co-culture experiment with transwell (1 μm pore diameter, allows EV transfer). Mouse WT cardiac EC (MCEC) on top cultured with *Gadlor*-KO or WT CMs in the bottom well without direct cell contact. (F) RT-qPCR detection of *Gadlor1* and *Gadlor2* in isolated *Gadlor*-KO CMs and MCEC mouse ECs after 48 h of mono-culture or co-culture as indicated. (G–H) RT-qPCR detection of *Gadlor1* and *Gadlor2* in isolated adult *Gadlor*-KO CMs (G) or WT CMs (H) and MCECs that were incubated with GW4869 as indicated. Samples were collected after 48 h of mono-culture and co-culture. DMSO was used as vehicle control. (I) *Gadlor1* and *Gadlor2* expression in the cytosol and nucleus of the recipient NRCMs after EV transfer (as shown in A). Data are shown as mean ± SEM. Data normality was evaluated with Shapiro-Wilk test and p values were calculated with Student's t test or one-way ANOVA with Fisher's LSD post-hoc test were applied for comparing two or multiple groups, respectively. Two-way ANOVA with Fisher's LSD post-hoc test was applied for grouped analysis when applicable. *$p < 0.05$, **$p < 0.01$, ***$p < 0.001$, ****$p < 0.0001$.

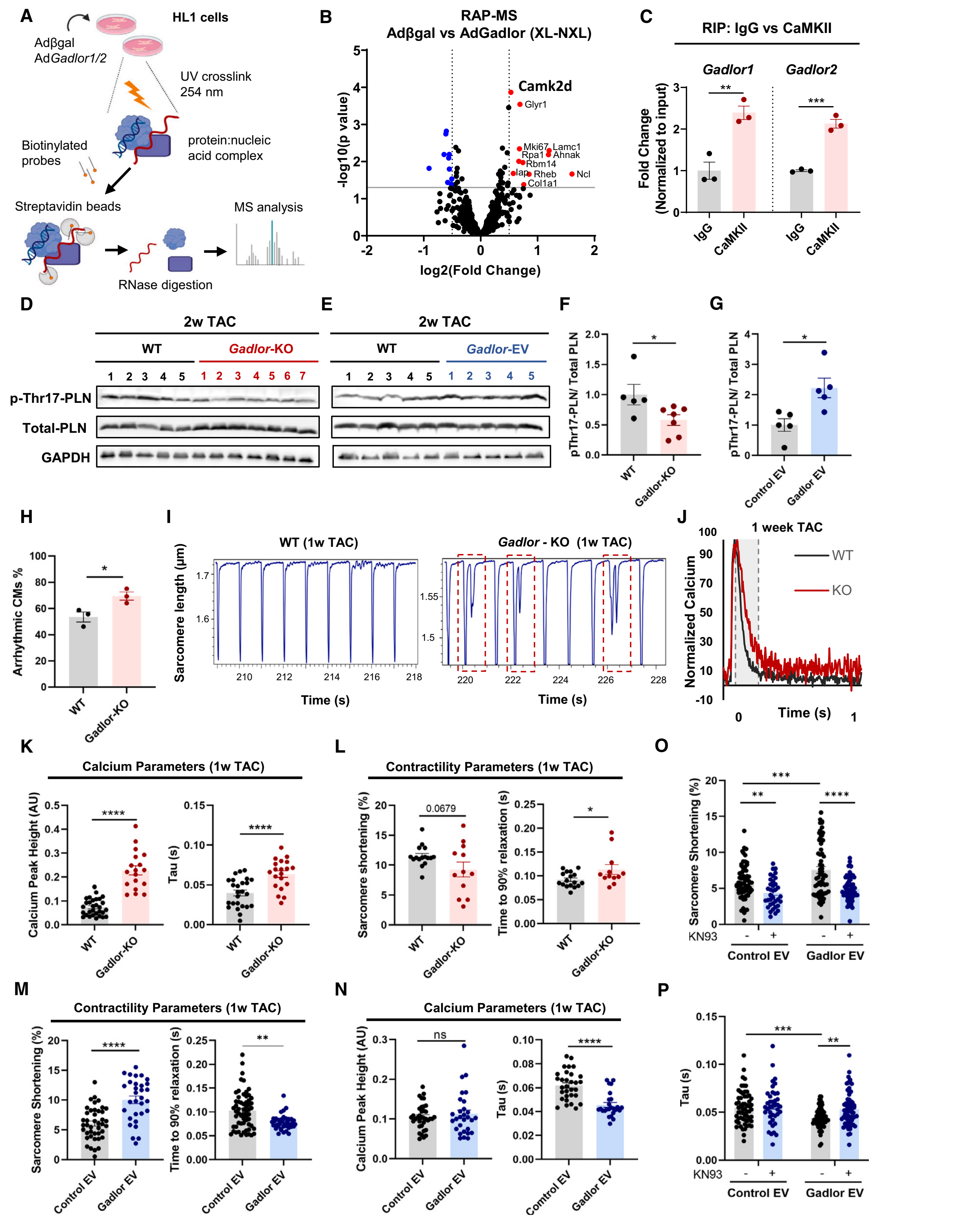

(legend on next page)

lncRNAs during chronic, persisting pressure overload entailed sudden death, most likely due to arrhythmia.

Among the different cell types in the myocardium, we found the highest *Gadlor1/2* expression in ECs, where reduced *Gadlor1/2* levels entailed an increased capillary density and cell proliferation after TAC. Importantly, enhanced myocardial angiogenesis was reported to promote cardiac function during pressure overload.[20,22] *Gadlor1/2* overexpression in isolated ECs reduced angiogenic activity, indicating that *Gadlor* lncRNAs inhibit angiogenesis in ECs in a cell autonomous manner. Because lncRNAs often exert their effects by modifying gene expression, we performed transcriptome profiling by RNA-seq in cardiac ECs after TAC. Indeed, we found that, upon *Gadlor1/2* deletion, ECs upregulate genes related to angiogenesis, cell cycle, and mitosis. Besides this cell-autonomous role, *Gadlor*-KO mice also exerted an increased expression of proangiogenic genes in CMs and FBs during TAC, which are both known to affect angiogenesis in a paracrine manner.[20,21,35,36]

In addition to ECs, cardiac FBs exhibited high levels of *Gadlor1/2* expression in the heart. Transcriptomic profiling in cardiac FBs revealed the downregulation of many extracellular matrix-associated genes, including pro-fibrotic growth factors, their receptors, as well as matrix proteins in *Gadlor*-KO mice. Our data imply that the pro-fibrotic roles of *Gadlor1* and *Gadlor2* are mediated by their direct effect on gene expression in cardiac FBs. Increased and decreased fibrosis will likely play a major role in reduced and improved heart function during TAC in mice with enhanced or ablated *Gadlor* expression, respectively.

Remarkably, *Gadlor1/2* are mainly secreted within EVs from ECs. This finding indicated a potential paracrine role of *Gadlor* lncRNAs in the heart. Indeed, EVs were reported to contain mainly miRNAs, but also lncRNAs and mRNAs, and to protect these RNA species from degradation by extracellular RNases and thus enhance RNA stability during transfer, leading to increased RNA accumulation in EVs when needed.[37] EVs might thereby play a role in maintaining homeostasis, and facilitating intercellular communication, especially during stress stimulation or disease conditions, when their production is typically upregulated.[38] In cell culture experiments, we found an effective uptake of *Gadlor1/2* from endothelial-derived EVs in neonatal and adult CMs. When adult CMs from *Gadlor*-KO mice that naturally could not upregulate intrinsic *Gadlor1/2* lncRNA expression were co-cultured in a two-chamber system (allowing the transfer of EVs, but not cells between the compartments) with wild-type ECs, these ECs in the upper well increased *Gadlor1/2* levels in the KOCMs at the bottom well, an effect that was blunted by inhibition of small EV generation. In addition, the co-cultured WT ECs markedly upregulated their *Gadlor1/2* expression compared with when they were cultured alone, which was also blunted by EV inhibition. Perhaps even more importantly, when WT ECs and WT CMs were co-cultured, EV inhibition strongly reduced *Gadlor1/2* levels in the WT CMs. This demonstrated that EC-derived EVs are crucial to provide *Gadlor1/2* to CMs, in which they regulate calcium cycling as well as gene expression. Our results therefore indicated a reciprocal intercellular signaling circuit between ECs and CMs, whereby ECs provide *Gadlor* lncRNAs within EVs to CMs, which signal back to ECs by currently unknown (perhaps EV-based) mechanisms to indicate insufficient *Gadlor1/2* levels, when necessary. Indeed, signal-responsive EV release had been described previously.[38] In conclusion, *Gadlor1/2* exert a strong effect in intercellular communication in the heart during pathological overload, on one hand by directly serving as intercellular messenger between ECs and CMs, and on the other hand by regulating the gene expression of secreted growth factors in CMs, ECs, and FBs, thereby affecting, for instance, angiogenesis and fibrosis.

In CMs, *Gadlor1/2* lncRNAs are taken up from ECs and localize to the cytosolic as well as to the nuclear compartment. Accordingly, we

Figure 7. *Gadlor* lncRNAs bind to calcium/calmodulin-dependent protein kinase type II in cardiomyocytes

(A) Experimental scheme of RNA-antisense purification coupled with mass spectroscopy (RAP-MS) to identify specific interaction partners of *Gadlor1/2* in HL1 cardiomyocytes treated with βgal or *Gadlor1/2* adenovirus. (B) Volcano plot indicating the identified interaction partners of *Gadlor1* and *Gadlor2* in cardiomyocytes with RAP-MS (fold change enrichment (FC) threshold 1.2 and $p < 0.05$). UV crosslinking was used to stabilize protein:RNA interactions and non-crosslinked (N-XL) samples were used as background and removed from crosslinked (XL) samples for each indicated condition. (C) RNA immunoprecipitation (RIP) performed with anti-IgG or anti-CaMKII antibodies from HL-1 cardiac muscle cell lysate to validate the interaction with *Gadlor1/2*. RT-qPCR analysis showing the enrichment of *Gadlor1* and *Gadlor2* compared with IgG controls. Data were normalized to input RNA expression. ($n = 3$ for each condition). (D and E) Western blots of phospho-Thr17-PLN and total PLN with GAPDH as loading control in (D) WT and *Gadlor*-KO and (E) in control-EV- and *Gadlor*-EV-treated mouse heart tissue samples after 2 weeks of TAC. (F and G) Quantification of wstern blots. (H) Percentage of isolated adult WT and *Gadlor*-KO cardiomyocytes with arrhythmic phenotype after 1 week TAC recorded using a Multicell HT system (Ion-Optix) Cell number ≥ 30 in each condition. (I) Exemplary traces of sarcomere shortening (contractions) of adult WT and *Gadlor*-KO cardiomyocytes isolated after 1 week of TAC (1w TAC). Images showing the measurement of transients in WT-CMs in which no arrhythmia was detected, and detection of arrhythmic beating shown as a secondary peak indicating an after-contraction before the end of the first contraction has been reached in *Gadlor*-KO CMs. Recordings of transients (nine transients in each measurement cycle) are obtained from a single cardiomyocyte. Images were analyzed with the CytoSolver transient analysis tool. (J–N) Sarcomere contractility and calcium transient measurements were recorded in parallel with the Ionoptix Multicell HT system with continuous perfusion of basal medium on isolated adult CMs after 1 week TAC. (J) Representative calcium transient recordings of WT and *Gadlor*-KO mice shown as normalized levels of calcium (%). (K) Calcium parameters including calcium peak height (arbitrary units, AU) and decay time (tau, s). (L) Contractility parameters including sarcomere shortening (%) and relaxation. Recordings were obtained from adult cardiomyocytes isolated from WT or KO mice after 1 week TAC. (M) Sarcomere function (shortening and relaxation) and (N) calcium peak height and decay time constant (tau). Recordings were obtained from adult cardiomyocytes isolated from WT mice after 1 week TAC followed by incubation of control-EV (Ad.βgal) and *Gadlor1/2*-EVs (Ad.*Gadlor1* and Ad.*Gadlor2*) for 4–6 h. (O and P) Sarcomere contractility and calcium tau measurements in cardiomyocytes as described before with and without addition of the CaMKII inhibitor KN93. Data are shown as mean ± SEM. Data normality was evaluated with Shapiro-Wilk test and p values were calculated with Student's t test (Mann-Whitney for non-parametric) for comparing two groups. $^{*}p < 0.05$, $^{**}p < 0.01$, $^{***}p < 0.001$, $^{****}p < 0.0001$.

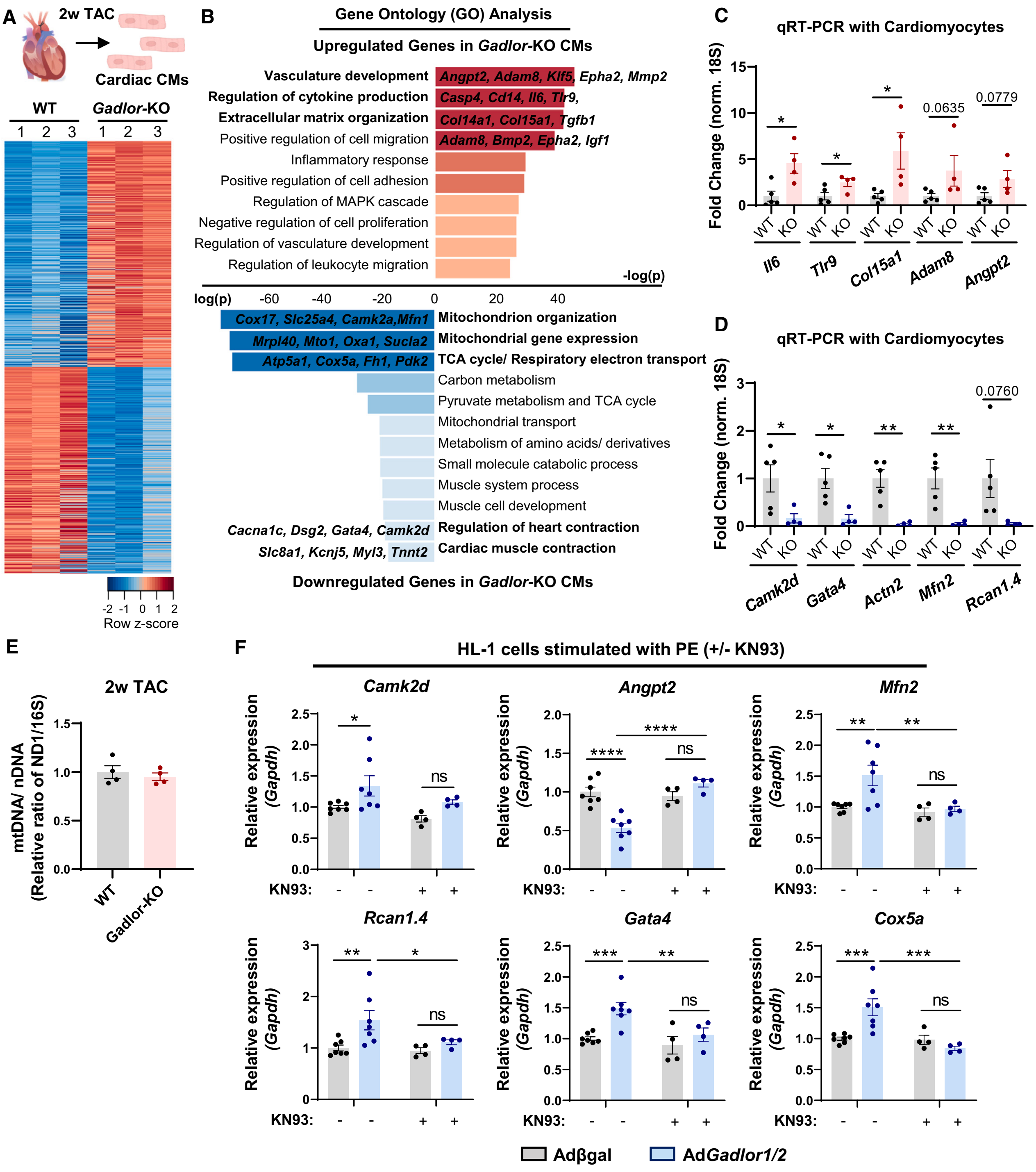

Figure 8. Deletion of *Gadlor1* and *Gadlor2* affects gene expression in cardiomyocytes

(A) Heatmap showing differentially regulated genes analyzed by bulk RNA-seq of isolated cardiomyocytes after 2 weeks TAC. (B) Bar plots showing the GO analysis of upregulated and downregulated genes in *Gadlor*-KO CMs after 2 weeks TAC (red, upregulated in *Gadlor*-KO; blue, downregulated in *Gadlor*-KO). Exemplary genes were listed for selected GO terms. (C and D) Validation of selected upregulated and downregulated genes from RNA-seq data with RT-qPCR. (E) Analysis of mitochondrial DNA (mtDNA) to nuclear DNA (nDNA) ratio determined via quantitative PCR by using WT and *Gadlor*-KO heart tissue samples isolated 2 weeks after TAC. Assessment of ND1 and 16S DNA abundance was used to determine the mtDNA/nDNA ratio. (F) RT-qPCR of selected genes in HL-1 cardiac muscle cells overexpressing *Gadlor* lncRNAs after

(legend continued on next page)

identified CaMKII as binding partner of *Gadlor1/2* in CMs, which localizes to both compartments and is activated mainly by calcium/calmodulin, autophosphorylation and oxidation, or S-nitrosylation.[39] Its activity is known to be about 3-fold upregulated in failing human hearts.[40] In CMs, CaMKII promotes on one hand maladaptive features such as hypertrophy and fibrosis by phosphorylating HDAC4, leading to de-repression of MEF2-dependent, pro-hypertrophic gene expression.[41,42] On the other hand, it has adaptive functions, for example, it promotes calcium re-uptake into the SR by phosphorylating phospholamban at Thr17.[43–45] Because *Gadlor1/2* bind CaMKII and *Gadlor*-KO mice exerted reduced, while *Gadlor1/2* overexpression led to enhanced Thr17 phosphorylation, we concluded that the *Gadlor* lncRNAs might promote CaMKII activation in a feed-forward loop in CMs during pathological overload. Decreased CaMKII activity could account for the improved cardiac remodeling features (i.e., reduced hypertrophy and improved systolic heart function) in *Gadlor*-KO mice, but at the same time slow down calcium re-uptake into the SR, leading to increased cytosolic calcium levels with pro-arrhythmic activity. Indeed, upon *Gadlor1/2* overexpression, pharmacological CaMKII inhibition partially reversed pro-hypertrophic gene expression and reversed increased calcium cycling in isolated CMs. Besides facilitating CaMKII activity by direct protein binding, *Gadlor1/2* also appear to promote *Camk2d* expression at the mRNA level. As another pro-hypertrophic gene, *Gata4* is also positively regulated by *Gadlor1/2* in CMs. Although details of how *Gadlor1/2* directly regulate gene expression in the CM nucleus will have to be addressed in future studies, this could work through binding to the chromatin reader protein GLYR1 (also known as NDF, NPAC, or NP60), which we also found as a direct binding partner of *Gadlor1/2* and that is strongly involved in the regulation of CM gene expression during differentiation by binding to the gene body of actively transcribed genes, often together with GATA4.[46]

In conclusion, upregulation of *Gadlor1/2* in failing hearts and their transfer into CMs might be a compensatory mechanism aiming at promoting calcium re-uptake into the SR during hemodynamic overload, although this comes at the cost of aggravated hypertrophy and fibrosis. Considering their dichotomous role, *Gadlor1/2* inhibition in CMs might therefore not be a suitable therapeutic strategy in heart failure. Even though this would lead to inhibition of hypertrophy, there might be the danger of arrhythmia. On the contrary, targeted inhibition of *Gadlor1/2* effects in ECs and FBs could be beneficial, as it would entail increased angiogenesis and reduced FB-mediated fibrosis, which according to our data are likely the main underlying reasons for improved heart function in *Gadlor*-KO mice. Although more work is needed, we suggest that the inhibition of *Gadlor* effects in cardiac non-myocytes might be a promising strategy to treat heart failure in the future.

Limitations of the study

As our study focuses on the systemic KO of *Gadlor1* and *Gadlor2* together, we acknowledge the limitation of not being able to investigate the individual effects of each lncRNA, as well as their cell-specific effects. While we extensively analyzed different isolated cell types to overcome this challenge, we recognize that generating cell-specific KO models would provide additional insights into the mechanisms of action. In addition, while we conducted thorough baseline cardiac phenotypic analysis in mice under normal physiological conditions, further investigation into the effects of *Gadlor1/2* lncRNAs on other organs, both in normal and disease conditions, is required.

We have identified CaMKII as a direct target of *Gadlor* lncRNAs in CMs, influencing calcium cycling and hypertrophy. To which degree the *Gadlor* effects *in vivo* depend on its impact on CaMKII will require further investigations. In addition, we have not yet identified their direct targets in ECs or FBs, which will be the focus of our future studies. Although we have elucidated a crucial role of *Gadlor1/2* under pathological conditions, their role in normal physiology remains unclear. We envision that *Gadlor1/2* may fine-tune the link between capillary and CM growth and function even in the absence of pathology in the adult heart, but this requires experimental validation in future studies.

Furthermore, validating our findings in models of human cells, such as human induced pluripotent stem cell-derived CMs in the future, will strengthen our findings and support the potential for future therapeutic studies.

MATERIALS AND METHODS

Materials and methods that are exclusively related to supplemental figures are provided in the extended methods in the supplemental information.

Human samples

Studies on human heart tissue samples were approved by the Institutional Ethical Board of Massachusetts General Hospital, where samples were collected from patients with end-stage heart failure undergoing cardiac transplantation.[47] Control heart tissue samples were obtained from healthy organ volunteers when the organ was not eligible for transplantation, or from the victims of traffic accidents. Human serum samples were obtained from healthy blood donors, or from aortic stenosis patients before replacement of the aortic valve (clinical data are available in Table S1), where all donors were provided a written informed consent for the collection and use of samples, that had received approval from the Institutional Review Board of Christian-Albrechts-Universität Kiel (file no. A174/09).

Gadlor1/2 adenovirus treatment (βgal as control) followed by phenylephrine (PE) stimulation (100 μM, 24 h) with and without KN93 (1 μM, 2 h) addition. Data are shown as mean ± SEM. Data normality was evaluated with Shapiro-Wilk test. p values were calculated with Student's t test for parametric (or Mann-Whitney for non-parametric) for comparing two groups. Two-way ANOVA with Fisher's LSD post-hoc test was applied for grouped analysis. ns, not significant, *$p < 0.05$, **$p < 0.01$, ***$p < 0.001$, ****$p < 0.0001$.

Measurement of GADLORs in human serum samples

Human serum samples were collected on the day before transcatheter aortic valve implantation. Serum RNA was extracted using an miRNeasy serum/plasma kit (no. 217184; QIAGEN) based on the manufacturer's instructions. LncRNA levels were normalized by the addition of 2 μL of TATAA universal RNA Spike (no. RS10SII; Tataa Biocenter) prior to RNA isolation. RNA was transcribed to cDNA (no. K1652; Maxima H Minus First Strand cDNA Synthesis Kit; Thermo Fisher) and analyzed with TaqMan Non-coding RNA Technology (no. 4369016) using custom-specific TaqMan Gene Expression Assay (no. 43331348, ID: APPRKET und APRWEYP; Thermo Fisher). For qRT-PCR, Stratagene's MX4000 multiplex qPCR system was used.

Animal models and studies

All studies including the use and care of animals were performed with the permission of the Regional Council Karlsruhe and the Lower Saxony State Office for Consumer Protection and Food Safety, Germany, with approved protocols 35-9185.81/G-144/18, I-22/03, 33.9-42502-12- 10/0016, 33.19-42502-04-14/1403, and 33.8-42502-04-16/2356. Female and male animals were used in similar proportions throughout the study, except for the experiments in Figure 3, where only male mice were used.

WT ICR/CD1 mice were in-bred in-house and C57BL/6N mice were obtained from Janvier Labs (Le Genest-Saint-Isle, France). Animals were maintained in a temperature-humidity controlled (22°C ± 2°C and 35%–60% humidity), 12-h dark-light cycled room with unlimited access to water and standard food.

Systemic *Gadlor*-KO mice were generated by deletion of the respective region of mouse chromosome 16 using a CRISPR-Cas9-based strategy to achieve homozygous deletion. In brief, embryonic stem cells (G4 line) were transfected with gRNAs LNC1-01 (target sequence: 5′-TTGTACATGAGCGGTTGTAG-3′) and LNC2-01 (target sequence 5′- AGTATACAGGGGGTTACCAT-3′) as well as *in-vitro*-transcribed CAS9-GFP mRNA.[48] GFP- expressing cells were sorted and single-cell-derived clones were established. Five out of 72 analyzed clones showed extensive deletions between the gRNA target sites. Clones 1–37 comprised a deletion between 39,995,533 and 40,004,426 on one chromosome 16 allele. This clone was injected into C57BL/6 blastocysts to generate a transgenic mouse line, which was then further crossed to achieve a homozygous deletion.

Pressure overload was induced in 8- to 10-week-old mice by TAC and maintained for 2 weeks for short-term and 8–12 weeks for long-term studies as described previously.[49] Analgesia (0.1 mg/kg buprenorphine) was provided by subcutaneous injection. Tracheal intubation was followed by upper thoracotomy to visualize the aortic arch, which was tied with a 7-0 silk ligature around a 26G needle that was removed immediately after secured constriction. Mice were injected with 0.1 mg/kg atropine, the chest wall was closed with suture, and then followed by closure of the skin with surgical glue. The mice were ventilated with 2% isoflurane throughout the procedure. For postoperative care, additional analgesia was provided in drinking water for the following 5 days. To assess the mortality rate, mice were followed and inspected daily after TAC. Sham-operated animals were treated with the same procedure and medications, but no constriction was applied to the aortic arch. Mice were monitored with echocardiography before and during pressure overload for assessment of cardiac function.

Transthoracic echocardiography

Echocardiography was performed with 1% isoflurane anesthesia. Mice were dorsally placed on a heated table to maintain the body temperature during the procedure while the ECG and the respiration rate were recorded continuously. Echocardiography was recorded with a linear 30- to 40-MHz transducer (MX-550D) and the Vevo 3100 system (VisualSonics, Toronto, Canada) as described.[50] Data were analyzed with the Vevo Lab 5.5.0 software.

Left ventricle and apical four chamber view

Parameters were recorded in B-mode and M-mode in both parasternal long-axis (PSLAX) and short-axis view at the papillary muscle level. Left-ventricular posterior wall thickness and left-ventricular end-diastolic volume were used to characterize LV microanatomy and the change of LV diameter length from end-diastole to end-systole was used to assess contractility and to calculate LV ejection fraction and fractional shortening, which were measured with the PSLAX mode in the analysis. B-Mode tracing in the apical four-chamber view was recorded at the atrioventricular valve level with pulsed-wave Doppler and tissue Doppler measurements.

EV-mediated *Gadlor1* and *Gadlor2* overexpression

As a gain-of-function approach, *Gadlor1* and *Gadlor2* were overexpressed in mouse hearts by administration of *Gadlor1/2*-enriched EVs. To this end, C166 ECs were infected with *Gadlor1* and *Gadlor2* adenoviruses for 48 h. EVs were collected with ExoQuick-TC solution (EXOTC50A-1,65 SystemsBio) based on manufacturers' protocols with some adjustments. *In vivo* overexpression of *Gadlor* lncRNAs was achieved by injecting EVs directly into the left ventricles before TAC surgery. The intraventricular injection was performed by simultaneous cross-clamping of the aorta and pulmonary artery distal of the origin of the coronary vessels.

Organ harvest

At the end of the experiment, mice were weighed and euthanized to collect the heart, lungs, and liver. Organs were washed in cold PBS to remove the blood, and additionally the heart was washed in 0.5% (w/v) KCl in PBS. The heart was transversely cut into half, and the upper layer was immediately embedded into OCT for histological analysis while the other half was snap frozen.

Histology

OCT-embedded organs were sectioned into slices of 7 and 12 μm for immunofluorescence and Picrosirius red staining, respectively. In brief, for immunofluorescence tissue sections were fixed with 4% PFA

followed by permeabilization with 0.3% Triton X-100 in PBS and blocking with 3% BSA. Primary and secondary antibodies that were used are listed in Table S3. Samples were visualized using a Leica DMi8 Fluorescence microscope. Images were analyzed with Leica Application Suite X (LAS X) 3.7. Sirius red staining was performed by fixing the tissue samples with 100% ice-cold acetone, and by following the standard protocol. Tissue sections were scanned with Zeiss Axio Scan.Z1. Images were analyzed with ZEN 2.6 Blue Edition (Carl Zeiss).

Isolation and culture of juvenile mouse ECs

Hearts from 7- to 12-day-old mice were collected and washed in ice-cold DMEM high-glucose to clean and remove the atria. The minced tissue was transferred into 5 mL of enzyme solution (for 3–4 hearts) and incubated with collagenase I (500 U/mL, Worthington, LS004176) and DNase I (150 U/mL, Worthington, LS002139) in HBSS (without Ca and Mg). After digestion, the lysate was washed with FCS and passed through a 70-μm cell strainer. The washed lysate was incubated with CD31 antibody (BD, 553370) coupled to Dynabeads (Invitrogen, 11035) for the first step of isolation, before the cells were plated on 0.5% gelatinized plates. For culturing, cells were maintained for 3–4 days until they reached 80%–90% confluency. During the first passage, further purification of cells was achieved by incubation with CD102 antibody (BD, 553370)-coupled Dynabeads. Primary ECs were maintained in DMEM with 20% FCS supplemented with non-essential amino acids and sodium pyruvate and passaged up to P3 for experiments.

Isolation of adult mouse ECs, FBs, and CMs

Isolation of ECs and FBs from adult mouse hearts was performed with MACS magnetic beads from Miltenyi Biotec. In brief, heart tissue was washed in ice-cold PBS and minced into small pieces before incubating in enzyme solution (collagenase I [500 U/mL, Worthington, LS004176] and DNase I [150 U/mL, Worthington, LS002139] in RPMI 1640 [Thermo Fischer Scientific, 31870025]). Digested tissue samples were passed through a 70-μm cell strainer and washed with FCS, and MACS buffer with BSA (Miltenyi Biotec, 130-091-222). After multiple washing steps, the tissue lysate was incubated with CD146 microbeads (Miltenyi Biotec, 130-092-007) before being passed through magnetic columns (Miltenyi Biotec, MS columns 130-042-201) for positive selection of ECs. The flowthrough was incubated with feeder removal microbeads (Miltenyi Biotech, 130-095-531) and eluted after multiple washing steps to collect FBs. Isolated cells were stored as frozen cell pellets for later RNA or protein isolation for corresponding experiments.

Isolation of adult cardiac myocytes was achieved by using a Langendorff perfusion system.[51] In brief, immediately after excision, the heart was placed into ice-cold 1× perfusion buffer (10× stock: 1,130 mM NaCl, 47 mM KCl, 6 mM KH_2PO_4, 6 mM Na_2HPO_4, 12 mM $MgSO_4 \cdot 7H_2O$, 120 mM $NaHCO_3$, 100 mM $KHCO_3$, 100 mM HEPES buffer solution, and 300 mM taurine) to clean the tissue around the aorta under a microscope. Then the aorta was placed onto a cannula (inner diameter 1 mm) with forceps and tied with silk suture to allow perfusion through the coronary arteries. Subsequently, the heart was perfused with enzyme solution containing Liberase DH (5 mg/mL, Roche, 5401089001), trypsin (1%, Gibco, 15090046), and $CaCl_2$ (100 mM) to digest the tissue at 37°C. Enzymatic digestion was ended with Stop I and II solutions containing perfusion buffer with FCS and $CaCl_2$ (10 mM). Digested heart tissue samples were passed through a 100-μm cell strainer. Then, the calcium concentration was gradually increased by manual administration to digested heart tissue with continuous gentle mixing. Then the cells were subjected to either IonOptix analysis, culturing, or direct RNA and protein extraction (after CM sedimentation).

Isolation of NRCMs

Hearts from 1- to 3-day-old rats were collected and washed with ice-cold 1× ADS (pH 7.35) to clean the blood and remove the atria. The ventricular parts were minced in 1× ADS and digested in enzyme solution containing collagenase Type II (Worthington, LS004176) and pancreatin (Sigma P3292). The resulting cells were loaded onto a Percoll gradient to separate CMs and non-CMs. NRCMs were seeded (4.0×10^5/well for 6-well plate) onto 0.5% gelatin-coated plates.

Cell culture

C166 (CRL-2581, ATCC) mouse embryonic ECs and NIH3T3 (CRL-1658, ATCC) mouse FB cells were cultured in Dulbecco's modified Eagle's medium (DMEM) with 10% FCS, while MCECs (CLU510-P, Tebu-bio), immortalized MCECs, were cultured with 5% FCS containing DMEM supplemented with L-glutamine (1%), penicillin-streptomycin (1%), and HEPES. HL-1 cardiac muscle cells (SCC065, Merck) were cultured with Claycomb medium (Sigma, 51800C) supplemented with 10% FCS, 0.1 mM norepinephrine, 2 mM L-glutamine, and 1× penicillin-streptomycin (P/S). HL-1 cells were plated on 0.1% gelatin-coated flasks, and cell culture medium was refreshed daily. Cells were cultured for up to 10 passages.

Adenoviral infection

For overexpression of *Gadlor* lncRNAs, recombinant adenoviruses were used. Mouse cDNAs of AK037972 (*Gadlor1*) and AK038629 (*Gadlor2*) were subcloned into the pShuttleCMV vector (Source Bioscience, UK) and adenoviruses were generated using the AdEasy Adenoviral Vector system (Agilent, 240009). An adenovirus overexpressing β-galactosidase (Adβgal) was used as a control. After the production of adenoviruses, the viral titer was calculated with the help of the AdEasy Viral Titer Kit (Agilent, 972500), and all the experiments were performed at a multiplicity of infection of 50. Adenoviral infection on cultured cells was performed for 4 h at 37°C on cells with medium containing heat-inactivated FCS.

Co-culture experiments

For co-culture experiments, adult mouse CMs isolated from *Gadlor*-KO mice were seeded on laminin-coated (Santa Cruz, SC-29012) 6-well plates and cultured with MCECs plated on 1 μm pore-sized inserts (Thincert, Greiner, 657610). Cells were collected separately after 48 h for RNA isolation.

To inhibit EV trafficking between MCECs and CMs in the co-culture model, MCECs were pre-treated with 20 μM of GW4869 (Hoelzel Biotech) for 6 h, and then placed for 48 h of co-culture with isolated *Gadlor*-KO or WT CMs.

EC sprouting assay

To study the effect of *Gadlor* lncRNAs on angiogenesis, we performed sprouting assays with C166 mouse ECs according to a well-established protocol described previously.[52] In brief, cells were infected with Ad.βgal or Ad.*Gadlor1/2* adenovirus for 24 h. A total of 50,000 cells were re-suspended with 4 mL DMEM + 10% FCS and 1 mL methylcellulose solution (Sigma-Aldrich, M0512) and drops containing 25 μL of suspension were pipetted onto a 10-cm cell culture dish. The drops were then incubated upside-down in a cell culture incubator for 24 h to allow spheroid formation. On the following day, the cell spheroids were gently washed off the hanging drops with PBS, collected by 200 × *g* centrifugation for 5 min and re-suspended with methylcellulose solution containing 20% FCS. The collagen matrix was prepared in parallel on ice with collagen stock solution (Corning, 11563550), diluted in 10× M199 medium (Sigma-Aldrich, M0650), and the required amount of sodium hydroxide to set the pH for polymerization. Then the collagen medium was mixed with the spheroids. One milliliter of the spheroid-collagen-methylcellulose mixtures was added per well in a 24-well plate and incubated in a cell culture incubator for 30 min to induce the polymerization of the collagen-methylcellulose matrix. The endothelial spheroids were stimulated with 100 μL of DMEM + 10% FCS, 25 ng/mL FGF2, or 10 ng/mL TGF-β1 by adding it dropwise to the collagen matrix. After 24 h, the sprouting assay was stopped by adding 1 mL of 4% paraformaldehyde to the wells. Spheroids were visualized with the 10× objective in bright-field microscopy and analyzed with ImageJ.

RNA isolation and RT-qPCR

RNA isolation from tissue samples was performed with QIAzol reagent (QIAGEN, 79306), and from isolated cells with a NucleoSpin RNA isolation kit (Macherey-Nagel, 740955.250) according to manufacturers' protocols. Samples were incubated with rDNase (Macherey-Nagel, 740963) on the silica columns to remove any DNA contamination. cDNA was generated by using the Maxima-H minus First strand cDNA synthesis kit (Thermo Fisher Scientific, K1652). qPCR was performed with the Maxima SYBR Green mix with ROX as reference dye (Thermo Scientific, K0253) on an AriaMx Real-time PCR System (Agilent, G8830a). Gene expression was normalized to Gapdh 18S or U6 expression. All qPCR primer sequences are listed in Table S4.

Protein isolation and western blotting

Heart tissue protein lysates were prepared from frozen pulverized tissue with lysis buffer containing 30 mM Tris (pH 8.8), 5 mM EDTA (pH 8.0), 3% SDS (v/v), 10% glycerol (m/v), protease, and phosphatase inhibitors. Protein quantification was performed by BCA assay (Thermo Fisher Scientific, Pierce BCA Protein Assay kit, 23225). Samples were incubated at 95°C for 5 min after the addition of Laemmli buffer (1× final concentration) and proteins were separated with SDS-PAGE electrophoresis. The following primary antibodies were used: Gapdh (Meridian Bioscience, H86045P, mouse), Phospholamban, pThr17 (Badrilla A010-13, rabbit), and Phospholamban (Badrilla, A010-14 mouse). The following secondary antibodies were used: rabbit anti-mouse peroxidase (Sigma-Aldrich) and goat anti-rabbit peroxidase (Sigma-Aldrich).

Sarcomere contractility and calcium transient measurements

Adult CMs were isolated 1 week after TAC operation. Cardiomyocytes were plated on laminin-coated (10 μg/cm^2) 35-mm dishes (MatTek 10-mm glass-bottom dishes, P35G-1.5-10-C) and gently washed 1 h later with MEM medium without butanedione monoxime. The plated cells were then transferred into the Ion-Optix Multicell High-Throughput (HT) system chamber, where they were simultaneously paced (18 V, 2.5 Hz, 4 ms impulse duration), and assessed for sarcomere length and contraction. Calcium handling was recorded after incubation with 1 μM fura-2, AM (Invitrogen, F1221).

For measurements following *Gadlor1/2* overexpression via EV-mediated transfer, isolated adult CMs were incubated with EVs for 4 h. To assess the effect of CaMKII inhibition, cells were treated with KN93 (1 μM) for 1 h before the measurements. The recordings were analyzed with IonWizard software.

RAP-MS

To identify the protein interaction partners of *Gadlor* lncRNAs we performed RAP-MS as described previously with minor modifications.[53] The 5′ biotinylated antisense *Gadlor1* and *Gadlor2* probes were pooled in the experiment and the sequences are listed in Table S5. We used HL-1 CMs overexpressing *Gadlor1* and *Gadlor2* after infection with Ad.*Gadlor1* and Ad.*Gadlor2*. RAP was performed with cells collected from five 15-cm dishes per sample, where each experimental group contained three replicates without (as a negative control to identify non-specific "background" proteins) and four replicates with UV crosslinked conditions. HL-1 cells were washed with cold PBS twice and crosslinked using 150 mJ/cm^2 of 254 nm UV light. Cells were then lysed with lysis buffer, incubated for 10 min on ice, homogenized by passing several times through a 21G needle and DNA was digested with addition of DNase salt solution and Turbo DNase for 10 min at 37°C (Thermo Fisher Scientific, AM2238). Hybridization conditions were adjusted by the addition of an equal amount of hybridization buffer. Lysates were precleared with streptavidin-coated magnetic beads. Biotin-labeled *Gadlor1* and *Gadlor2* probes were heated to 85°C for 3 min and then incubated with the lysate for 2 h at 67°C. Probe-RNA complexes were captured by pre-washed streptavidin-coated magnetic beads and incubated at 37°C for 30 min. Lysate was removed from beads by magnetic separation, and beads were washed four times in hybridization buffer at 67°C. *Gadlor* lncRNA-bound proteins were then released by Benzonase RNA digestion for 2 h at 37°C. The captured protein samples were identified by TMT labeling followed by liquid chromatography-tandem MS by the EMBL proteomics core facility.

RIP

We performed RIP with minor modifications to a previously described protocol to confirm the interaction of *Gadlor1* and *Gadlor2* lncRNAs with CaMKII.[54] HL-1 CMs were used to overexpress *Gadlor* lncRNAs by adenovirus treatment, while Ad.βgal-treated samples were used as control. Three 15-cm plates were combined for preparation of each sample. In brief, cells were washed with ice-cold PBS twice and lysed with polysome lysis buffer (100 mM KCl, 5 mM $MgCl_2$, 10 mM HEPES [pH 7], 0.5% IGEPAL CA-630, 0.1 mM DTT, 1× protease inhibitor cocktail, 1× RNasIN). Cell lysates were passed through a 26G needle multiple times for homogenization. Protein G magnetic beads (Bio-Rad SureBeads, 161-4221) were washed twice with NT-2 buffer (50 mM Tris-HCl [pH 7.4], 150 mM NaCl, 5 mM $MgCl_2$, and 0.05% IGEPAL CA-630) and coupled with anti-CaMKII antibody (Santa Cruz, sc-5306) or anti-IgG (mouse, Cell Signaling 7076, as control) overnight at 4°C. Antibody coupled beads were washed twice with NT-2 buffer and re-suspended in NET-2 buffer (50 mM Tris-HCl [pH 7.4], 150 mM NaCl, 5 mM $MgCl_2$, 0.05% IGEPAL CA-630, 20 mM EDTA [pH 8], 1 mM DTT, 1× RNaseIN). Cell lysates were added to beads and incubated at 4°C for 2 h (10% lysate was removed as input and stored on ice prior to IP).

After supernatant was removed from the beads, 1 mL of ice-cold NT-2 buffer was used to wash the beads five times in total. Then, beads were re-suspended with PK buffer (1× NT-2 buffer and 1% SDS) and incubated at 55°C for 30 min with constant shaking while input sample conditions were adjusted accordingly and processed in parallel. The supernatant was transferred into fresh tubes and combined with NT-2 buffer and UltraPure phenol-chloroform (Thermo Fisher Scientific, 15593031) for RNA purification. After centrifugation in heavy-lock tubes at 15,000 × *g* for 15 min, RNA was purified from the clear aqueous phase with the RNA Clean and concentrator kit (Zymo Research, R1017).

Isolation of EVs

Isolation of EVs was performed with ultracentrifugation from the supernatant of C166 cells that were cultured with EV-depleted FCS. Initially, cell debris and large vesicles (apoptotic bodies) were removed by centrifugations at 300 × *g* and 10,000 × *g*, respectively. Subsequently, small EVs (including microvesicles and exosomes) were collected with 100,000 × *g* ultracentrifugation for 90 min, and similarly washed with PBS, before a second round of centrifugation was performed. The EV pellet was re-suspended in PBS for NTA (ZetaView Nanoparticle Tracing Videomicroscope, PMX-120) measurements, or with QIAzol lysis buffer (QIAGEN, 79306) for RNA isolation.

EV-RNA isolation

RNA isolation from EVs was performed by RNA precipitation as described before.[55] In brief, the EV pellet was re-suspended with 1 mL of QIAzol, and then mixed with 200 μL of chloroform for phase separation. Samples were mixed thoroughly and incubated for 5 min at room temperature (RT), then centrifuged at 12,000 × *g* at 4°C for 15 min. The aqueous upper layer was mixed with 10% (v/v) sodium acetate (3M [pH 5.5]) and 4 μL of glycogen (5 mg/mL) in 1.5 mL ethanol (100%). Samples were incubated at −80°C overnight, then centrifuged at 16,000 × *g* for 30 min to pellet the RNA. The RNA pellet was then washed with 70% ethanol and dried, before being re-suspended in nuclease-free water.

EV RNase and PK protection assay

To test the transfer of *Gadlor* lncRNAs within EVs, we performed RNase and PK protection assays with and without prior the application of Triton X-100. In brief, EVs were isolated and treated with 100 ng/μL of RNase A and 20 mg/μL of PK for 30 min at 37°C. PMSF (5 mM) was used to inactivate the PK at RT. To disrupt the lipid bilayer, EVs were incubated with 1% Triton X-100 for 1 h for selected samples. As a negative control, RNA isolated from EVs was also treated with the same protocol.

EV-labeling with PKH67 cell linker

To visualize EVs for transfer and re-uptake experiments *in vitro*, they were labeled using the PHK67 Green Fluorescent Cell Linker kit (Sigma, MIDI67). In brief, the EV pellet was re-suspended with 1 mL of diluent C including 4 μL of PKH67 dye, and incubated for 5 min at RT. Then, 1% BSA-PBS was added into the suspension to remove the unspecific binding of the dye. Then EVs were pelleted at 3,000 × *g* for 1 h and then suspended with the cell culture medium and added on NRCMs. Visualization of EVs was conducted with a Leica Confocal Microscope TCS SP8 and images were analyzed by Leica Application Suite X (LAS X) 3.7.

RNA-seq and bioinformatics

RNA isolation was performed from isolated cells as described at the indicated time points (2 weeks after TAC or sham surgery). Quality control of RNA samples (Agilent 2100 Fragment Analyzer) and library preparation (DNBSEQ Eukaryotic Strand-specific mRNA library) were performed by BGI, Hong Kong. Bulk RNA-seq from different cardiac cells was performed as stranded and single-end with 50-base sequence read length by BGI with an Illumina HiSeq 2500. Initially, the trimming of adapter sequences from fastq files was performed with the R package FastqCleaner. For aligning the reads to the reference genome (mm10) after trimming, the R package bowtie2 alignment tool was used. Gene annotation was performed with the bioMaRt R package. Library size of the samples was normalized to counts per million (cpm) and transformed into log2 values followed by calculation of differential gene expression with the edgeR package of R. Significant changes in gene expression between compared groups were filtered based on FDR < 0.05 and fold change > 1.5. Gene ontology analysis was performed with Metascape and DAVID online tools. Heatmaps that show the differentially regulated genes were generated by heatmap.2 function in ggplot2 library in R. The graphical abstract was created using BioRender.com.

Statistical analysis

Data analysis and statistical analysis were performed with GraphPad Prism software (v.8). Data are shown as mean ± standard error of the

mean (SEM). All the experiments were carried out in at least three biological replicates. The number of replicates for animal experiments and cell culture experiments are indicated in the figure legends. The investigators were blinded for mouse genotype and treatment during surgeries, echocardiography, organ weight determination, and all histological and immunofluorescence quantifications. Premature death was a criterion for exclusion from an ongoing 2-week TAC experiment. Death rates were not significantly different between experimental 2-week TAC groups.

Initially, all the datasets were analyzed for normality to allow the application of the proper statistical test. An unpaired two-tailed Student t test was used for comparing two groups only. Comparing multiple groups for one condition was performed with one-way ANOVA and Fisher's LSD post-hoc test. Comparing multiple groups for multiple conditions was achieved with two-way ANOVA and followed by Fisher's LSD post-hoc test when applicable. For non-parametric datasets, the Mann-Whitney and Kruksal-Willis tests were used to compare two groups and multiple groups, respectively. Values of $p < 0.05$ were considered statistically significant.

DATA AND CODE AVAILABILITY

The authors declare that the data supporting the findings of this study are available within the paper and its supplemental information. RNA-seq datasets were deposited in National Center for Biotechnology Information's (NCBI) Gene Expression Omnibus (GEO) repository with accession number GEO: GSE213612.

ACKNOWLEDGMENTS

The authors thank the Core Facilities Pre-Clinical Models, Live Cell Imaging Mannheim (LIMA), and the Transgenic Mice Service Unit (TGSM) at Helmholtz Center for Infection Research (HZI) for generating the knockout mice. The authors also acknowledge EMBL Proteomics Core Facility for performing proteomics analysis. This study was supported by grants from SFB1366 (CRC1366 – Vascular Control of Organ Function)/1 and 2-A6 (to J.H.) by the Deutsche Forschungsgemeinschaft (DFG). J.H. is also supported by SFB1550 (CRC1550-Molecular circuits of heart disease)/1-B01 by the Deutsche Forschungsgemeinschaft (DFG).

AUTHOR CONTRIBUTIONS

M.K. designed and performed the experiments, analyzed RNA-seq data, and wrote the first draft of the manuscript. S.G. designed and performed the experiments and analyzed data. N. Froese, F.A.T., R.W., S.H., S.L., G.M.D., M.S., and R.H. performed the experiments. D.W. designed and supervised the generation of *Gadlor*-KO mice. N.W. maintained the mouse colonies and performed experiments. P.-S.K., J. Hegermann, D.F., S.U., K.B., A.M.-G., and J.C. provided experimental support for the study. N. Frey, J.B., G.D., and T.W. provided crucial advice for the study and critically revised the paper. J.H. designed and conceptualized the study, supervised all the experiments, and prepared the final draft of the manuscript. All authors read and approved the manuscript.

DECLARATION OF INTERESTS

The patent for the use of lncRNA *Gadlor1* and *Gadlor2* in treating and preventing cardiac remodeling has been granted to N. Froese, J.B., and J.H. (US11208656, granted on 28.01.2021).

SUPPLEMENTAL INFORMATION

Supplemental information can be found online at https://doi.org/10.1016/j.omtn.2024.102306.

REFERENCES

1. Tsao, C.W., Aday, A.W., Almarzooq, Z.I., Alonso, A., Beaton, A.Z., Bittencourt, M.S., Boehme, A.K., Buxton, A.E., Carson, A.P., Commodore-Mensah, Y., et al. (2022). Heart Disease and Stroke Statistics-2022 Update: A Report From the American Heart Association. Circulation *145*, e153–e639.
2. Frantz, S., Hundertmark, M.J., Schulz-Menger, J., Bengel, F.M., and Bauersachs, J. (2022). Left ventricular remodelling post-myocardial infarction: pathophysiology, imaging, and novel therapies. Eur. Heart J. *43*, 2549–2561.
3. Wu, X., Reboll, M.R., Korf-Klingebiel, M., and Wollert, K.C. (2021). Angiogenesis after acute myocardial infarction. Cardiovasc. Res. *117*, 1257–1273.
4. Frey, N., Katus, H.A., Olson, E.N., and Hill, J.A. (2004). Hypertrophy of the heart: a new therapeutic target? Circulation *109*, 1580–1589.
5. Bar, C., Chatterjee, S., Falcao Pires, I., Rodrigues, P., Sluijter, J.P.G., Boon, R.A., Nevado, R.M., Andrés, V., Sansonetti, M., de Windt, L., et al. (2020). Non-coding RNAs: update on mechanisms and therapeutic targets from the ESC Working Groups of Myocardial Function and Cellular Biology of the Heart. Cardiovasc. Res. *116*, 1805–1819.
6. Hill, J.A., and Olson, E.N. (2008). Cardiac plasticity. N. Engl. J. Med. *358*, 1370–1380.
7. Heineke, J., and Molkentin, J.D. (2006). Regulation of cardiac hypertrophy by intracellular signalling pathways. Nat. Rev. Mol. Cell Biol. *7*, 589–600.
8. Huang, C.K., Kafert-Kasting, S., and Thum, T. (2020). Preclinical and Clinical Development of Noncoding RNA Therapeutics for Cardiovascular Disease. Circ. Res. *126*, 663–678.
9. Lu, D., and Thum, T. (2019). RNA-based diagnostic and therapeutic strategies for cardiovascular disease. Nat. Rev. Cardiol. *16*, 661–674.
10. Wilusz, J.E., Sunwoo, H., and Spector, D.L. (2009). Long noncoding RNAs: functional surprises from the RNA world. Genes Dev. *23*, 1494–1504.
11. Li, H., Trager, L.E., Liu, X., Hastings, M.H., Xiao, C., Guerra, J., To, S., Li, G., Yeri, A., Rodosthenous, R., et al. (2022). lncExACT1 and DCHS2 Regulate Physiological and Pathological Cardiac Growth. Circulation *145*, 1218–1233.
12. Zhao, Y., Riching, A.S., Knight, W.E., Chi, C., Broadwell, L.J., Du, Y., Abdel-Hafiz, M., Ambardekar, A.V., Irwin, D.C., Proenza, C., et al. (2022). Cardiomyocyte-Specific Long Noncoding RNA Regulates Alternative Splicing of the Triadin Gene in the Heart. Circulation *146*, 699–714.
13. Viereck, J., Kumarswamy, R., Foinquinos, A., Xiao, K., Avramopoulos, P., Kunz, M., Dittrich, M., Maetzig, T., Zimmer, K., Remke, J., et al. (2016). Long noncoding RNA Chast promotes cardiac remodeling. Sci. Transl. Med. *8*, 326ra22.
14. Han, P., Li, W., Lin, C.H., Yang, J., Shang, C., Nuernberg, S.T., Jin, K.K., Xu, W., Lin, C.Y., Lin, C.J., et al. (2014). A long noncoding RNA protects the heart from pathological hypertrophy. Nature *514*, 102–106.
15. Trembinski, D.J., Bink, D.I., Theodorou, K., Sommer, J., Fischer, A., van Bergen, A., Kuo, C.C., Costa, I.G., Schürmann, C., Leisegang, M.S., et al. (2020). Aging-regulated anti-apoptotic long non-coding RNA Sarrah augments recovery from acute myocardial infarction. Nat. Commun. *11*, 2039.
16. Micheletti, R., Plaisance, I., Abraham, B.J., Sarre, A., Ting, C.C., Alexanian, M., Maric, D., Maison, D., Nemir, M., Young, R.A., et al. (2017). The long noncoding RNA Wisper controls cardiac fibrosis and remodeling. Sci. Transl. Med. *9*, eaai9118.
17. Cheng, Y., Wang, X., Yang, J., Duan, X., Yao, Y., Shi, X., Chen, Z., Fan, Z., Liu, X., Qin, S., et al. (2012). A translational study of urine miRNAs in acute myocardial infarction. J. Mol. Cell. Cardiol. *53*, 668–676.
18. Kuwabara, Y., Ono, K., Horie, T., Nishi, H., Nagao, K., Kinoshita, M., Watanabe, S., Baba, O., Kojima, Y., Shizuta, S., et al. (2011). Increased microRNA-1 and microRNA-133a levels in serum of patients with cardiovascular disease indicate myocardial damage. Circ. Cardiovasc. Genet. *4*, 446–454.
19. Schulte, C., Barwari, T., Joshi, A., Zeller, T., and Mayr, M. (2020). Noncoding RNAs versus Protein Biomarkers in Cardiovascular Disease. Trends Mol. Med. *26*, 583–596.
20. Heineke, J., Auger-Messier, M., Xu, J., Oka, T., Sargent, M.A., York, A., Klevitsky, R., Vaikunth, S., Duncan, S.A., Aronow, B.J., et al. (2007). Cardiomyocyte GATA4 functions as a stress-responsive regulator of angiogenesis in the murine heart. J. Clin. Invest. *117*, 3198–3210.
21. Sano, M., Minamino, T., Toko, H., Miyauchi, H., Orimo, M., Qin, Y., Akazawa, H., Tateno, K., Kayama, Y., Harada, M., et al. (2007). p53-induced inhibition of Hif-1 causes cardiac dysfunction during pressure overload. Nature *446*, 444–448.

22. Oka, T., Akazawa, H., Naito, A.T., and Komuro, I. (2014). Angiogenesis and cardiac hypertrophy: maintenance of cardiac function and causative roles in heart failure. Circ. Res. *114*, 565–571.

23. Sluijter, J.P.G., Verhage, V., Deddens, J.C., van den Akker, F., and Doevendans, P.A. (2014). Microvesicles and exosomes for intracardiac communication. Cardiovasc. Res. *102*, 302–311.

24. Pinto, A.R., Ilinykh, A., Ivey, M.J., Kuwabara, J.T., D'Antoni, M.L., Debuque, R., Chandran, A., Wang, L., Arora, K., Rosenthal, N.A., and Tallquist, M.D. (2016). Revisiting Cardiac Cellular Composition. Circ. Res. *118*, 400–409.

25. Uchida, S., and Dimmeler, S. (2015). Long noncoding RNAs in cardiovascular diseases. Circ. Res. *116*, 737–750.

26. Kenneweg, F., Bang, C., Xiao, K., Boulanger, C.M., Loyer, X., Mazlan, S., Schroen, B., Hermans-Beijnsberger, S., Foinquinos, A., Hirt, M.N., et al. (2019). Long Noncoding RNA-Enriched Vesicles Secreted by Hypoxic Cardiomyocytes Drive Cardiac Fibrosis. Mol. Ther. Nucleic Acids *18*, 363–374.

27. Piccoli, M.T., Gupta, S.K., Viereck, J., Foinquinos, A., Samolovac, S., Kramer, F.L., Garg, A., Remke, J., Zimmer, K., Batkai, S., and Thum, T. (2017). Inhibition of the Cardiac Fibroblast-Enriched lncRNA Meg3 Prevents Cardiac Fibrosis and Diastolic Dysfunction. Circ. Res. *121*, 575–583.

28. Hosen, M.R., Li, Q., Liu, Y., Zietzer, A., Maus, K., Goody, P., Uchida, S., Latz, E., Werner, N., Nickenig, G., and Jansen, F. (2021). CAD increases the long noncoding RNA PUNISHER in small extracellular vesicles and regulates endothelial cell function via vesicular shuttling. Mol. Ther. Nucleic Acids *25*, 388–405.

29. Bang, C., Batkai, S., Dangwal, S., Gupta, S.K., Foinquinos, A., Holzmann, A., Just, A., Remke, J., Zimmer, K., Zeug, A., et al. (2014). Cardiac fibroblast-derived microRNA passenger strand-enriched exosomes mediate cardiomyocyte hypertrophy. J. Clin. Invest. *124*, 2136–2146.

30. Hosen, M.R., Goody, P.R., Zietzer, A., Xiang, X., Niepmann, S.T., Sedaghat, A., Tiyerili, V., Chennupati, R., Moore, J.B., 4th, Boon, R.A., et al. (2022). Circulating MicroRNA-122-5p Is Associated With a Lack of Improvement in Left Ventricular Function After Transcatheter Aortic Valve Replacement and Regulates Viability of Cardiomyocytes Through Extracellular Vesicles. Circulation *146*, 1836–1854. https://doi.org/10.1161/CIRCULATIONAHA.122.060258.

31. Huang, G., Garikipati, V.N.S., Zhou, Y., Benedict, C., Houser, S.R., Koch, W.J., and Kishore, R. (2020). Identification and Comparison of Hyperglycemia-Induced Extracellular Vesicle Transcriptome in Different Mouse Stem Cells. Cells *9*, 2098.

32. Froese, N., Szaroszyk, M., Korf-Klingebiel, M., Koch, K., Schmitto, J.D., Geffers, R., Hilfiker-Kleiner, D., Riehle, C., Wollert, K.C., Bauersachs, J., and Heineke, J. (2022). Endothelial Cell GATA2 Modulates the Cardiomyocyte Stress Response through the Regulation of Two Long Non-Coding RNAs. Biology *11*, 1736.

33. Froese, N., Cordero, J., Abouissa, A., Trogisch, F.A., Grein, S., Szaroszyk, M., Wang, Y., Gigina, A., Korf-Klingebiel, M., Bosnjak, B., et al. (2022). Analysis of myocardial cellular gene expression during pressure overload reveals matrix based functional intercellular communication. iScience *25*, 103965.

34. Das, S., Shah, R., Dimmeler, S., Freedman, J.E., Holley, C., Lee, J.M., Moore, K., Musunuru, K., Wang, D.Z., Xiao, J., et al. (2020). Noncoding RNAs in Cardiovascular Disease: Current Knowledge, Tools and Technologies for Investigation, and Future Directions: A Scientific Statement From the American Heart Association. Circ. Genom. Precis. Med. *13*, e000062.

35. Dittrich, G.M., Froese, N., Wang, X., Kroeger, H., Wang, H., Szaroszyk, M., Malek-Mohammadi, M., Cordero, J., Keles, M., Korf-Klingebiel, M., et al. (2021). Fibroblast GATA-4 and GATA-6 promote myocardial adaptation to pressure overload by enhancing cardiac angiogenesis. Basic Res. Cardiol. *116*, 26.

36. Vidal, R., Wagner, J.U.G., Braeuning, C., Fischer, C., Patrick, R., Tombor, L., Muhly-Reinholz, M., John, D., Kliem, M., Conrad, T., et al. (2019). Transcriptional heterogeneity of fibroblasts is a hallmark of the aging heart. JCI Insight *4*, e131092.

37. Kim, K.M., Abdelmohsen, K., Mustapic, M., Kapogiannis, D., and Gorospe, M. (2017). RNA in extracellular vesicles. Wiley Interdiscip. Rev. RNA *8*.

38. Pironti, G., Strachan, R.T., Abraham, D., Mon-Wei Yu, S., Chen, M., Chen, W., Hanada, K., Mao, L., Watson, L.J., and Rockman, H.A. (2015). Circulating Exosomes Induced by Cardiac Pressure Overload Contain Functional Angiotensin II Type 1 Receptors. Circulation *131*, 2120–2130.

39. Mattiazzi, A., Bassani, R.A., Escobar, A.L., Palomeque, J., Valverde, C.A., Vila Petroff, M., and Bers, D.M. (2015). Chasing cardiac physiology and pathology down the CaMKII cascade. Am. J. Physiol. Heart Circ. Physiol. *308*, H1177–H1191.

40. Kirchhefer, U., Schmitz, W., Scholz, H., and Neumann, J. (1999). Activity of cAMP-dependent protein kinase and Ca2+/calmodulin-dependent protein kinase in failing and nonfailing human hearts. Cardiovasc. Res. *42*, 254–261.

41. Backs, J., Backs, T., Neef, S., Kreusser, M.M., Lehmann, L.H., Patrick, D.M., Grueter, C.E., Qi, X., Richardson, J.A., Hill, J.A., et al. (2009). The delta isoform of CaM kinase II is required for pathological cardiac hypertrophy and remodeling after pressure overload. Proc. Natl. Acad. Sci. USA *106*, 2342–2347.

42. Li, C., Cai, X., Sun, H., Bai, T., Zheng, X., Zhou, X.W., Chen, X., Gill, D.L., Li, J., and Tang, X.D. (2011). The deltaA isoform of calmodulin kinase II mediates pathological cardiac hypertrophy by interfering with the HDAC4-MEF2 signaling pathway. Biochem. Biophys. Res. Commun. *409*, 125–130.

43. Beckendorf, J., van den Hoogenhof, M.M.G., and Backs, J. (2018). Physiological and unappreciated roles of CaMKII in the heart. Basic Res. Cardiol. *113*, 29.

44. Mattiazzi, A., and Kranias, E.G. (2014). The role of CaMKII regulation of phospholamban activity in heart disease. Front. Pharmacol. *5*, 5.

45. Kemi, O.J., Ellingsen, O., Ceci, M., Grimaldi, S., Smith, G.L., Condorelli, G., and Wisløff, U. (2007). Aerobic interval training enhances cardiomyocyte contractility and Ca2+ cycling by phosphorylation of CaMKII and Thr-17 of phospholamban. J. Mol. Cell. Cardiol. *43*, 354–361.

46. Gonzalez-Teran, B., Pittman, M., Felix, F., Thomas, R., Richmond-Buccola, D., Hüttenhain, R., Choudhary, K., Moroni, E., Costa, M.W., Huang, Y., et al. (2022). Transcription factor protein interactomes reveal genetic determinants in heart disease. Cell *185*, 794–814.e30.

47. Haq, S., Choukroun, G., Lim, H., Tymitz, K.M., del Monte, F., Gwathmey, J., Grazette, L., Michael, A., Hajjar, R., Force, T., and Molkentin, J.D. (2001). Differential activation of signal transduction pathways in human hearts with hypertrophy versus advanced heart failure. Circulation *103*, 670–677.

48. George, S.H.L., Gertsenstein, M., Vintersten, K., Korets-Smith, E., Murphy, J., Stevens, M.E., Haigh, J.J., and Nagy, A. (2007). Developmental and adult phenotyping directly from mutant embryonic stem cells. Proc. Natl. Acad. Sci. USA *104*, 4455–4460.

49. Szaroszyk, M., Kattih, B., Martin-Garrido, A., Trogisch, F.A., Dittrich, G.M., Grund, A., Abouissa, A., Derlin, K., Meier, M., Holler, T., et al. (2022). Skeletal muscle derived Musclin protects the heart during pathological overload. Nat. Commun. *13*, 149.

50. Trogisch, F.A., Abouissa, A., Keles, M., Birke, A., Fuhrmann, M., Dittrich, G.M., Weinzierl, N., Wink, E., Cordero, J., Elsherbiny, A., et al. (2024). Endothelial cells drive organ fibrosis in mice by inducing expression of the transcription factor SOX9. Sci. Transl. Med. *16*, eabq4581.

51. Louch, W.E., Sheehan, K.A., and Wolska, B.M. (2011). Methods in cardiomyocyte isolation, culture, and gene transfer. J. Mol. Cell. Cardiol. *51*, 288–298.

52. Tetzlaff, F., and Fischer, A. (2018). Human Endothelial Cell Spheroid-based Sprouting Angiogenesis Assay in Collagen. Bio. Protoc. *8*, e2995.

53. McHugh, C.A., and Guttman, M. (2018). RAP-MS: A Method to Identify Proteins that Interact Directly with a Specific RNA Molecule in Cells. Methods Mol. Biol. *1649*, 473–488.

54. Gagliardi, M., and Matarazzo, M.R. (2016). RIP: RNA Immunoprecipitation. Methods Mol. Biol. *1480*, 73–86.

55. Prendergast, E.N., de Souza Fonseca, M.A., Dezem, F.S., Lester, J., Karlan, B.Y., Noushmehr, H., Lin, X., and Lawrenson, K. (2018). Optimizing exosomal RNA isolation for RNA-Seq analyses of archival sera specimens. PLoS One *13*, e0196913.

OMTN, Volume *35*

Supplemental information

Endothelial derived, secreted long non-coding RNAs *Gadlor1* and *Gadlor2* aggravate cardiac remodeling

Merve Keles, Steve Grein, Natali Froese, Dagmar Wirth, Felix A. Trogisch, Rhys Wardman, Shruthi Hemanna, Nina Weinzierl, Philipp-Sebastian Koch, Stefanie Uhlig, Santosh Lomada, Gesine M. Dittrich, Malgorzata Szaroszyk, Ricarda Haustein, Jan Hegermann, Abel Martin-Garrido, Johann Bauersachs, Derk Frank, Norbert Frey, Karen Bieback, Julio Cordero, Gergana Dobreva, Thomas Wieland, and Joerg Heineke

SUPPLEMENTAL MATERIAL

Extended Methods

Carotid flow measurement

To evaluate the degree of aortic constriction during TAC surgery, the peak velocity of flow in the right and left common carotid arteries (RCCA and LCCA) were measured 2 days after surgery with PW-Doppler at the level of carotid bifurcation to calculate the ratio of RCCA/LCCA flow.

EV FACS Staining

Characterization of surface markers of EVs was performed with flow cytometry by staining EC-derived EVs with CD9-PerCP-Cy5.5 (Miltenyi Biotec, 130-102-278, Clone: MZ3), CD63-APC (Miltenyi Biotec, 130-108-894, Clone: REA563) and CD54-FITC (BD, 553252, Clone: 3E2) labelled antibodies for 1 hour at 4°C. Measurements were obtained with BD FACSCanto ll.

EV Transmission Electron Microscopy (TEM)

Visualization of EVs was performed with TEM. After centrifugation, pellets were resuspended in the minimum possible volume of residual liquid and 25 % aqueous glutaraldehyde was added to a final concentration of 1 %. After fixation overnight, samples were mixed 1:1 (v/v) with 4 % agar at 40 °C. After hardening, the agar blocks were cut into cubes of 1 mm in size. Further preparation of samples and electron microscopy were performed as described, before being imaged with a FEI Morgagni 268 transmission electron microscope (FEI, Eindhoven, Netherlands) operated at 80 kV using a Veleta CCD camera (Olympus Soft Imaging Solutions).[1]

RNAScope in situ Hybridization

In situ hybridization was performed to localize *Gadlor1* (Mm-LOC118567341-C3), *Gadlor2* (Mm- AK038629-C1, Cat no: 404391) and *Cdh5* (Mm-Cdh5-C2, Cat no: 312531) mRNA expression on mouse heart tissue slides with RNAscope Multiplex Fluorescent v2 kit. The assay was performed according to the manufacturer's protocol. Briefly, OCT-embedded tissue slides (7 µm thickness) were used in the assay, fixed with 4% PFA and then stepwise dehydration was performed with increasing concentrations of ethanol. Tissue slides were subsequently treated with hydrogen peroxide (10 min at RT) and protease IV (30 min at RT) in the humidity control incubator. Slides were incubated for 2 hours with the mixture of probes to hybridize with the corresponding RNAs. Next, the signal was amplified with RNAScope Multiplex FL v2 AMP 1-3 reagents and then HRP channels were developed sequentially. Finally, DAPI was used to counterstain the slides, and each was mounted with ProLong Gold Antifade Mountant (Fisher Scientific).

Proximity Ligation Assay (PLA)

We performed proximity ligation assay (PLA) with minor modifications to a previously described protocol to confirm the interaction of *Gadlor1* and *Gadlor2* lncRNAs with CaMKII.[2] Briefly, specific single-stranded DNA probes, identical to those used in RAP-MS, were designed to be complementary to *Gadlor1* and *Gadlor2* RNA, each with a 3' biotin tag. For each target, five specific probes were pooled based on their region within the target RNA. HL-1 cells were fixed with 4% paraformaldehyde, washed in PBS, and permeabilized using PBS with 1% BSA and 0.1% Triton X-100. After washing in 0.1 M triethanolamine, acetic anhydride was incrementally added to a final concentration of 0.5%. Cells were washed in PBS with 0.02% Tween 20, followed by a hybridization buffer (5× SSC, 1× Denhardt's reagent, 0.1% Tween 20, 0.1% CHAPS, 5 mM EDTA, 1 mg/mL RNase-free tRNA, and 100 µg/mL heparin). Probes (200 nmol/L) were boiled at 95°C and incubated with cells in hybridization buffer overnight at 37°C in a humidified chamber.

Slides were sequentially washed in: 50% deionized formamide/5× SSC, 25% deionized formamide/1× SSC, 12.5% deionized formamide/2× SSC, 2× SSC/0.1% Tween 20, and 0.2× SSC/0.1% Tween 20. After washing in PBS with 0.02% Tween 20, slides were blocked with Duolink blocking buffer.

Primary antibodies (Rabbit-anti CamkIIΔ) were diluted in Duolink antibody diluent and incubated overnight at 4°C. After washing with Duolink buffer A, Duolink probes (Rabbit MINUS and Mouse PLUS) were applied to the slides for 1 hour at 37°C. Slides were washed in buffer A, treated with Duolink ligase mix for 30 minutes at 37°C, followed by a polymerase/amplification mix for 100 minutes at 37°C. Finally, slides were washed in buffer B, rinsed in 0.01% buffer B, and mounted in Vectashield Mounting Medium with DAPI. The specificity of PLA was confirmed by omitting the primary antibody.

Supplemental Tables

Table S1: Clinical information of human serum samples from patient cohort.

Patient No	Age	Sex	Diagnosis	Previous Cardiac Surgery	NYHA Classification* During Admission	LVEF (%)
1	77	Female	AS, Hypertension, Dyslipidaemia, COPD	No	3	40
2	73	Male	AS, Hypertension, CAD, COPD	No	3	40
3	90	Female	AS, Hypertension, CAD	No	3	45
4	86	Female	AS, Hypertension	No	2	55
5	80	Male	AS, CAD	No	3	35
6	91	Female	AS, Hypertension, Dyslipidaemia, CAD, PAD	No	3	60
7	75	Male	AS, Hypertension, Diabetes, Dyslipidaemia	No	3	50
8	86	Female	AS, Hypertension, COPD	No	3	45
9	83	Female	AS, Hypertension, Dyslipidaemia, CAD	No	3	65
10	76	Male	AS, CAD	No	4	30
11	84	Male	AS, Hypertension, Diabetes, Dyslipidaemia, CAD, COPD	Yes	3	40
12	85	Male	AS, Hypertension	No	3	20
13	80	Male	AS, Dyslipidaemia, CAD	No	3	25
14	76	Male	AS, Dyslipidaemia, CAD	No	3	45
15	76	Female	AS, Hypertension, Diabetes, Dyslipidaemia, CAD	No	3	40
16	82	Female	AS, Hypertension, Diabetes, CAD	No	3	65

*New York Heart Association (NYHA) Functional Classification Criteria Committee, New York Heart Association, Inc. Diseases of the Heart and Blood Vessels. Nomenclature and Criteria for diagnosis, 6th edition Boston, Little, Brown and Co. 1964, p 114.
AS: Aortic Stenosis, CAD: Coronary Artery Disease, COPD: Chronic Obstructive Pulmonary Disease, LVEF: Left ventricle ejection fraction, PAD: Peripheral Arterial Disease.

Table S2: List of primers used for genotyping of *Gadlor*-KO mouse line.

	Primer 1 (Forward)	Primer 2 (Reverse)
***Gadlor*-WT**	CTTGAGCCGTCTCTCCAAAG	GGGTGGCATGCAAGATGATTGAGA
***Gadlor*-KO**	CTTGAGCCGTCTCTCCAAAG	TGTGGAGTGGACACATAGAGG

Table S3: List of antibodies/reagents used in cell isolation methodologies and immunofluorescence (IF) staining.

Target	Company, Catalogue No	Application
**Anti-mouse CD146 (LSEC) MicroBeads**	Miltenyi-Biotec, 130-092-007	Cell isolation
**Feeder Removal MicroBeads, Mouse**	Miltenyi-Biotec, 130-095-531	Cell isolation
**Isolectin B4 (IB4)**	Vector Lab, FL-1201	1:50 (IF)
**Purified Rat Anti-mouse CD102**	BD Pharmingen, 553326	Cell isolation
**Purified Rat Anti-mouse CD31**	BD Pharmingen, 553370	Cell isolation
**Rabbit Polyclonal anti-ki67**	Abcam, ab15580	1:100 (IF)
**VECTASHIELD HardSet Antifade Mounting Medium With DAPI**	Vector Lab, H-1500	
**Wheat Germ Agglutinin (WGA)**	Invitrogen, W21405	1:100 (IF)

Table S4: List of primers used for qPCR and qRT-PCR (5´-3´).

	Primer 1 (Forward)	Primer 2 (Reverse)
Gadlor1	AGGTGAGCTCTGGTTGTGTT	CTGCTGCCTGTGAAAGATGG
Gadlor2	TGAGACTCCACTTGCCACAT	TGTGGTTTCAGGCATGTTTCT
GADLOR1	AATTTCAGCCACAAGCATCC	TGCTTGGGGAAGAGGAAGTA
GADLOR2	TGGGATCTAAGCACTGACACC	GAGACAGACATTCGTTTGGTCA
**18S**	GTAACCCGTTGAACCCCATT	CCATCCAATCGGTAGTAGCG
**U6**	CTCGCTTCGGCAGCACA	AACGCTTCACGAATTTGCGT
Gapdh	CCGCATCTTCTTGTGCAGT	CATCACCTGGCCTACAGGAT
Acat1	GCAGGGAAGTTTGCCAGTGAGA	GAACACGGTCTTGAGCTTTGGC
Actn2	CACCTGGAGTTTGCCAAGAGAG	GCCTTGAACTGCTCATGTGCAG
Adam8	TGCCAACGTGACACTGGAGAAC	GCAGACACCTTAGCCAGTCCAA
Angpt2	AACTCGCTCCTTCAGAAGCAGC	TTCCGCACAGTCTCTGAAGGTG
Angptl4	CTGGACAGTGATTCAGAGACGC	GATGCTGTGCATCTTTTCCAGGC
Aurkb	CTTCTACGACCAGCAGAGGATC	GGCATCTGACAGTTCCTCCATG
Cacna1c	CGTTCTCATCCTGCTCAACACC	GAGCTTCAGGATCATCTCCACTG
Camk2d	GTGACACCTGAAGCCAAAGACC	CCTGTGCATCATGGAGGCAACA
Cdk1	CATGGACCTCAAGAAGTACCTGG	CAAGTCTCTGTGAAGAACTCGCC
Col15a1	ACACCCACAGTGACTCCCAAGA	TCCTCATTGCCCACGATGTCTC
Col1a1	CCGCTGGTCAAGATGGTC	CCTCGCTCTCCAGCCTTT
Col3a1	ATAAGCCCTGATGGTTCTCG	ATGCATGTTTCCCCAGTTTC
Col4a1	ATGGCTTGCCTGGAGAGATAGG	TGGTTGCCCTTTGAGTCCTGGA
Col6a1	GACACCTCTCAGTGTGCTCTGT	GCGATAAGCCTTGGCAGGAAATG
Comp	GTGCCCAACTTTGACCAGAGTG	ACAGGCATCACCCACAAAGTCG
Cox5a	GTCACACGAGACAGATGAGGAG	CCGTCTACATGCTCGCAATGCA

Cxcl2	CATCCAGAGCTTGAGTGTGACG	GGCTTCAGGGTCAAGGCAAACT
Dll1	GCTGGAAGTAGATGAGTGTGCTC	CACAGACCTTGCCATAGAAGCC
Efna1	GCTGAAGGTGACTGTCAATGGC	CGGCACTGTAACCAATGCTGTG
Fgfr2	GTCTCCGAGTATGAGTTGCCAG	CCACTGCTTCAGCCATGACTAC
Fh1	GAACTCACACGCAGGATGCTGT	GGCGGCTTTTATTCTCACCATCG
Fn1	TGTGACAACTGCCGTAGACC	TGGGGTGTGGATTGACCTTG
Gata4	GCCTCTATCACAAGATGAACGGC	TACAGGCTCACCCTCGGCATTA
Icam5	ACCGATGCACAGCAGTCAATGG	ATGTTCTGGGCAGCCTACACTG
Igf1	GTGGATGCTCTTCAGTTCGTGTG	TCCAGTCTCCTCAGATCACAGC
Il6	CGGCCTTCCCTACTTCACAA	TCCAGTTTGGTAGCATCCATCA
Klf15	ACACCAAGAGCAGCCACCTCAA	GCCTTGACAACTCATCTGAGCG
Lsamp	GGAGTCGAAGAGCAACGAAG	AATCTCAAGGCCATTTGCAC
Mfn2	GTGGAATACGCCAGTGAGAAGC	CAACTTGCTGGCACAGATGAGC
Myh6	GAGTGGGAGTTTATCGACTTCG	CCTTGACATTGCGAGGCTTC
Myh7	ACTGTCAACACTAAGAGGGTCA	TTGGATGATTTGATCTTCCAGGG
Nppa	TTCCTCGTCTTGGCCTTTTG	CCTCATCTTCTACCGGCATC
Nppb	GTCCAGCAGAGACCTCAAAA	AGGCAGAGTCAGAAACTGGA
Rcan1.4	CTTGTGTGGCAAACGATGATG	TGGTGTCCTTGTCATATGTTCTG
Sirt1	GGAGCAGATTAGTAAGCGGCTTG	GTTACTGCCACAGGAACTAGAGG
Sucla2	GGTGTCTCTGTTCCCAAAGGCT	TTTCCTCTGCCGCCAGCCAAAA
Tlr9	GCTGTCAATGGCTCTCAGTTCC	CCTGCAACTGTGGTAGCTCACT
ND1	CTAGCAGAAACAAACCGGGC	CCGGCTGCGTATTCTACGTT
16S	CCGCAAGGGAAAGATGAAAGAC	TCGTTTGGTTTCGGGGTTTC
**Gadlor_Intron**	GCTGCACAAATCCCACTTCA	TGAAGTGGGATTTGTGCAGC
**Gadlor_Spanning**	AGCACATTGTACTCCCATGTT	GTGAGTCCCTGCAAGTCCTA

**Gadlor1_ExonIntron**	AGCACATTGTACTCCCATGTT	GGCAGTTTGGAAGGTGATCA
**Gadlor2_ExonIntron**	TGGGAAGTAGAGAGCTATTGTTC	TCCTATGTGTGCACTGTCCA

Table S5: List of *Gadlor1* and *Gadlor2* probes used for RNA antisense purification (RAP).

	**Probe Sequence**
***Gadlor1* – Probe 1**	[Btn]AGGATTGTTAAATATGACTATGCTTGGTATAGTCACAAAACATGGGAGTAC
***Gadlor1* – Probe 2**	[Btn]TATCATAATCTTTCTGTAGGCCATTACTTGTTTCATATTTTAAAGGGACAGTCCACTCTAGGAATGTCAAGTGTCTGATCTCTGAAAACA
***Gadlor1* – Probe 3**	[Btn]TTTTCTGAATAGTTGAAAATTCTAACTAAACACAGGAAGAACAGAGNCACAAGAATAAAGAAATTTAGATATATCCTAAATGTTTCCAGG
***Gadlor1* – Probe 4**	[Btn]ATAAAAGAAGGCGAGGGGTGGCATGCAAGATGATTGAGAAAGCCCAGTAGCCATTTTTGGGGTGGGGCAAAGGGAGTGGTCTGGGTAGGG
***Gadlor2* – Probe 1**	[Btn]GCTGCTATTTTATTATCTCTTTGGTTCTGTTTTTCATTTGTATTAAAAATG
***Gadlor2* – Probe 2**	[Btn]GTGATGGTGAAGATGAAATTGAGATGAATCATTTGAAGAACGATGTGCGTTTTAGAAGAATCACTTTGTC

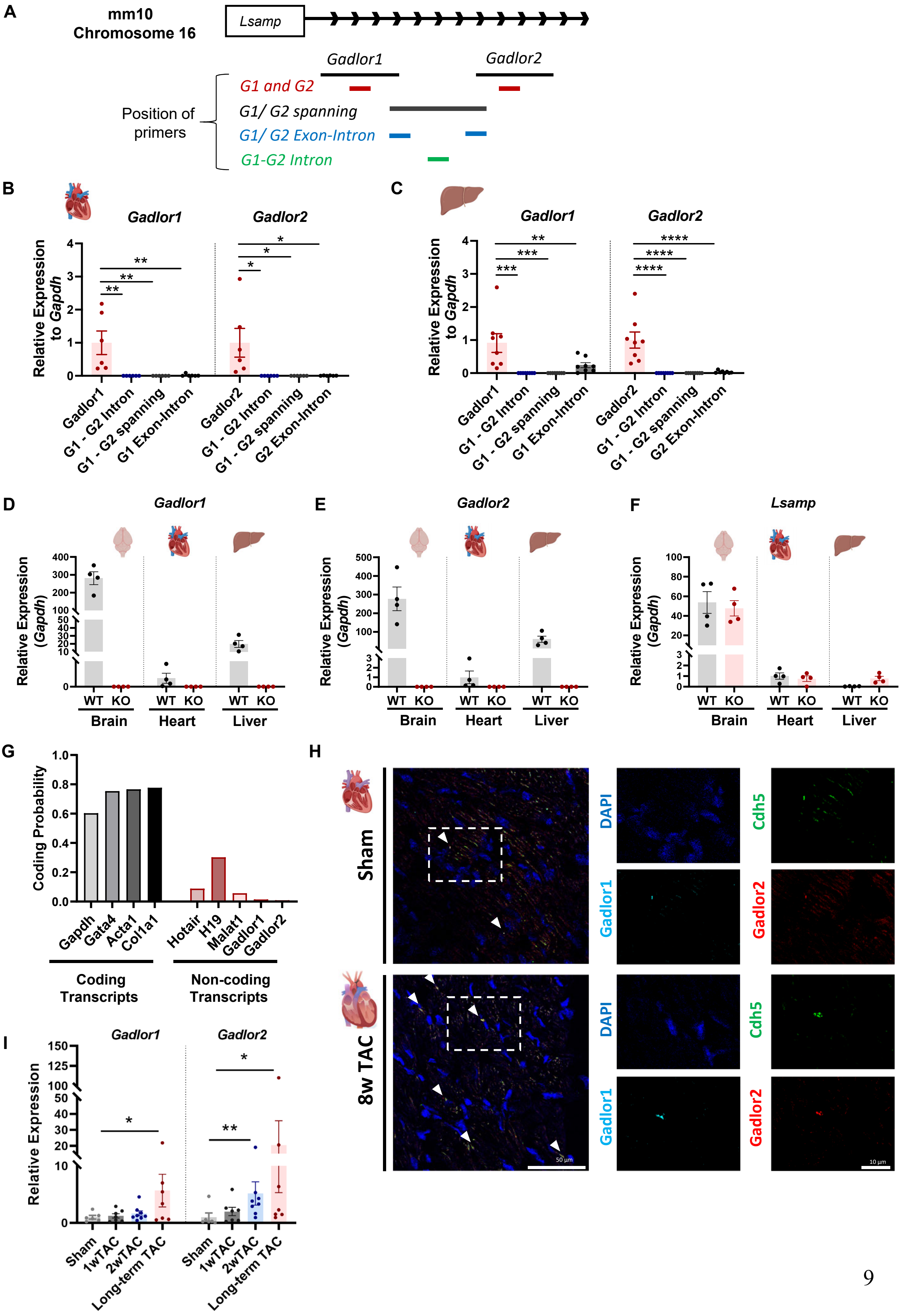
A
mm10
Chromosome 16
Lsamp
Gadlor1
Gadlor2
Position of primers
G1 and G2
G1/ G2 spanning
G1/ G2 Exon-Intron
G1-G2 Intron
B
Gadlor1
Gadlor2
Relative Expression to Gapdh
Gadlor1
G1 - G2 Intron
G1 - G2 spanning
G1 Exon-Intron
Gadlor2
G1 - G2 Intron
G1 - G2 spanning
G2 Exon-Intron
C
Gadlor1
Gadlor2
Relative Expression to Gapdh
D
Gadlor1
Relative Expression (Gapdh)
WT KO
Brain
Heart
Liver
E
Gadlor2
F
Lsamp
G
Coding Probability
Gapdh
Gata4
Acta1
Col1a1
Hotair
H19
Malat1
Gadlor1
Gadlor2
Coding Transcripts
Non-coding Transcripts
H
Sham
8w TAC
DAPI
Cdh5
Gadlor1
Gadlor2
50 µm
10 µm
I
Gadlor1
Gadlor2
Relative Expression
Sham
1wTAC
2wTAC
Long-term TAC

Figure S1: *Gadlor1* and *Gadlor2* are non-coding transcripts that are located in the intronic region of the *Lsamp* gene.
A. Position of primer pairs targeting different regions within *Gadlor1* and *Gadlor2* lncRNAs. **B-C.** Quantitative RT-PCR analysis of *Gadlor1/2* expression in heart and liver tissues with indicated primer pairs. **D-F.** Systemic deletion of the whole region containing *Gadlor* lncRNAs did not affect the expression of the neighbor gene *Lsamp*, which is mainly expressed in brain tissue (*Gapdh* expression used for normalization). Relative expression of *Gadlor1* (D), *Gadlor2* (E) and *Lsamp* (F) were shown in mouse brain, heart and liver tissue samples collected from WT and Gadlor-KO mice (n=4, same samples were indicated in each graph). **G.** Coding probability of *Gadlor1* and *Gadlor2* was evaluated with the online Coding Potential Assessment Tool (CPAT - http://lilab.research.bcm.edu/) and compared with coding transcripts and well-known lncRNAs for validation that revealed Gadlor1 and Gadlor2 are non-coding transcripts. **H.** Fluorescent in situ hybridization of *Gadlor1* (cyan), *Gadlor2* (red) and *Cdh5* RNAs in mouse heart tissue sections of sham and following 8-weeks of TAC surgery. *Cdh5* shows endothelial cells (green), nuclei stained with DAPI (blue), and the overlap in 3 channels was indicated with arrows. Dashed rectangles were the zoomed areas showed in separate pictures. Scale bars: 50 μm and 10 μm, as indicated. **I.** *Gadlor1/2* lncRNA levels were assessed in mouse cardiac ECs at different time-points post-TAC compared to sham samples. Sham group (n=6), 1-week TAC (n=7), 2-week TAC (n=8), Long-term TAC (10-12 weeks, n=7). The housekeeping gene 18S was used for normalization of RT-qPCR data. Data are shown as mean±SEM. *p-value<0.05, **p-value<0.01, ***p-value<0.001; ****p-value<0.0001

A **AK037972 (*Gadlor1*)**

```
#=======================================
# Aligned_sequences: 2
# Gap_penalty: 10.0
# Extend_penalty: 0.5
#
# Length: 578
# Identity:     268/578 (46.4%)
# Similarity:   268/578 (46.4%)
# Gaps:         259/578 (44.8%)
# Score: 740.5
#=======================================

H    1 AAATTGTCTAGAA-TGTGGCAGTTTAAAAACACATCTACATTGATCTACA     49
         |||||||.||||| |||||||.|||||||.|||.||..|||||||||||.
M    1 AAATTGTTTAGAACTGTGGCACTTTAAAAGCACCTCGGCATTGATCTACT     50

      50 GTCTCAAGGTCCCAC--CTTTGTGTTACTTTTAGAATTTGTAGATGGATG     97
         ..|||.||.||.|||  ||.||||.||||||||||.|||||||||||||.
      51 AACTCTAGATCTCACCTCTCTGTGCTACTTTTAGAGTTTGTAGATGGATA    100

      98 TTATTCAGCA-AGGATGACTCATCTACATT-TGGTTTGTCAATTTGTTTT    145
         .|.|.||||| |||| ||.||||||||.|| ||  .||||.|||||||||
     101 CTGTCCAGCAGAGGA-GAGTCATCTACGTTCTG--ATGTCGATTTGTTTT    147

     146 CAAAGATCATA----TGTACATAGCGTCCT----TCGATCTTACTTTCCC    187
         ||.||||||.|    || ||||    ||||    |.|     |||.||||
     148 CAGAGATCAGACACTTG-ACAT----TCCTAGAGTGG-----ACTGTCCC    187

     188 TTTAAAATATGAAACTGATAATGACCTTGAAAAAGAT-------AGAATT    230
         |||||||||||||||...|||||.|||..|.||||||       |.|.||
     188 TTTAAAATATGAAACAAGTAATGGCCTACAGAAAGATTATGATAAAACTT    237

     231 TC----------------------------------------AG------    234
         ||                                        ||
     238 TCCAGTTTTTTGGCTTTTCCCTTCGGCCCTGACTGGATGTGTAGTCTACT    287

     235 --CCACAA------------------------------------------    240
           ||||||
     288 TCCCACAATCCTGTATGTTACATGGTTCCCTACCCAGACCACTCCCTTTG    337

     241 -----------------------------------------GCATCCCA-    248
                                                  ||||.|||
     338 CCCCACCCCAAAAATGGCTACTGGGCTTTCTCAATCATCTTGCATGCCAC    387

     249 ---------------------------------TGAA-------AT----    254
                                          ||||       ||
     388 CCCTCGCCTTCTTTTATGTTTCAGAGTTGTGTGTGAAGAATCACATTAGT    437

     255 --TAATGTATCCATACTTTTAACA--CTA-----GTATAC-----TTTAT    290
           ||.|||||.|.|.||||| |||  |||     ||.|||     |||||
     438 GATACTGTATGCTTTCTTTT-ACAATCTATTTACGTTTACACATATTTAT    486

     291 CTTCTTATTT-CA-AATGCTC--TGTCTCC--------------------    316
         .||..||||| || |.|.|||  |||.|.|
     487 TTTTATATTTACATATTTCTCATTGTTTACCAGTTGGCAATAGTGATATT    536

     317 TT---------CTGTCCA--TTACTTCC    333
         ||         ||.|.||  ||||||||
     537 TTGTGAGCTGACTTTACATTTTACTTCC    564
```

B **AK038629 (*Gadlor2*)**

```
#=======================================
# Aligned_sequences: 2
# Gap_penalty: 10.0
# Extend_penalty: 0.5
#
# Length: 913
# Identity:     656/913 (71.9%)
# Similarity:   656/913 (71.9%)
# Gaps:         130/913 (14.2%)
# Score: 2336.5
#=======================================

H    1 AAAAAGAAAATTTGGA-AGATGCTCAAATAGGA-TTGATAGAGGTTCATT     48
         ||.||.|||||||||| || |||.||.|||||| ||| .||.|..|||..
M   34 AAGAATAAAATTTGGACAG-TGCACACATAGGACTTG-CAGGGACTCACA     81

      49 TTGTGTCATATTTT-CGCTTTATATGGATGTTGATATGTTCTCAGAATAA     97
         .||||||||||||| |.||||.||||.|||||||||||||.||||||| |
      82 GTGTGTCATATTTTGCACTTTTTATGAATGTTGATATGTTTTCAGAAT-A    130

      98 ATTTATAACTGCAATAGAAAATGGAATGACTATTGATTATTTTTTAGTTA    147
         |||||||||||||||.|||||..|||||.||||||||||..||||||||.
     131 ATTTATAACTGCAATTGAAAACTGAATGGCTATTGATTAAATTTTAGTTT    180

     148 CCCAAACTAATTGTCATATAAAGCTTTGTAAAT-CTTTTA-AAATGAGTA    195
         |..||||||||||.||||||||||||..||||| |||||| |||  ||.|
     181 CAAAAACTAATTGACATATAAAGCTTCATAAATCCTTTTAGAAA--AGGA    228

     196 TGGGTGTAAATTAAAAACGAATTAAAGCAGAGGCTTATAAAGACACT---    242
         ||.||.||||||||||||.|||||.||||.||||||||||| |.|||
     229 TGAGTATAAATTAAAAACTAATTACAGCAAAGGCTTATAAA-ATACTCCA    277

     243 ATCTCCCCTTCAATGGGTTATGTATTTTTGTTGTGTGTTAGGAAGATATC    292
         || |||..|||.|| ||||||.||   .||||||.||.||.||||.|.|.
     278 AT-TCCAGTTCTAT-GGTTATATA---CTGTTGTATGCTATGAAGGTGTT    322

     293 TCTTGATCTCCACACCCAAAACTCCTTCC----------AAATGAACAAA    332
         .|||..|||.|..|..||||||||||.||          |.||||||
     323 CCTTTGTCTTCTTAATCAAAACTCCTGCCATAGAATTAAATATGAAC---    369

     333 GGCCTACACACAAAAATTATTTCAATTGCTAATAGCCAGTTTTTAT----    378
                 ||.||||..|||||.||||||||||....|.|||.|||
     370 --------CATAAAAGATATTTTAATTGCTAATGATTACTTTATATATAG    411

     379 ------TTTTCAAAAGTAAATGCTTTCTTTTTTTGAAAAGTAAATATAAC    422
               |||||||.||                      |||  .||||||.
     412 TATCCCTTTTCAAGAG----------------------AGT--TTATAAG    437

     423 TATTTTAACAAAGTAAGATTTAAAAA---AAAAAACAACTCAGAAAAAAG    469
         .|||||.|...|||||.|||||||||   ||||||.|||      |.|||
     438 AATTTTTAATCAGTAATATTTAAAAATTTAAAAAAGAAC------AGAAG    481

     470 T--GCCAT------GCAG-AGATAACAGGGCTGATCTGTCTGTTATCTGC    510
         |  |||||      |||| ||| |.||      || ||.|.||||||||.
     482 TGGGCCATGTTAAGGCAGCAGA-ACCA------AT-TGCCAGTTATCTGT    523

     511 AGCCTCTATCCTTACTGTTAACAAGCCTTTTATCTTTGAAGACACTAAAC    560
         |.|||||||||||..||.|||.||||||||||.|||.|||||||||||.|
     524 AACCTCTATCCTTGATGCTAATAAGCCTTTTACCTTCGAAGACACTAAGC    573

     561 ATCTGGGATCTAAGCACTGACACCTATTAGTTACAGTGGTTTCCTTTTAC    610
         ||||||||||||||.|.||||||||||||||||||.||||||||||||||
     574 ATCTGGGATCTAAGTAGTGACACCTATTAGTTACAATGGTTTCCTTTTAC    623

     611 CTTTCTAAACTATCTGATAGATAAACCTCCAGGAATCCTACAAAATTAGG    660
         | ||||||.|.|||||||||||||||||||||||||||.||||||||.|.
     624 C-TTCTAAGCAATCTGATAGATAAACCTCCAGGAATCCCACAAAATTGGA    672

     661 CCCTTAATTGACCAAACGAATGTCTGTCTCTCTTTTTCAATCTAAATCAA    710
         ||||||||||||||||.|||||.|||||||||||||.||.|.|||||..|
     673 CCCTTAATTGACCAAATGAATGGCTGTCTCTCTTTTACAGTATAAATTGA    722

     711 AAAG-GAGTTTGCTCCTAGGAG--ATGAGATGATGTACAGTGGAAAAAAA    757
         |||| |||||||||||.|.|||  |.||.||.|..||    |||||.|||
     723 AAAGTGAGTTTGCTCCCATGAGGTAAGACATAACTTA----GGAAAGAAA    768

     758 TATTTCA-GCTGCAACTCCACTGGTAACCGTCAATGTGACCTTATAATCT    806
             ||| ||||..||||||||.|..| |.|.|.||||.||||.||||||
     769 ----TCACGCTGAGACTCCACTTGCCA-CATTAGTGTGCCCTTGTAATCT    813

     807 CCCC-----CCAATTTATTCCCA-AAACAGCTA--CCAAAGTGATCTTTC    848
         |.||     |||       |||| .||||| ||  .|||||||||..|||
     814 CACCAAAAGCCA-------CCCACCAACAG-TAGGACAAAGTGATTCTTC    855

     849 TAAAACCCACATC    861
         ||||||.||||||
     856 TAAAACGCACATC    868
```

C

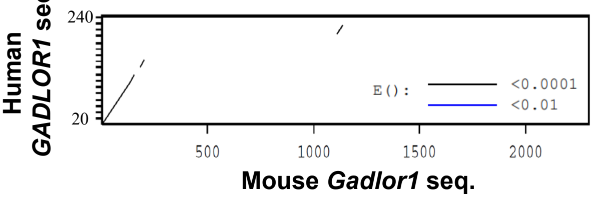

D

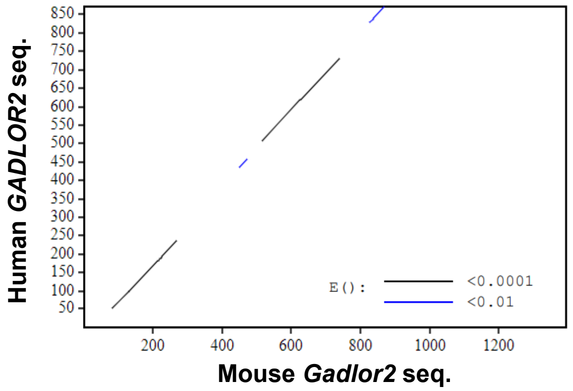

Figure S2: Conservation levels of Gadlor1/2 between mouse and human genome.
A-B. Assessment of sequence level conservation of *Gadlor1* and *Gadlor2* among human (H) and mouse (M) genome evaluated with EMBOSS-Water local alignment tool (https://www.ebi.ac.uk/Tools/psa/emboss_water/).
C-D. Visualization of pairwise local alignment with LALIGN DNA:DNA tool of University of Virginia (https://fastademo.bioch.virginia.edu/fasta_www2/fasta_www.cgi?rm=lplalign).

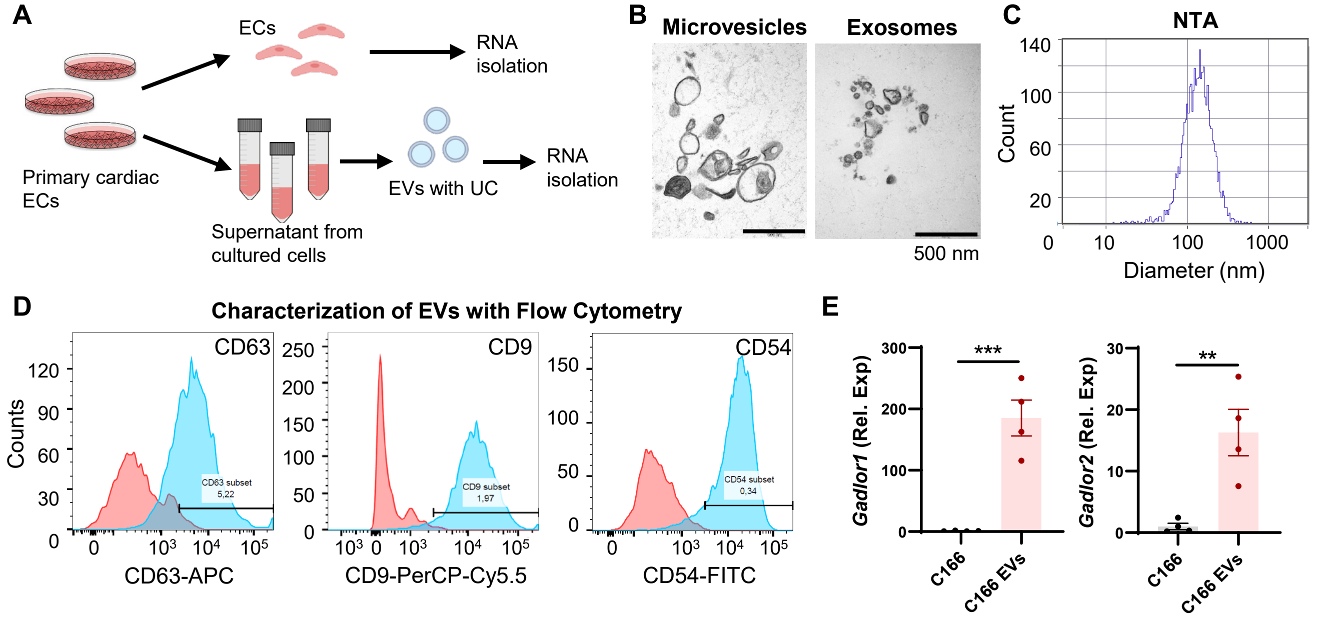

Figure S3: ***Gadlor*** **lncRNAs are mainly secreted in endothelial cells (EC) derived extracellular vesicles (EVs). A.** Scheme depicting the experimental design of EV isolation with ultracentrifugation (UC) from cultured primary cardiac ECs. Characterization of cardiac EC-derived EVs by **B.** visualizing microvesicles and exosomes with electron microscopy (scale bar: 500 nm) and **C.** detecting the size in diameter (nm: nanometers) with Nanoparticle Tracking Analysis (NTA). **D.** Flow cytometry analysis of EV surface markers (CD63-APC, CD9-PerCP-Cy5.5 and CD54-FITC) on EC-derived EVs that were stained against markers (blue curve) and compared to isotype control (red curve). **E.** Expression of *Gadlor1* and *Gadlor2* in C166 mouse endothelial cell and in EVs derived from these cells. Data are shown as mean±SEM. Data normality was evaluated with Shapiro- Wilk test and p-values were calculated with Student's t-test for parametric (or Mann-Whitney for non-parametric) assessment. *p-value<0.05, **p-value<0.01, ***p-value<0.001.

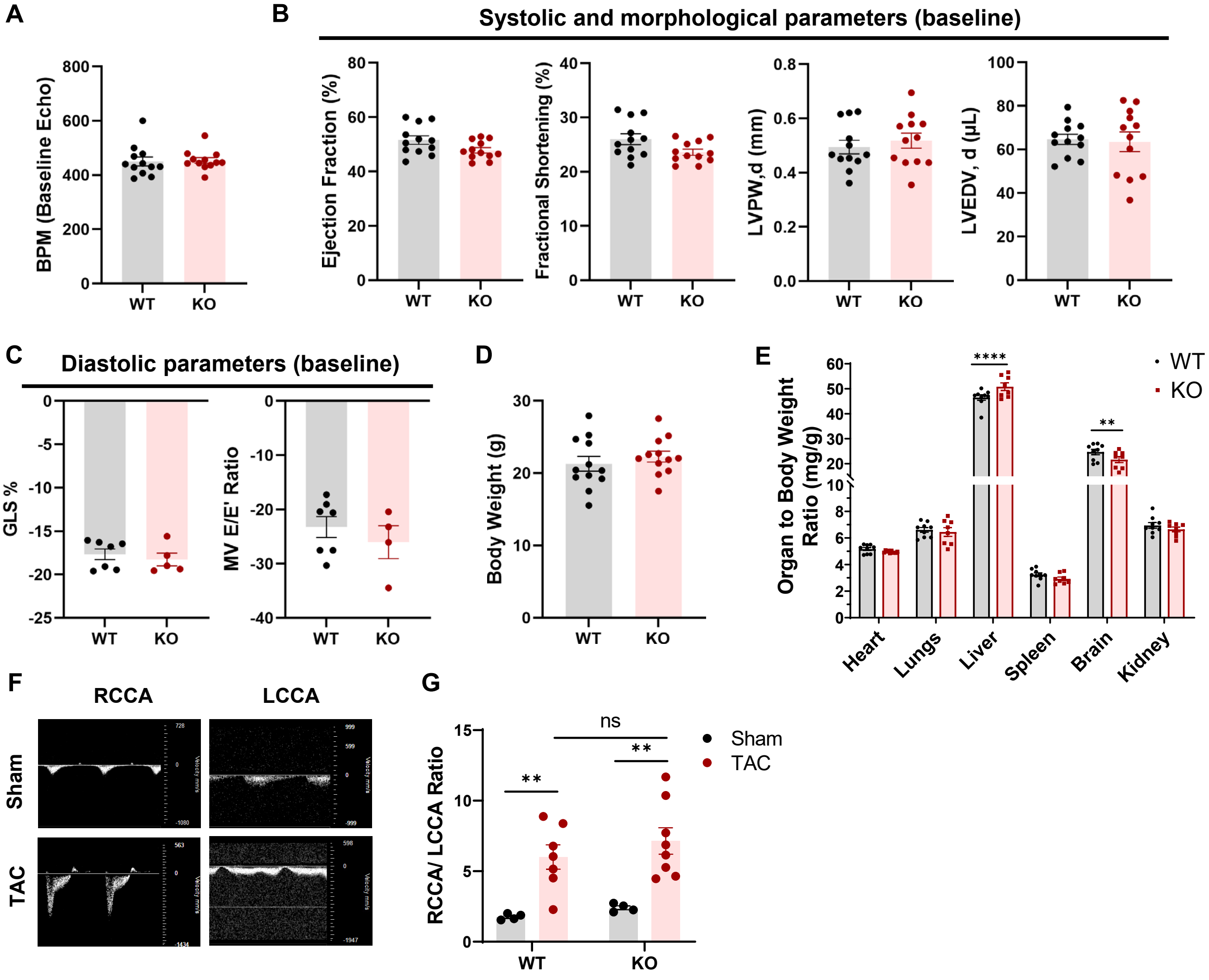

Figure S4: Baseline phenotypic characterization of *Gadlor*-KO mice compared to wild-type (WT) littermates.

A. Heart rate of WT and *Gadlor*-KO mice during echocardiography (BPM: beats per minute, n≥12). **B.** Analysis of systolic function and morphological parameters of left ventricle (LV) ejection fraction (%), fractional shortening (%), LV posterior wall thickness in diastole (mm) and LV end diastolic volume (µl) (n≥12). **C.** Analysis of diastolic function parameters, global longitudinal strain (GLS, %) and mitral valve (MV) E to E' ratio (n≥4). **D.** Body weight (g) of adult (9 weeks old) WT and *Gadlor*-KO animals (n≥18), and **E.** organ to body weight ratio (mg/g) of isolated organs including heart, lungs, liver, spleen, brain and kidney (n≥8). **F-G.** Representative images and quantification of flow measurement in right and left common carotid arteries (RCCA and LCCA) in sham (n≥4) and TAC (n≥7) mice to measure the strength of aortic constriction in WT and *Gadlor*-KO animals.

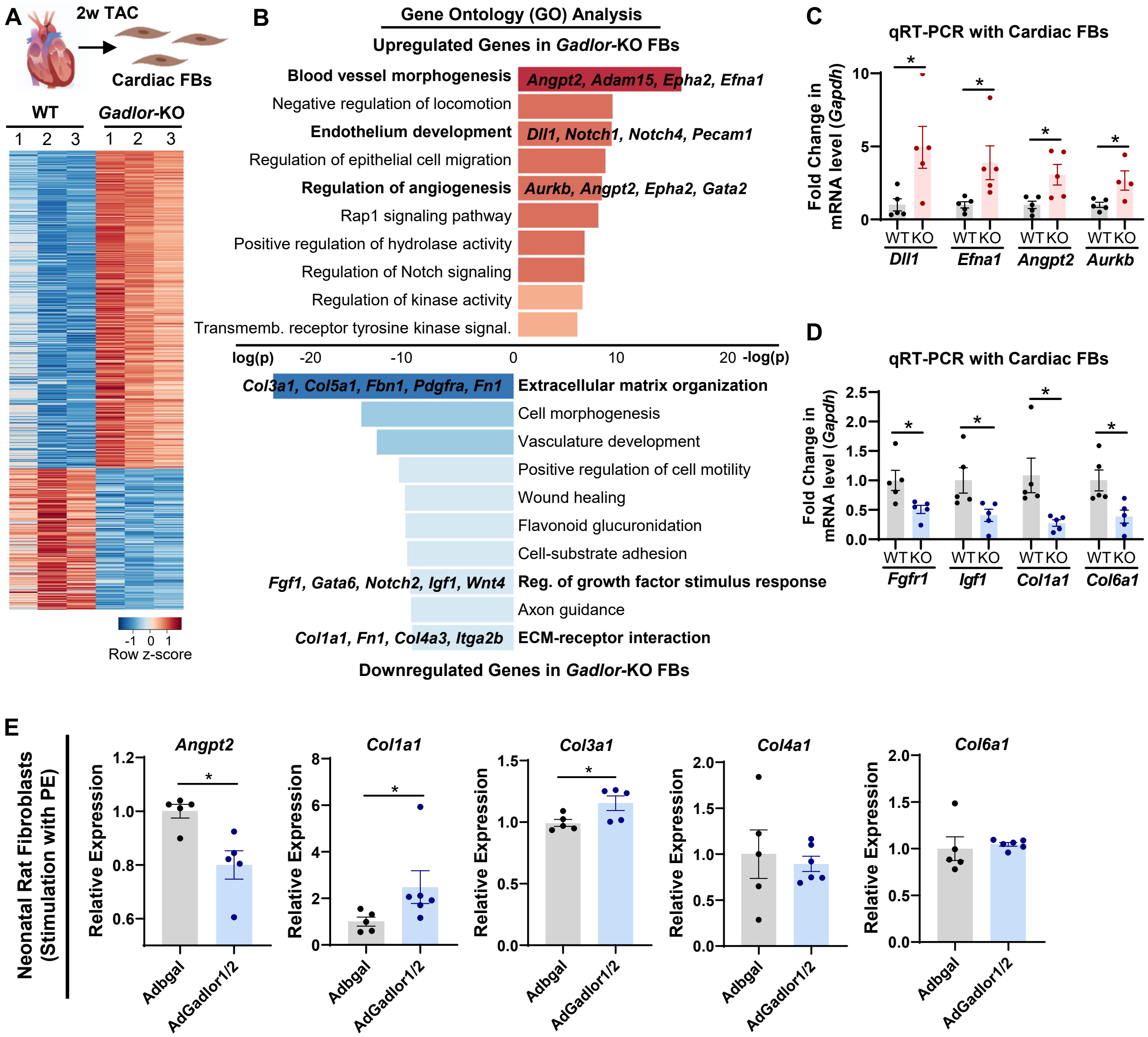

Figure S5: RNA sequencing of *Gadlor*-KO cardiac FBs showed less induction of fibrosis-associated genes after TAC.

A. Heatmap showing differentially regulated genes analysed by bulk RNAseq of isolated cardiac fibroblasts after 2-weeks TAC. **B.** Bar plots showing the gene-ontology (GO) analysis of upregulated and downregulated genes in *Gadlor*-KO FBs after 2-weeks TAC (Red: Upregulated in *Gadlor*-KO, Blue: Downregulated in *Gadlor*-KO). Exemplary genes were listed for selected GO-terms. **C-D.** Validation of selected genes from RNAseq data with qRT-PCR in isolated adult cardiac FBs after 2 weeks of TAC (Red: Upregulated in *Gadlor*-KO, Blue: Downregulated in Gadlor-KO). **E.** qRT-PCR of selected genes in neonatal rat fibroblasts (NRFB) overexpressing *Gadlor* lncRNAs after *Gadlor1/2* adenovirus treatment (βgal as control) followed by phenylephrine (PE) stimulation (100 µM, 24 hours). Data are shown as mean±SEM. Data normality was evaluated with Shapiro-Wilk test and p- values were calculated with Student's t-test for parametric and Mann-Whitney test for non-parametric assessment. *p- value<0.05.

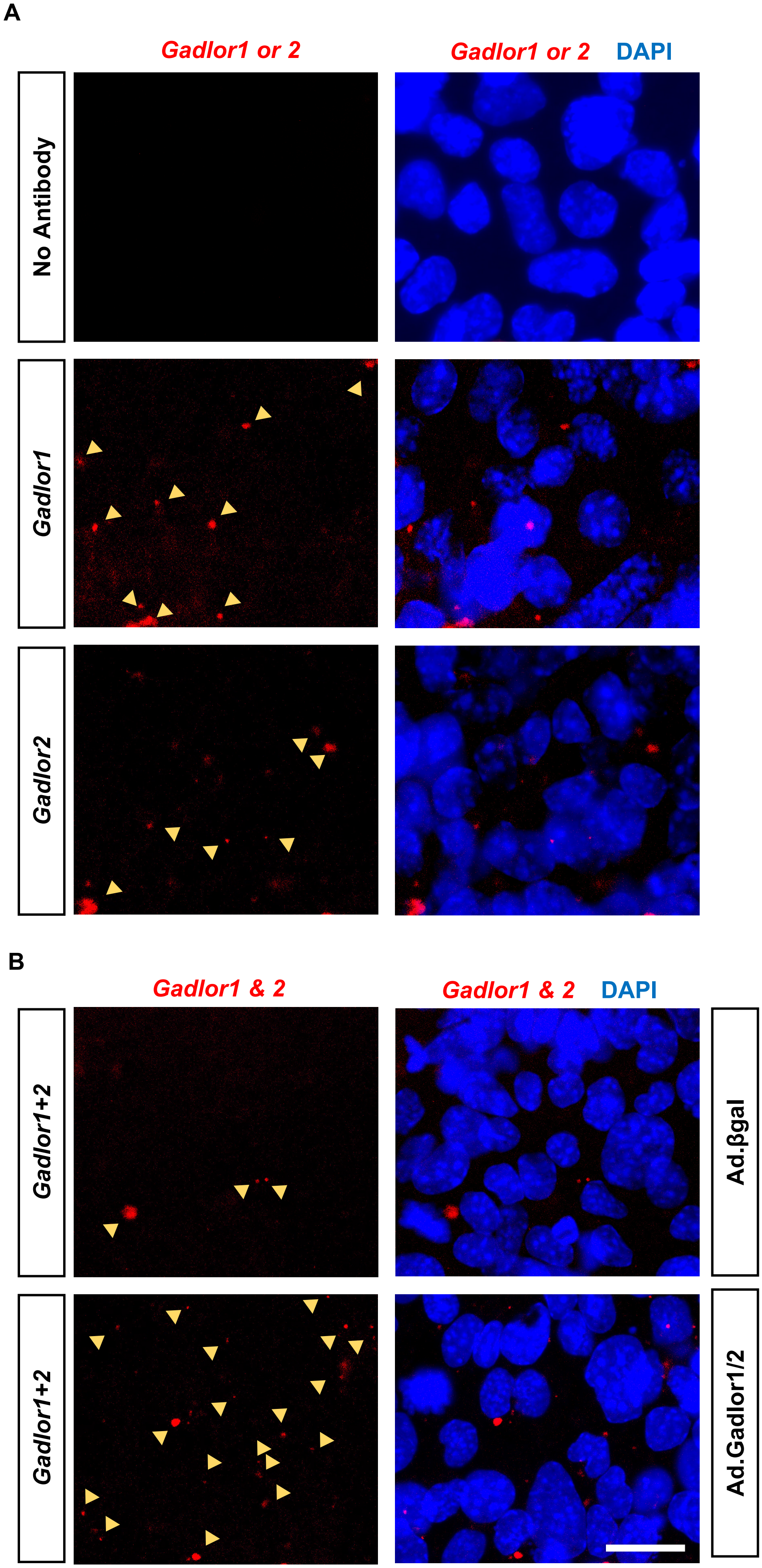
A
Gadlor1 or 2
Gadlor1 or 2 DAPI
No Antibody
Gadlor1
Gadlor2
B
Gadlor1 & 2
Gadlor1 & 2 DAPI
Gadlor1+2
Gadlor1+2
Ad.βgal
Ad.Gadlor1/2

Figure S6: Visualization of the specific interactions between CaMKII and *Gadlor1* and *Gadlor2* lncRNAs in situ by proximity ligation assay (PLA).

A. Representative images from proximity ligation assays (PLA) in HL1 cardiomyocytes in situ, showing the interaction between CaMKII with endogenous *Gadlor1* or *Gadlor2* lncRNAs (red). PLA specificity was confirmed by omitting the primary antibody as a negative control. **B.** Representative images from PLA in HL1 cardiomyocytes in situ, showing the interaction between CaMKII with adenoviral overexpressed *Gadlor1* and *Gadlor2* lncRNAs (red). Cells were mounted with Vectashield containing DAPI (blue). Scale bar: 25 µm.

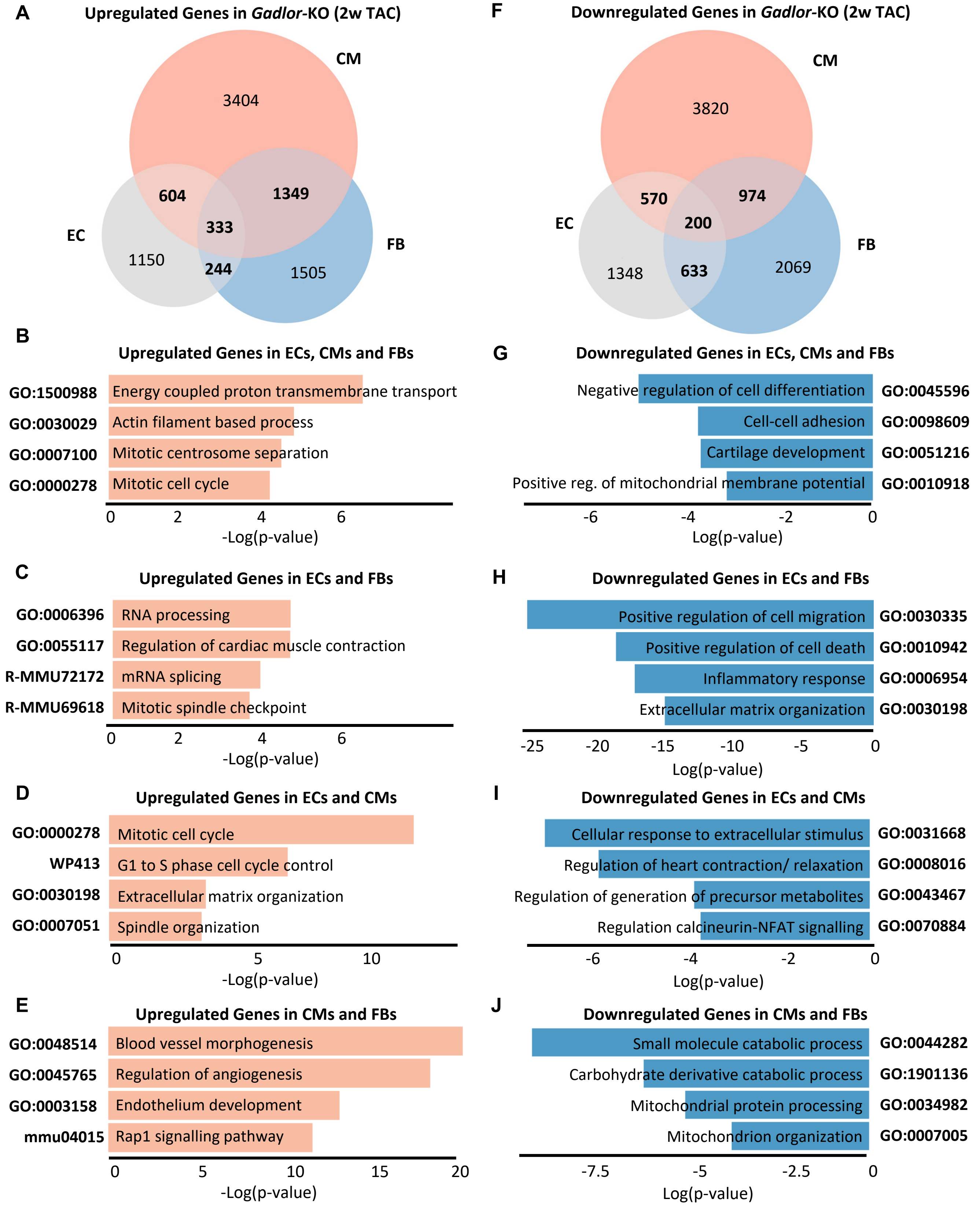

A
Upregulated Genes in Gadlor-KO (2w TAC)
CM
EC
FB
3404
604
1349
333
1150
244
1505
F
Downregulated Genes in Gadlor-KO (2w TAC)
CM
EC
FB
3820
570
974
200
1348
633
2069
B
Upregulated Genes in ECs, CMs and FBs
GO:1500988 Energy coupled proton transmembrane transport
GO:0030029 Actin filament based process
GO:0007100 Mitotic centrosome separation
GO:0000278 Mitotic cell cycle
0 2 4 6
-Log(p-value)
G
Downregulated Genes in ECs, CMs and FBs
Negative regulation of cell differentiation GO:0045596
Cell-cell adhesion GO:0098609
Cartilage development GO:0051216
Positive reg. of mitochondrial membrane potential GO:0010918
-6 -4 -2 0
Log(p-value)
C
Upregulated Genes in ECs and FBs
GO:0006396 RNA processing
GO:0055117 Regulation of cardiac muscle contraction
R-MMU72172 mRNA splicing
R-MMU69618 Mitotic spindle checkpoint
0 2 4 6
-Log(p-value)
H
Downregulated Genes in ECs and FBs
Positive regulation of cell migration GO:0030335
Positive regulation of cell death GO:0010942
Inflammatory response GO:0006954
Extracellular matrix organization GO:0030198
-25 -20 -15 -10 -5 0
Log(p-value)
D
Upregulated Genes in ECs and CMs
GO:0000278 Mitotic cell cycle
WP413 G1 to S phase cell cycle control
GO:0030198 Extracellular matrix organization
GO:0007051 Spindle organization
0 5 10
-Log(p-value)
I
Downregulated Genes in ECs and CMs
Cellular response to extracellular stimulus GO:0031668
Regulation of heart contraction/ relaxation GO:0008016
Regulation of generation of precursor metabolites GO:0043467
Regulation calcineurin-NFAT signalling GO:0070884
-6 -4 -2 0
Log(p-value)
E
Upregulated Genes in CMs and FBs
GO:0048514 Blood vessel morphogenesis
GO:0045765 Regulation of angiogenesis
GO:0003158 Endothelium development
mmu04015 Rap1 signalling pathway
0 5 10 15 20
-Log(p-value)
J
Downregulated Genes in CMs and FBs
Small molecule catabolic process GO:0044282
Carbohydrate derivative catabolic process GO:1901136
Mitochondrial protein processing GO:0034982
Mitochondrion organization GO:0007005
-7.5 -5 -2.5 0
Log(p-value)

Figure S7: Venn diagrams of differentially expressed genes in different cardiac cell types of *Gadlor*-KO mice compared to WT littermates after 2 weeks of TAC.

A. Venn diagram of upregulated genes in different cardiac cell types (CM: red, EC: grey, FB: blue) of *Gadlor*-KO mice compared to WT after 2-weeks TAC. The numbers indicating the number of genes in corresponding cell types. Genes were filtered as fold-change greater or equals to 1.2 for each condition. **B-E.** Gene ontology (GO) analysis of indicated intersections representing the overlapping upregulated genes in shown cell type of *Gadlor*-KO compared to WT littermates. **F.** Venn diagram of downregulated genes in different cardiac cell types (CM: red, EC: grey, FB: blue) of *Gadlor*-KO mice compared to WT after 2-weeks TAC. **G-J.** Gene ontology (GO) analysis of indicated intersections representing the overlapping downregulated genes in shown cell type of Gadlor-KO compared to WT littermates. Bar plots showing selected GO-terms for each condition.

Supplemental Video

Video S1: PKH67 labelled extracellular vesicles (EVs) taken up by a neonatal rat cardiomyocyte (NRCM) in a three-dimensional view.

Supplemental References

1. Grund A, Szaroszyk M, Korf-Klingebiel M, Malek Mohammadi M, Trogisch FA, Schrameck U, Gigina A, Tiedje C, Gaestel M, Kraft T, et al. TIP30 counteracts cardiac hypertrophy and failure by inhibiting translational elongation. EMBO Mol Med. 2019;11:e10018. doi: 10.15252/emmm.201810018

2. Wardman R, Keles M, Pachkiv I, Hemanna S, Grein S, Schwarz J, Stein F, Ola R, Dobreva G, Hentze MW et al. RNA-Binding Proteins Regulate Post-Transcriptional Responses to TGF-β to Coordinate Function and Mesenchymal Activation of Murine Endothelial Cells. Arterioscler. Thromb. Vasc. Biol. 2023; 43:1967–1989. doi.org/10.1161/ATVBAHA.123.319925
